# New roles for AP-1/JUNB in cell cycle control and tumorigenic cell invasion via regulation of cyclin E1 and TGF-β2

**DOI:** 10.1186/s13059-022-02800-0

**Published:** 2022-12-09

**Authors:** Beatriz Pérez-Benavente, Alihamze Fathinajafabadi, Lorena de la Fuente, Carolina Gandía, Arantxa Martínez-Férriz, José Miguel Pardo-Sánchez, Lara Milián, Ana Conesa, Octavio A. Romero, Julián Carretero, Rune Matthiesen, Isabelle Jariel-Encontre, Marc Piechaczyk, Rosa Farràs

**Affiliations:** 1 Centro de Investigación Príncipe Felipe, Valencia, Spain; 2 Present Address: PerkinElmer Informatics, Tres Cantos, Madrid, Spain; 3 Department of Pathology, Faculty of Medicine and Dentistry, Universitat de València, Valencia, Spain; 4 INCLIVA Biomedical Research Institute, 46010 Valencia, Spain; 5 Spanish National Research Council, Institute for Integrative Systems Biology, Paterna, Valencia, Spain; 6 Microbiology and Cell Science, University of Florida, Gainesville, FL USA; 7 Cancer Genetics Group, Josep Carreras Leukaemia Research Institute (IJC), Badalona, Barcelona, Spain; 8 Departament de Fisiologia, Facultat de Farmacia, Universitat de València, Burjassot, Valencia, Spain; 9 Computational and Experimental Biology Group, NOVA Medical School-Research, Faculdade de Ciências Médicas, Universidade NOVA de Lisboa, Lisbon, Portugal; 10 Institut de Génétique Moléculaire de Montpellier, University of Montpellier, CNRS, Montpellier, France; 11 Present address: IRCM, Institut de Recherche en Cancérologie de Montpellier, INSERM U1194, Université de Montpellier, Montpellier, France

**Keywords:** Transcriptome, ChIP-seq, AP-1/JUNB, Cell cycle, Epithelial to mesenchymal transition, Cancer progression

## Abstract

**Background:**

JUNB transcription factor contributes to the formation of the ubiquitous transcriptional complex AP-1 involved in the control of many physiological and disease-associated functions. The roles of JUNB in the control of cell division and tumorigenic processes are acknowledged but still unclear.

**Results:**

Here, we report the results of combined transcriptomic, genomic, and functional studies showing that JUNB promotes cell cycle progression via induction of cyclin E1 and repression of transforming growth factor (TGF)-β2 genes. We also show that high levels of JUNB switch the response of TGF-β2 stimulation from an antiproliferative to a pro-invasive one, induce endogenous TGF-β2 production by promoting TGF-β2 mRNA translation, and enhance tumor growth and metastasis in mice. Moreover, tumor genomic data indicate that JUNB amplification associates with poor prognosis in breast and ovarian cancer patients.

**Conclusions:**

Our results reveal novel functions for JUNB in cell proliferation and tumor aggressiveness through regulation of cyclin E1 and TGF-β2 expression, which might be exploited for cancer prognosis and therapy.

**Supplementary Information:**

The online version contains supplementary material available at 10.1186/s13059-022-02800-0.

## Background

AP-1 is a group of dimeric transcriptional complexes made up of combinations of Jun- (JUN, JUNB, and JUND), Fos- (FOS, FOSB, FRA1, and FRA2), ATF- (ATF-2, ATF-3/LRF1, ATF-4, ATF-5, ATF-6B, ATF-7, BATF, BATF-2, BATF-3, JDP2), and MAF (c-MAF, MAFA, -B, -F, -G, -K, and Nrl) multigene family members [1]. All AP-1-constituting proteins bear an alpha-helical bZIP domain containing a basic DNA-binding region adjacent to a leucine zipper dimerization motif [1]. AP-1 dimers bind to DNA at so-called TPA (12-O-tetradecanoylphorbol-13-acetate)-responsive elements (TREs, also called AP-1; 5′-TGA(G/C)TCA-3′) or cAMP-responsive elements (CREs; 5′-TGAGCTCA-3′) found in a wide range of gene promoters and transcription regulatory regions such as enhancers [1]. AP-1 physiologically regulates many fundamental cellular processes, including division, growth, differentiation, migration, death, and responses to a multitude of stresses and environmental cues. At the organism level, it is implicated in the control of many physiological functions [1].

AP-1 proteins can sometimes act as oncogenes or tumor suppressors, although AP-1 components have rarely been described as oncogenes per se. However, AP-1 is a mandatory downstream mediator of various activated oncogenes in many tumorigenic processes [2]. Moreover, these oncogenes frequently alter the abundance and/or the activity of certain AP-1 constituents to maximize their deleterious effects [1–3]. Thus, dysregulation of AP-1 can promote invasion and metastatization [4, 5], contribute to angiogenesis [6, 7], or stimulate inflammatory responses facilitating cancer development [8]. Notably, certain AP-1 proteins have been reported to exert tumor suppression actions under specific circumstances [9], underlining the complexity and versatility of the biology of this transcriptional complex.

JUNB has long been known as a transcription factor showing positive or negative transcriptional activity, depending on its target genes [10]. It is a cell cycle-regulated protein and its abundance varies depending on the cell proliferation state/stage [10]. In exponentially growing cells, JUNB level increases before cells enter the S phase and is maintained high until it dramatically drops during the G2/M transition, which leads to low JUNB abundance during M to G1 transition [11, 12]. We have previously shown that such a disappearance of JUNB in late G2 is due to its phosphorylation-dependent degradation by the ubiquitin-proteasome pathway and is essential for both proper mitosis and preservation of chromosome integrity [11, 13]. In contrast, high JUNB activity is necessary for the progression of dividing cells from S to G2 via transcriptional activation of the *cyclin A2* gene (*CCNA2*) [11, 14]. Besides this, JUNB can repress the *cyclin D1* gene (*CCND1*), an important positive regulator of the G1-to-S transition. As JUNB is a well-known antagonist of certain pro-cell division transcriptional actions of its relative JUN, it has been postulated that maintaining a low level of JUNB during M to G1 transition in dividing cells is necessary for JUN to be able to activate *CCND1* transcription to promote G1 progression [12]. Finally, as JUNB can also stimulate the cyclin-dependent kinase inhibitor 2A gene (*CDKN2A*), its constitutive expression at substantial levels leads to premature senescence of primary mouse embryo fibroblasts and reduces the proliferation of mouse 3T3 fibroblasts [15]. Overall, these observations indicate that JUNB can impact cell proliferation in various and, sometimes, contrasting manners. Yet, much remains to be done to elucidate how it regulates the cell cycle, which requires prior extensive identification of its target genes.

JUNB antitumorigenic activity has been shown in the myeloid lineage in transgenic mouse studies [16–18], which was consistent with JUNB repression observed in certain human myeloid malignancies [19–21]. Conversely, pro-oncogenic activities of JUNB have also been described in cell lines and mouse models [13, 22, 23]. Moreover, overexpression of JUNB has been shown to contribute to neoplastic development in several human cancers, including anaplastic large cell lymphoma (ALCL) [24], certain Hodgkin’s lymphomas [25], and breast and stomach cancers [26, 27]. JUNB has also been implicated in the pathogenesis and resistance to chemotherapy of malignant pleural mesothelioma, a highly aggressive human cancer [28]. Finally, JUNB has been associated with cancer progression. For example, it collaborates with the TGF-β1 (TGFB1 pathway to promote epithelial-to-mesenchymal transition (EMT), as well as for profibrotic and invasion responses in breast, lung, and kidney cancer cell lines [29–31]. These findings suggest an important role for JUNB in cancer development. However, the understanding of the molecular roles of JUNB in tumor cell proliferation and expansion remains ill-defined. Therefore, in the present study, we have investigated the genome-wide transcriptional effects of JunB in proliferating cancer cells, combined with functional assays. This led us to propose novel roles for JUNB activity in the regulation of both cell cycle and tumor progression. We show a positive role of JUNB in cell cycle progression via induction of the cyclin E1 gene (*CCNE1*) and repression of the TGF-β2 (*TGFB2*) gene, the cytokine product of the latter gene being best known for its cell division inhibitory activity [32]. Additionally, our data suggest an important role for JUNB in E2F, KRAS, AKT, and TGFB pathways, as well as in EMT. Since high TGFB2 levels are recurrently found in advanced tumors where it acts as an oncogenic factor, we also investigated whether continuous stimulation of JUNB-overexpressing cells with exogenous TGFB2 could disturb JUNB signaling with consequences for cell proliferation and tumorigenic phenotype. We demonstrate that, under these conditions, high levels of JUNB in cancer cells stimulate the production of endogenous TGFB2 protein by promoting its mRNA translation via a JUNB-dependent post-transcriptional mechanism and switch the response to TGFB2 from an antiproliferative- to a pro-invasive one in vitro. We further demonstrate that high expression of JUNB both enhances the tumorigenic and metastatic activities of U2OS cancer cells characterized by an epithelial phenotype, when these are xenografted in immunocompromised mice. In line with our observations, JUNB amplification is associated with a poorer prognosis in several types of epithelial cancers. Thus, our data point to novel functions for JUNB in the control of both normal cell cycle and tumor aggressiveness. They also postulate JUNB as a potential prognosis marker and therapeutic target in cancers overexpressing JUNB.

## Results

### Downregulation of JUNB impairs cell cycle progression from G1 to S

To address the role of JUNB in cell proliferation, we investigated the consequences of JUNB depletion on the distribution of cells in the various phases of the division cycle using U2OS cells which are one of the most widely used model for cell cycle studies and that we have previously used to study the regulation of JUNB protein levels during the G2/M transition [11, 13].

**Fig. 1:**
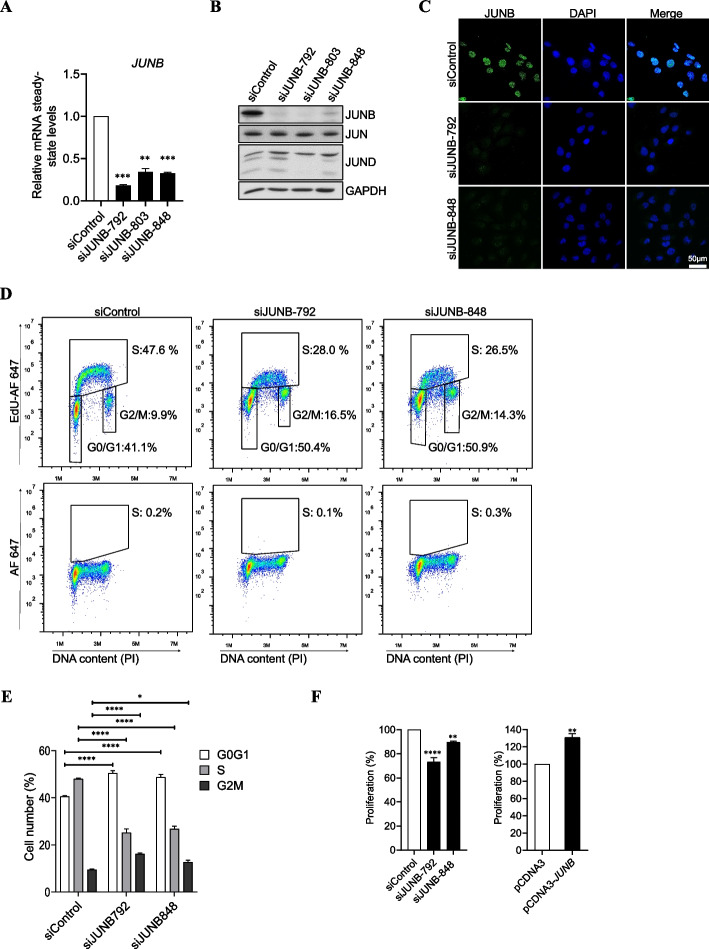
Depletion of JUNB blocks the cell cycle in G1/S and reduces entry into S phase. **A ***JUNB mRNA levels upon transfection of siRNA against JUNB* in U2OS cells. JUNB mRNA levels were analyzed by RT-qPCR following transfection of U2OS cells for 72 h with either a control siRNA (siControl) or JUNB-specific siRNAs (siJUNB-792, siJUNB-803, siJUNB-848). Data are shown as means with SEM from three independent experiments. Statistical analyses were performed by one-tailed paired *t*-test (**p<0.01, ****p<0.0001). **B ***Jun family protein levels upon transfection of siRNAs against* JUNB. JUNB, JUN, and JUND protein levels were assayed by immunoblotting in U2OS cells processed as in **A**. GAPDH was used as a loading control. **C ***Immunofluorescence analysis of JUNB in U2OS cells upon siJUNB-792 and siJUNB-848 transfection.* RNAi transfection conditions were the same as in **A**. The green color indicates positive JUNB staining, blue color indicates nuclear staining by DAPI (scale bars, 50 μm). **D ***Distribution of U20S cells in the cell cycle and DNA synthesis upon transfection of siJUNB*. RNAi transfection conditions were the same as in **A**. EdU incorporation and total DNA stained with propidium iodide were analyzed for cell cycle analysis by two-parametric flow cytometry under the conditions described in “Materials and methods.” Fluorescence intensity of cells stained with the Alexa Fluor 647 azide (AF-647) and PI is shown. Upper panel: distribution of the cells labelled with EdU in G0/G1, G2/M, and cells in S phase of a representative experiment is indicated. Lower panel: EdU unlabelled cells were included as negative controls for EdU staining. **E** Graph showing the mean of the percentage of cells in G0/G1, S, and G2/M phases of three independent experiments in **D**. Statistical analyses were performed using two-way ANOVA with Tukey’s multiple comparison test (*p < 0.05, ****p < 0.0001). **F ***Cell proliferation assay upon siJUNB- or JUNB expression plasmid transfection*. Cell proliferation was assayed using the MTS assay. Left panel: RNAi conditions were the same as in **A**. Right panel: U2OS cells were transfected with either an empty vector (*pCDNA3*) or a JUNB expression plasmid (*pCDNA3-JUNB*) for 48 h. Statistical analyses were performed by one-tailed paired *t*-test (**p < 0.01, ****p < 0.0001). Error bars represent SEM of triplicate independent experiments

First, we searched for reliable anti-JunB siRNAs. Three different siRNAs targeting various regions of the JUNB mRNA (siJUNB-792, siJUNB-803 and siJUNB-848) were designed. As a negative control, we resorted to a commercial control siRNA (siControl). All three JUNB siRNAs strongly downregulated JUNB expression at both the mRNA and the protein level, as assayed 72 h post-transfection (Fig. 1 A, B). RT-qPCR revealed that siJUNB-792 RNA displayed the highest silencing efficiency, leading to an ∼85% decrease in JUNB mRNA steady-state level (Fig. 1A). We also investigated whether the anti-JUNB siRNAs could perturb the abundances of the other JUN family proteins (JUN and JUND). Immunoblot analyses showed that siJUNB-803 affected significantly JUN and JUND levels (Fig. 1B and Additional file 1: Fig. S1A). Therefore, all further experiments were conducted with siJUNB-792 and/or siJUNB-848. In parallel, immunofluorescence assays indicated a homogeneous nuclear decrease in JUNB protein levels after transfection of U2OS cells with siJUNB-792 or siJUNB-848 (Fig. 1C).

Next, U2OS cells were subjected to RNAi for 72 h and their distribution in the cell cycle was analyzed by flow cytometry using propidium iodide-labelling of DNA, while DNA synthesis was assayed in parallel by EdU incorporation. As compared to control conditions, the cells transfected with siJUNB-792, as well as those transfected with siJUNB-848, showed an accumulation in G0/G1 associated with a strong decrease in S phase (Fig. 1 D, E). DNA synthesis was also found reduced in siJUNB-treated cells (Fig. 1D). These observations consequently pointed to a crucial role for JUNB in promoting the progression from G1 to S. Both the decrease in the fraction of cells in S and the G0/G1 arrest were also observed in epithelial cell lines derived from a uterine cervix carcinoma (HeLa), a lung adenocarcinoma (H1395), and a breast cancer (MCF-7) (Additional file 1: Fig. S1B and S1C). Furthermore, cell proliferation assays indicated 25 and 10% reductions in the number of proliferating U2OS cells 72 h after transfection with siJUNB-792 or siJUNB-848, respectively (Fig. 1F, left panel) and slower proliferation rate was confirmed by quantification of living cell time-lapse imaging (Additional file 1: Fig. S1D left panel). On the contrary, overexpression of JUNB obtained by transfection of a eukaryotic JUNB expression vector (pCDNA3-JUNB) for 48 h resulted in a 25% increase in the number of proliferating cells (Fig. 1F, right panel). Higher proliferation rate was confirmed in established U2OS-UTA6 and MCF-7 cell lines overexpressing JUNB (Additional file 1: Fig. S1D middle and right panel). Additionally, to formally establish that the changes in cell cycle arrest observed in RNAi experiments were specific to JUNB downregulation, and not caused by siRNA off-target effects, we performed JUNB rescue experiments. For this aim, U2OS cells were transfected with pCDNA3-JUNB, which expresses a JUNB mRNA insensitive to siJUNB-792, to maintain substantial JUNB protein levels in siJUNB-treated cells and their cell cycle distribution was analyzed 48 h later. Overexpression of JUNB compensated the RNAi-mediated loss of endogenous JUNB (Additional file 1: Fig. S1E, left upper panel) and cell accumulation in G0/G1 was significantly reduced (Additional file 1: Fig. S1E, left lower panel and right panel), indicating that the siJUNB-induced cell cycle arrest was indeed caused by the downregulation of JUNB.

Thus, our data indicate that JUNB exerts a positive action on cell proliferation in U2OS cells and in several other epithelial cancer types, in particular by promoting cell cycle progression from G1 to S.

### JUNB-regulated transcriptome

**Fig. 2:**
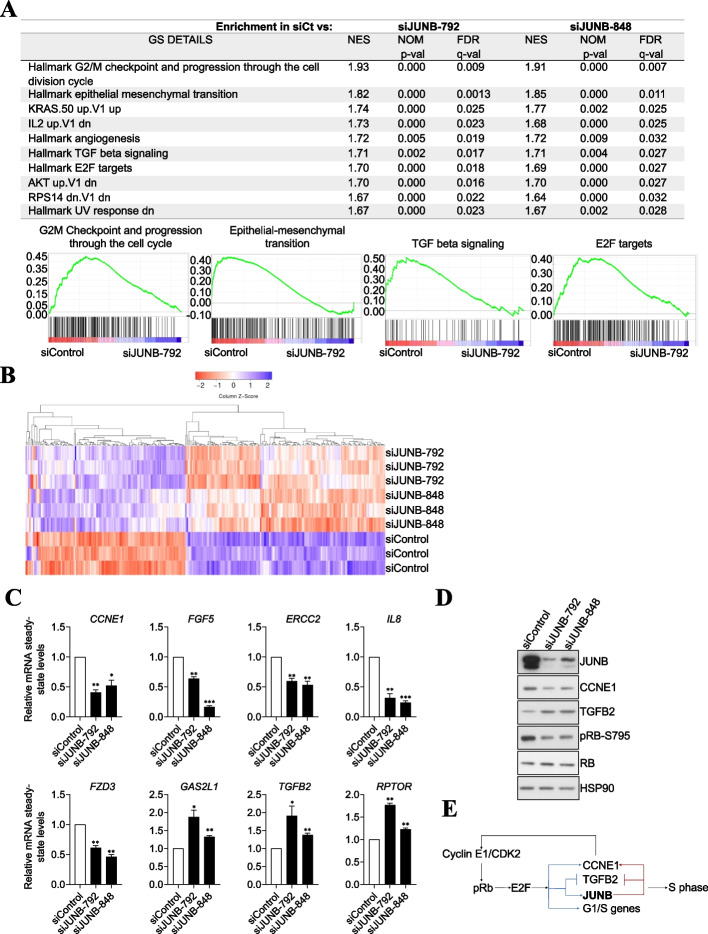
Transcriptomic analysis of JUNB knockdown in the human U2OS osteosarcoma cell line reveals that JUNB is involved in many cancer cell properties. **A ***Gene Set Enrichment Analysis (GSEA) of gene expression upon JUNB depletion*. The JUNB-regulated genes were pre-rank-ordered according to their fold change (log2) between siControl, siJUNB-792, and siJUNB-848, and analyzed based on MSigDB hallmark gene set collection [34]. The table shows gene sets upregulated in siControl vs siJUNB-792 and siJUNB-848. Gene sets are significantly enriched at FDR < 0.05. The graphs show the enrichment score (ES) plotted on the *y*-axis. The plots represent the enrichment score (ES) of the genes included in the gene sets indicated in each title, where the relative expression of each individual gene appears as a black line. The shift of genes to the left (higher expression in siControl cells) or to the right (higher expression in siJUNB-792 and siJUNB-848 cells) indicates the degree of enrichment of the signature. **B ***Heatmap hierarchical clustering of 246 consistent differentially expressed JUNB-regulated genes (FDR < 0.05)*. **C ***RT-qPCR validation of a panel of JUNB target genes involved in cell cycle regulation and cell proliferation.* The mRNA steady-state levels of the indicated genes were analyzed in U2OS cells 48h after transfection with either siControl, siJUNB-792, or siJUNB-848. The data shown are means with SEM from 3 independent experiments. Statistical analyses were performed using the one-tailed paired *t*-test (*p<0.05, **p<0.01, ***p<0.001). **D ***Depletion of JUNB leads to a decrease in CCNE1 and pRB protein levels, and to an increase in that of TGFB2*. U2OS cells were transfected with siControl, siJunB-792, or siJunB-848 72 h and protein extracts were analyzed by immunoblotting using the indicated antibodies. **E** Schematic representation of the G1/S transition regulation by E2F/pRb and JUNB

Next, to gain insights into the transcriptional network regulated by JUNB, U2OS cells were transfected with siJUNB-792, siJUNB-848, or siControl for 72 h before RNA preparation and probing of Affymetrix GeneChip Human Transcriptome 2.0 Arrays (containing mainly probes for protein-coding genes) in 3 independent experiments. In total, 1842 and 1475 transcripts were differentially up- or downregulated by a 1.25- to 10-fold factor with an FDR < 0.05 in siJUNB-transfected cells as compared to control cells (Additional file 1: Fig. S2A, Additional file 2: Table S1). The differences between the results obtained with siJUNB-792 and siJUNB-848 may be due to the fact that the former is more efficient at degrading JUNB mRNA, leading to stronger and/or faster downregulation of the JUNB protein (Fig. 1A). However, we cannot formally exclude that part of these differences might also be contributed by differences in some side-effects of the two JUNB siRNAs. Then, for siControl versus each siJUNB comparison, gene set enrichment analyses (GSEA) [33] based on hallmarks pathways were performed. The highest enrichment values were for genes involved in the following pathways (i) G2/M checkpoint and progression through the cell division cycle, (ii) EMT, (iii) TGFβ, (iv) KRAS, (v) E2F, (vi) AKT, (vii) IL2, (viii) angiogenesis, and (ix) response to ultraviolet radiation (Fig. 2A), pointing to likely pleiotropic effects of JUNB in U2OS cells. The fact that the same pathways are found downregulated with both JUNB siRNAs suggests that differences between the two mRNA silencers are more likely due to unequal silencing levels rather than to off-target effects.

Among the genes whose expression varied upon JUNB removal, 111 were upregulated and 135 were downregulated by the two JUNB siRNAs. They were called high-confidence JUNB-regulated genes (Fig. 2B, Additional file 1: Fig. S2A and Additional file 2: Table S1). Consistently with the cell cycle arrest observed upon JUNB depletion in U2OS cells (Fig. 1D), critical cell cycle regulators were found among them (Additional file 3: Table S2). In particular, *CCNE1*, which codes for cyclin E1 (CCNE1), was one of the most downregulated genes after JUNB depletion, and *TGFB2,* which codes for the cytokine TGF-β2 (TGFB2), was one of the most upregulated. Both were chosen for functional studies because the former is a crucial positive regulator of the G1/S transition and the latter a negative regulator of cell division [35, 36] (see below for more details). We also observed downregulation of other cell cycle regulators such as DNA damage repair genes (*ERCC2*), cytokines (*IL8*), growth factors (*FGF5*), and downregulation of frizzled class receptor 3 (*FZD3*), and upregulation of other genes such as *GAS2L1* and *RPTOR* (Additional file 3:Table S2). To validate our Affymetrix array transcriptomic results, we monitored the mRNA levels of these eight genes after JUNB depletion from U2OS cells in independent RT-qPCR assays (Fig. 2C). We also extended our observations to HeLa cells by analyzing *CCNE1*, *TGFB2*, *IL8*, *GAS2L1*, and *RPTOR* gene expression by RT-qPCR (Additional file 1: Fig. S2B). Additionally, we confirmed downregulation of *CCNE1* and upregulation of *TGFB2* upon JUNB silencing in H1395 and MCF-7 cells by RT-qPCR (Additional file 1: Fig. S2C and S2D). This indicated that the regulation of these genes by JUNB is conserved in epithelial cancer cells other than osteosarcoma U2OS cells.

During the G1/S transition, the kinase CDK2 in complex with basal levels of CCNE1 phosphorylates the retinoblastoma protein (pRB), leading to the activation of the transcription factors E2F1, E2F2, and E2F3, a subclass of E2F transcription factor family. In turn, these activated E2Fs stimulates the transcription of *CCNE1* and other genes encoding proteins that either promote the progression through G1/S or are required for DNA replication [35], thus creating a positive response loop that strengthens E2F activity and facilitates cell cycle progression [37]. Downregulation of CCNE1 has been shown to arrest cells in G1/S [38], to decrease pRB phosphorylation and to inhibit the E2F signaling pathway [39, 40]. Consistently with the latter observation, as well as with the G1/S cell cycle arrest undergone by JUNB-depleted U2OS cells (Fig. 1D), immunoblotting experiments indicated decreased levels of both CCNE1 and the phosphorylated form of pRB after RNAi-mediated JUNB downregulation (Fig. 2D). Similar results were observed in siJUNB-transfected MCF-7 cells (Additional file 1: Fig. S2E).

Of note, *JUNB* gene transcription has been reported to depend on E2F1, E2F2, and E2F3 (E2F1-3) activities [35]. As JUNB protein is required for CCNE1 expression (Additional file 2: Table S1), which in turn controls E2F1-3 activities, JUNB downregulation is very likely to lead to cell cycle inhibition by interfering with E2F signaling (Fig. 2A). Supporting this hypothesis, our transcriptomic data showed that JUNB and E2F1-3 share a number of common target genes involved in cell cycle regulation, one of the shared downregulated genes being *TGFB2* (Additional file 3: Table S2 and ref. [35]). As TGFB2 is known to be a negative regulator of cell division, the repression of its gene by JUNB may prevent its cytostatic effect. Indeed, we confirmed the upregulation of TGFB2 after JUNB depletion concomitantly with the downregulation of CCNE1 in U2OS and in MCF-7 cells (Fig. 2D and S2E; see functional evidence below). Taken together, these results suggested that JUNB depletion leads to cell cycle arrest in G1/S and impairs progression through S, at least in part, by interfering with the E2F signaling pathway (Fig. 2E).

We also found that several JUNB-regulated genes are involved in both TGFB signaling and EMT (Additional file 4: Table S3) with some of them also being regulated by E2F1-3 (*TGFB2*, *RBPJ*, *JAG1, CITED2*) [35, 41].

In conclusion, our transcriptomic data suggest that JUNB cooperates with and/or is a downstream effector of E2F, TGFB, AKT, and KRAS signaling and not only controls cell proliferation, but may also regulate cellular functions linked to EMT, cell invasion, and metastasis. These data also indicate that changes in JUNB levels can disturb important cell functions, such as the G1/S transition, by altering the expression of its target genes.

### Characterization of the JUNB cistrome in U2OS reveals potential direct target genes of JUNB involved in the regulation of cell cycle progression and EMT

**Fig. 3:**
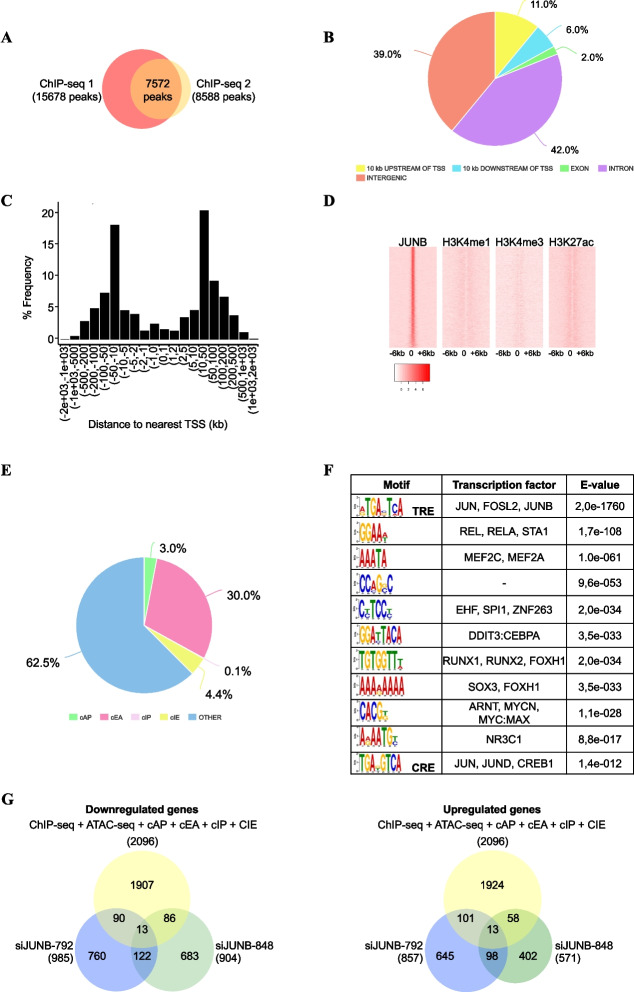
Characterization of JUNB cistrome. **A ***Proportional Venn diagram representing the intersection of JUNB binding sites identified in two independent ChIP-seq biological replicates*. **B ***Genomic distribution of JUNB binding sites*. The pie chart shows the distribution of JunB binding sites across the genome. Intergenic refers to the regions located from >10 kb from one TTS (terminal transcription site) to >10 kb of the TSS of the closest gene in 3'. **C** Distribution of the *distances of JunB peaks to the nearest transcription start site (TSS)*. **D ***Heatmaps of ChIP-seq signals around JUNB peak centers display high H3K27ac and H3K4me1 occupancy*. The heatmaps illustrate the JUNB ChIP-seq signals of U2OS in comparison to published ChIP-seq profiles of H3K4me3, H3K4me1, and H3K27ac of U2OS cells. Regions were sorted according to decreasing JUNB signal intensity (± 6kb around the centered summits). **E ***Distribution of peaks associated with JUNB binding sites either at candidate active promoter (cAP), candidate active enhancer (cAE), candidate inactive promoters (cIP), candidate inactive enhancers (cIE), and regions that do not contain histone mark specifying active or inactive enhancers or promoters (Other) as defined in the text. ***F ***Top-enriched DNA motifs identified within JUNB ChIP-seq peaks using the MEME suite*. The most enriched DNA motif corresponds to the TRE/AP-1 motif, and the less enriched DNA motif corresponds to the CRE motif. **G ***Venn diagram representing the intersection of the number of genes up- or downregulated by siJunB (siJUNB-792 and siJUNB-848; transcriptomic analysis), and genes containing JUNB binding sites annotated to the closest TSS (ChIP-seq analysis) and associated at cAP, cAE, cIP, and cIE*

As a first step to determine which of the genes whose expression varied in our transcriptomic study could be direct transcriptional targets of JUNB, we next mapped JUNB binding sites genome-wide in U2OS cells by ChIP-seq. Fragmented chromatin was immunoprecipitated with a highly specific anti-human JUNB antibody and immunoprecipitated DNA fragments were sequenced in parallel to input DNA from the same cells. Two independent biological replicates were performed to ensure that the enriched JUNB binding sites were not artifacts of sample processing and/or sequencing. The MACS peak-calling algorithm was applied to both replicates using the parameters indicated in “Materials and methods”. This led to the identification of 7572 JUNB binding peaks common to both replicates (Fig. 3A). Among these peaks, JUNB binding was more frequently observed in intergenic (39%) and intronic (42%) regions than in transcriptional promoters (defined as the 10 kb lying upstream of transcription start sites (TSSs)) of the nearest gene (11%). Moreover, JUNB binding sites were poorly represented in exons (2%), as well as in the 10-kb regions downstream of the transcription termination sites (TTSs) of the nearest gene (6%) (Fig. 3B). The distance distribution between JUNB binding sites and the closest gene TSSs, regardless of whether the former was located upstream or downstream of the TSS, is represented in Fig. 3C. About 62% of JUNB binding sites (4748/7572) were found located within 50 kb of the nearest TSSs. Our data were consistent with the currently emerging notion that AP-1-binding sites usually do not reside within gene promoters [1, 42].

We then addressed the distribution of JUNB binding sites between enhancers and promoters as described in [42]. To this aim, we intersected our JUNB cistrome data with publicly available genome-wide data obtained in U2OS cells for three histone modifications, H3K4me1, H3K4me3, and H3K27ac [43], classically used to discriminate active or inactive promoters and enhancers [42]. Heatmaps of the three histone modifications showed that the distribution of H3K4me1, H3K4me3, and H3K27ac marks was bimodal around JUNB peak centers (Fig. 3D), which is typical of regulatory elements bound by transcription factors [44]. Among the 7572 JUNB binding peaks, (i) 4257 were not surrounded by any of the three histone marks (62.3%) and were attributed no function, (ii) 2042 were surrounded mainly by H3K27ac and H3K4me1 (30%), which indicated candidate active enhancers (cAE), and (iii) 207 were surrounded by H3K27ac mainly in co-occurrence with H3Kme3 (3%), which indicated candidate active promoters (cAP) (Fig. 3E). Only 0.1% of JUNB binding peaks were found in candidate inactive promoters (cIPs; regions marked by H3K4me3 but not by H3K27ac) and 4.3% in candidate inactive enhancers (cIEs; regions marked by H3K4me1 but with low H3K27ac content and carrying neither cAPs nor cIPs) (Fig. 3E, Additional file 5: Table S4). Importantly, the majority of JunB-binding cAEs (96.4%) and cAPs (92.8%) were situated in open chromatin domains, as deduced from publicly available ATAC-seq data obtained in U2OS cells [45] (Additional file 5: Table S4). This strengthened the idea that they are genuine active regulatory elements.

De novo DNA motif analysis using the MEME suite [46] on all of the 7572 JUNB binding peaks revealed several highly enriched transcription factor (TF)-binding motifs (TFBMs). The TRE binding site showed the highest enrichment by far (Fig. 3F). It should be noted that the CRE motif appeared less enriched than any of the abovementioned TFBMs, suggesting that JUNB has lower affinity for it than for TREs in U2OS cells. Indeed, the CRE motif was found in only 20% of the JUNB peaks, whereas one or several copies of TRE were found in 90% of these peaks. The latter observation also supported the notion of direct binding of JUNB to these sites. Finally, the fact that a variety of TFBMs was found in many JUNB peaks suggested that JUNB may collaborate with other TFs at the enhancers and promoters to which it binds to regulate its target genes.

Thus, our data indicate that, besides regions for which no regulatory function could be attributed, JUNB binds more frequently to candidate active enhancers than to candidate active promoters in U2OS cells. JUNB binding to these sites is very likely direct as 90% of them contain at least one TRE motif. Finally, a majority of JUNB-bound candidate enhancers are located within 50 kb of the nearest TSSs, i.e., in a relative gene vicinity.

To search for possible direct transcriptional target genes of JUNB in U2OS cells, we then crossed our ChIP-seq- and transcriptomic data. Only the JUNB ChIP-seq peaks belonging to cAE, cAP, cIE, and cIP (2582 out of 7572) were considered for this analysis. The identified peaks were assigned by annotation to the closest gene TSS to 2096 putative target genes (Additional file 5: Table S4). Among the high-confidence gene list (246 genes), 26 genes had at least one JUNB peak suggesting that these genes may represent direct JUNB-regulated target genes (Fig. 3G, Additional file 6: Table S5 and Additional file 1: Fig. S3). Several of them (*TGFB2*, *ERCC2*, *RPTOR*, *RBPJ*, *ETV4*, *CDH4*, *CORO2B*, *TSPAN2*) being involved in cell cycle regulation and EMT (Additional file 3: Table S2 and Additional file 4: Table S3), strengthening the notion that JUNB may regulate the transcription of genes involved in the regulation of cell cycle and proliferation, TGFB signaling, and EMT. The other high-confidence JUNB-regulated genes identified in the transcriptomic assay without JUNB binding sites could be indirect target genes.

### JUNB-dependent regulation of TGFB2 during the G1-to-S transition

Studying in more detail *TGFB2* as a candidate direct transcriptional target of JUNB (Additional file 1: Fig. S3 and Additional file 6: Table S5) was of particular interest, not only because it was one of the most upregulated genes upon JUNB RNAi in U2OS cells (Additional file 2: Table S1), but also because its protein product has formerly been reported to inhibit proliferation of carcinoma cells [32] and to promote both cell invasion and cancer progression [47, 48].

**Fig. 4:**
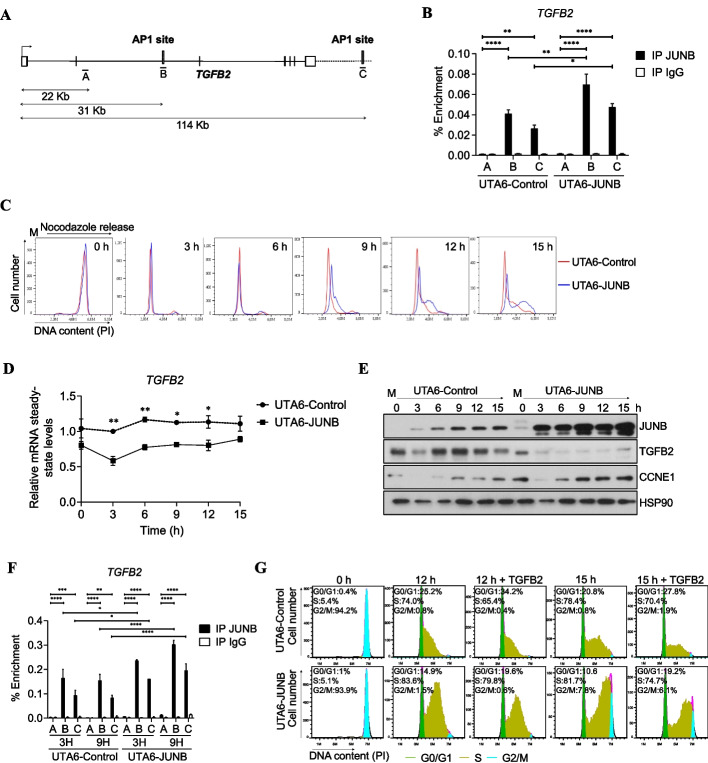
JUNB binds to and regulates the expression of TGFB2 gene. **A ***Schematic representation of the TGFB2 gene*. The position of the identified intragenic AP-1 site (**B**) and that of the AP-1 site located 114 kb downstream the TSS (**C**) are indicated. Both of them are bound by JUNB in U2OS cells. Position A, located +22 kb from the TSS, corresponds to a region not bound by JUNB and devoid of any AP-1 binding site and was used as a negative control for unspecific binding. **B ***ChIP-qPCR analysis of the enrichment of JUNB binding to site +31 kb and +114 kb of the TGFB2 locus in inducible UTA6 cells expressing pCDNA3 (UTA6-Control) and UTA6 cells overexpressing JUNB (UTA6-JUNB) cells*. Cells were grown in the absence of tetracycline, and ChIP was performed using JUNB antibody or IgG antibody as control. qPCR was carried out on the three *TGFB2* regions A, B, and C. Statistical analyses were performed using two-way ANOVA with Tukey’s multiple comparisons test (**p < 0.01, ***p < 0.001, ****p < 0.0001). **C** UTA6-Control or UTA6-JUNB were synchronized in mitosis by using a double block with thymidine and nocodazole. Cells were subjected to a thymidine block prior to the nocodazole block, and Tet was removed from the cell culture at the same time as thymidine is removed and nocodazole added to ensure substantial induction of the Tet-regulated JUNB, which requires several hours [11]. Mitotic cells were collected, released into the cell cycle, and DNA content was analyzed by flow cytometry of propidium iodide (PI)-stained cells at the indicated times (in h) as shown in the histograms. A representative experiment out of two is shown. **D ***TGFB2 mRNA levels during G1/S cell cycle progression in UTA6-control and UTA6-JUNB cell under the same conditions as described in ****C***. *TGFB2* mRNA levels were analyzed by RT-qPCR. Results are the mean of two independent experiments. Statistical analyses were performed with two-way ANOVA with Tukey’s multiple comparisons test (*p<0.05, **p<0.01). **E ***JUNB overexpression decreases TGFB2 and increases CCNE1 protein levels during cycle progression*. Protein extracts of the mitotic synchronized cells in **C** were analyzed by immunoblot using anti-JUNB, CCNE1, and TGFB2 antibodies. HSP90 and β-actin were used as loading control. **F ***ChIP-qPCR analysis of the enrichment of JUNB binding to the site located 31 and 114 kb downstream of the TGFB2 TSS in UTA6-control and UTA6-JUNB synchronized cells.* Cells were synchronized as described in **C**. Mitotic cells were collected, released into the cell cycle, and collected after 3 and 9 h for ChIP-qPCR analysis. ChIP was performed using a JUNB antibody or IgG antibody as control. qPCR was carried out on the three regions of *TGFB2 locus depicted in ****A****.* Results are the mean of two independent experiments. Statistical analyses were performed by two-way ANOVA with Tukey’s multiple comparison test (*p<0.05, **p < 0.01, and ****p < 0.0001). **G** Addition of exogenous TGFB2 ligand impaired cell cycle progression *in UTA6-Control and UTA6-JUNB cells*. Inducible UTA6-Control and UTA6-JUNB cells were synchronized in mitosis as in **E**. Mitotic cells were collected, released into the cell cycle, and concomitantly treated with 8 ng/ml of exogenous TGFB2 ligand for up to 15 h. Histograms of a representative experiment out of two are shown

Our ChIP-Seq data indicated 2 JUNB peaks located in open chromatin domains defined by ATAC-seq at the TGFB2 locus associated with histone marks. One was located at +31 kb. It contained both a TRE and a CRE motif and fell in a cIE. The other JUNB peak lied at +114 kb. It contained a single TRE motif and fell in a cAE (Additional file 6: Table S5, Fig. 4A and Additional file 1: Fig. S4A). Unless the +31 kb JUNB-bound cIE has no effect on TGFB2 expression, this raised the possibility that the transcriptional regulation of TGFB2 might involve a delicate balance between negative and positive regulatory elements.

ChIP-qPCR were conducted to validate the binding of JUNB to these two *TGFB2* gene most proximal sites (Fig. 4A). To this aim, as well as to further study the effect of JUNB on TGFB2 expression, we resorted to a U2OS tetracycline (Tet)-off cell system, expressing either inducible JUNB (UTA6-JUNB) or empty vector (UTA6-Control) we formerly engineered [11, 13]. Binding of JUNB at +31 and +114 kb was observed in both cell lines, when grown asynchronously in the absence of Tet, nevertheless with stronger signals in UTA6-JUNB cells where the JUNB gene was overexpressed (Fig. 4B). Concomitantly to higher binding of JUNB, lower TGFB2 mRNA and protein levels were observed in JUNB-overexpressing cells (Additional file 1: Fig. S4B and S4C). Lower TGFB2 levels were also observed in MCF-7 cells overexpressing JUNB-GFP (Additional file 1: Fig. S4D). In contrast, higher levels were observed in siJUNB-transfected MCF-7 cells (Additional file 1: Fig. S2E). Together with our JUNB transcriptomic data, this strengthened the idea of *TGFB2* being a directly repressed transcriptional target of JUNB.

Next, we addressed whether JUNB could act as a repressor of TGFB2 specifically during the G1-to-S transition and whether this could contribute to cell proliferation regulation. To answer this question, we first arrested UTA6-control and UTA6-JUNB cells in mitosis and, after release in the cell cycle, analyzed both progression through the cell cycle and TGFB2 expression. UTA6-JUNB cells overexpressing JUNB were found to reach S phase faster than cells expressing endogenous levels of JUNB (UTA6-Control) (Fig. 4C and Additional file 1: Fig. S4E; see time points 9 to 15 h). This result was consistent with the higher proliferation rate of asynchronously growing UTA6-JUNB cells overexpressing JUNB in the proliferation assays presented in Additional file 1: Fig. S1D (middle panel). Moreover, TGFB2 mRNA and protein levels were found lower in JUNB-overexpressing cells than in control cells at all times analyzed (Fig. 4 D, E). Of note, TGFB2 mRNA and protein levels were lower in M-arrested UTA6-JUNB than in UTA6-Control cells. We interpreted this observation to result from the higher basal level of JUNB at time 0 in the former cells (Fig. 4E), due to the cell synchronization- and JUNB expression induction procedures used. Also remarkable, the extent of protein-level reduction (Fig. 4E) was much stronger than that of its mRNA (Fig. 4D) in UTA6-JUNB cells, suggesting that JUNB can alter TGFB2 protein production, not only via a direct effect on its encoding gene, but also indirectly via influencing post-transcriptional mechanisms involved in TGFB2 production (see below for more details). It is also interesting to underline that, at time 15 h, when a large fraction of UTA6-JUNB cells have already entered the S phase (Fig. 4C), a slight but detectable increase of TGFB2 was detected at both the RNA (Fig. 4D) and the protein level (Fig. 4E). As no such an increase was detectable in control cells (which were still essentially in G1 and early S at the same time point), this supported the idea that TGFB2 repression by JUNB largely occurs during the G1-to-S transition. Further strengthening this notion, (i) JUNB binding at +31 and +114 kb could be confirmed in both UTA6-JUNB and UTA6-Control cells during the G1/S transition, being the highest binding enrichment at +31 kb in all cases (Fig. 4F) with, however, (ii) a stronger binding in UTA6-JUNB cells (Fig. 4F); and (iii) higher JunB binding enrichment was found at +31 and +114 kb in G1-to-S-synchronized than in exponentially growing cells (Fig. 4 B, F).

Finally, we asked whether the inhibition of TGFB2 production by JUNB was important to allow cells to progress from G1 to S. To this aim, we tested whether the addition of exogenous TGFB2 could affect progression from M to S of nocodazole-synchronized UTA6-Control- and UTA6-JUNB cells. Such a treatment (i) impaired G1-to-S progression in control cells (Fig. 4G), consistently with the well-described cytostatic effect of TGFB2, and (ii), more interestingly, slowed down the accelerated M-to-S progression of UTA6-JUNB where JUNB was induced (Fig. 4G). The latter slowdown was evidenced by (i) the higher number of cells still in G0/G1 and (ii) the lower number of those in S phase seen in TGFB2-treated- versus non-treated UT6-JUNB cells both at 12 and 15 h after cells were released in the cell cycle, consistently with the cytostatic effect of TGFB2 [11].

Although we cannot discard that overexpression of JUNB may alter AP-1 dimer composition and have a global impact on gene expression and affect cell cycle progression, our data indicate that the increasing expression of JUNB during progression from G1 to S represses the expression of *TGFB2*, which would otherwise behave as a cell cycle brake. Additionally, our data support the idea that JUNB-mediated downregulation of TGFB2 synthesis implies both transcriptional and post-transcriptional mechanisms.

### JUNB-dependent regulation of CCNE1 during progression from G1 to S

**Fig. 5:**
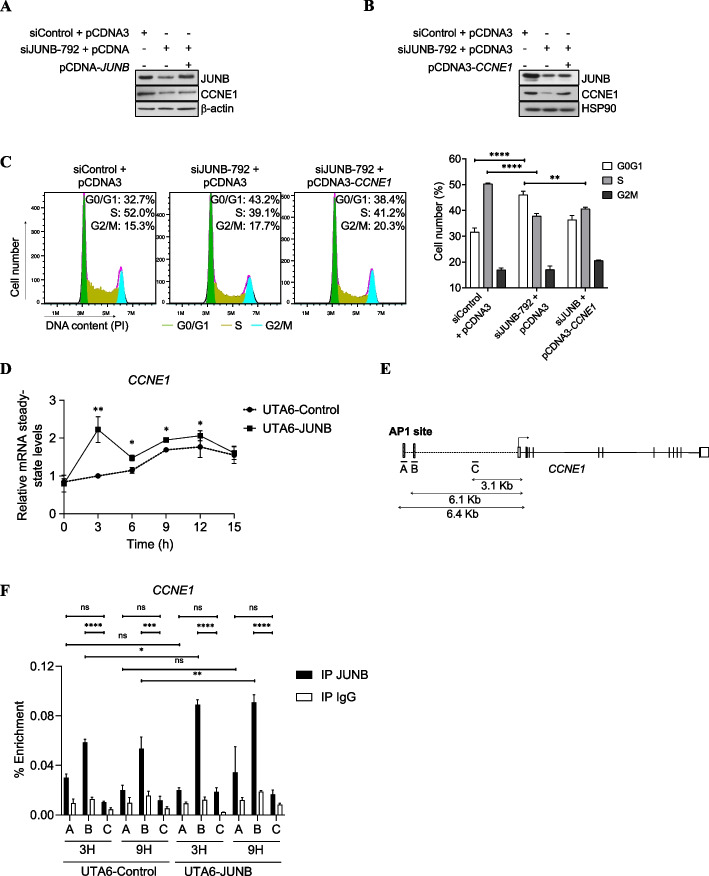
*CCNE1* is a cell cycle-regulated JUNB target. **A ***JUNB cDNA transfection in siJUNB-treated cells rescues CCNE1 protein expression*. U2OS cells were transfected with siJUNB-792 plus or minus pCDNA3 and pCDNA3-JUNB. Forty-eight hours later, protein extracts were analyzed by immunoblot using anti-JUNB and CCNE1 antibodies. β-actin was used as a loading control. **B**, **C ***CCNE1 cDNA transfection in siJUNB-transfected cells partially rescues cell cycle arrest* in U2OS cells. **B** U2OS cells were transfected with siJUNB-792 plus or minus pCDNA3 or pCDNA3*-CCNE1* and protein extracts were analyzed by immunoblot as in **A**. **C** Cell cycle of cells transfected in **B** was analyzed by propidium iodide (PI) staining of the cells. Flow cytometry profiles of a representative experiment are presented on the left panels. The right panel presents the mean of the percentage of cells in G0/G1, S, or G2/M phases of three independent experiments. Statistical analyses were performed using two-way ANOVA with Tukey’s multiple comparison test (**p < 0.01 and ****p < 0.0001. **D ***JUNB overexpression leads to an increase in CCNE1 mRNA level during G1/S cell cycle progression*. CCNE1 mRNA levels were analyzed by RT-qPCR after mitotic release as described in Fig. 4D. Results are the mean of two independent experiments. Statistical analyses were performed with two-way ANOVA and Tukey’s multiple comparisons test (*p<0.05, **p<0.01). **E ***Schematic representation of CCNE1 gene*. The positions of the identified JUNB binding sites, named A and B, each one containing one AP-1 site, and located close to the TSS are indicated. C is used as a control region for unspecific binding in ChIP-qPCR experiments. The arrow indicates the transcription start site. **F ***ChIP-qPCR analysis of the enrichment of JUNB binding to the three sites analyzed in UTA6-control and UTA6-JUNB synchronized cells. Cells were synchronized in* mitosis by double block with thymidine and nocodazole. Tetracycline was removed during the nocodazole block. Mitotic cells were collected, released into the cell cycle, and collected after 3 and 9 h for ChIP-qPCR analysis. ChIP was performed using a JUNB antibody or IgG antibody as control and qPCR was carried out on the regions A, B, and C. A representative experiment out of two is shown. Statistical analyses were performed by two-way ANOVA with Tukey’s multiple comparisons test (*p < 0.05, **p < 0.01, ****p < 0.0001)

The finding that *CCNE1* was one of the most downregulated high-confidence genes upon JUNB knockdown in our JUNB transcriptomic experiments (Additional file 2: Table S1) was interesting, due to the acknowledged role of its protein product in cell division control. We therefore formally asked whether this gene was another target whereby JUNB would actually control cell cycle progression. This was achieved in several steps. First, asynchronous U2OS cells were subjected or not to JUNB RNAi transfection and pCDNA3-JUNB (Fig. 5A), and CCNE1 protein expression were monitored. In the absence of pCDNA3-JUNB, the decrease in CCNE1 paralleled that of JUNB whereas, upon (partial) JUNB rescue, this reduction was more limited. Similar results were observed in MCF-7 cells transfected with siJUNB and pCDNA3-JUNB (Additional file 1: Fig. S5A). This strengthened the idea of *CCNE1* gene expression being dependent on JUNB. Second, an ectopic CCNE1 protein was expressed by transfection of pCDNA3-*CCNE1* plasmid into JUNB-depleted U2OS cells (Fig. 5B). This not only limited the disappearance of CCNE1, but also partially restored the ability of these cells to exit the G1 arrest (Fig. 5C), pointing to JUNB-dependent regulation of *CCNE1* as important for cell cycle control. Finally, as CCNE1 transcription is known to be tightly regulated during the G1-to-S transition [49], CCNE1 mRNA (Fig. 5D) and protein (Fig. 4E) levels were assayed in UTA6-JUNB- and UTA6-Control cells released in the cell cycle from a nocodazole block (Fig. 4C). Both CCNE1 mRNA (Fig. 5D) and protein (Fig. 4E) abundances increased in the two cell contexts during progression towards S, but more strongly in JUNB-overexpressing UTA6-JUNB- than in control cells. Moreover, increased CCNE1 mRNA and protein expression occurred earlier in the former- than in the latter cells and largely paralleled JUNB levels in both cases. This provided further evidence for an important role of JUNB-dependent regulation of CCNE1 expression in the progression of G1 cells towards S.

*CCNE1* was not identified as an obvious possible direct candidate JUNB target gene in our JUNB cistrome analysis. Nevertheless, we noted the presence of two TREs located 6.4 and 6.1 kb upstream of the *CCNE1* TSS. Interestingly, this region overlaps with H3K4me1 and H3K27ac marks and lies in an open chromatin region in U2OS cells, suggesting it to be an active enhancer region (Additional file 1: Fig. S5B). As *CCNE1* gene transcription is restricted to the limited period of the G1-to-S transition [49], we hypothesized that JUNB binding was not detected in our ChIP-seq experiments because G1/S cells represented only a minor fraction of the asynchronously growing U2OS cell population. Consequently, to address the possibility of G1/S-restricted binding of JUNB to the −6.4/−6.1-kb region, we resorted to synchronized UTA6-Control- and UTA6-JUNB cells released in the cell cycle from a nocodazole block and conducted ChIP-qPCR assays using three sets of primers. Two sets amplified the region containing the −6.4 (*A*) and −6.1 kb (*B*) TREs and one amplified a TRE-devoid domain (*C*) taken as a negative control (Fig. 5E). JUNB binding turned out to be significantly enriched at −6.1 kb TRE site in both cell lines during G1 (time point 3 h) and G1/S (time point 9 h), and, as expected, enrichment levels were stronger in UTA6-JUNB- than in UTA6-Control cells due to the higher levels of JUNB (Fig. 5F).

Thus, taken together, our data indicate that JUNB-dependent G1/S-restricted regulation of the CCNE1 protein is crucial for cells to progress from G1 to S, most probably via direct transcriptional stimulation of this gene by JUNB.

### Long-term stimulation of JUNB-overexpressing cells by exogenous TGFB2 promotes EMT and cell invasion

As mentioned earlier, advanced tumors are recurrently bathed in high concentrations of TGFB. Under this condition, TGFB signaling activation in cancerous cells is often associated with, not only a loss of cell proliferation inhibition activity, but also a switch that facilitates EMT, cell migration, invasion and, thereby, promotion of tumor progression and metastasis [50, 51]. The fact that we observed JUNB-dependent repression of TGFB2 in the experiments presented above led us to wonder about the possible disturbance of TGFB2 downstream signaling in JUNB-overexpressing tumor cells growing in a TGFB2-rich environment.

**Fig. 6:**
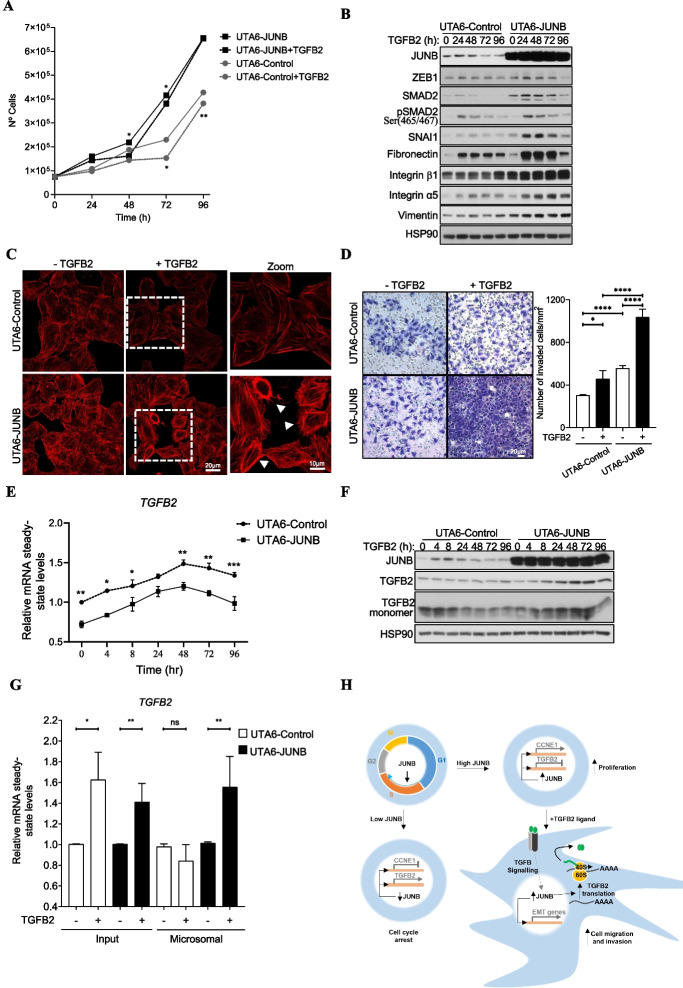
Overexpression of JUNB promotes TGFB2 signaling-induced EMT, cell invasion, and TGFB2 protein synthesis. **A** Prolonged exposure to *TGFB2 ligand exerts less antiproliferative effects*. The numbers of asynchronously growing UTA6-Control and UTA6-JUNB cells were quantified, in the absence of tetracycline and in response to increasing time of exogenous TGFB2 ligand treatment. Data are shown as means with SEM from 3 independent experiments using two-way ANOVA with Tukey’s multiple comparisons test (*p<0.05, **p<0.01). **B ***Overexpression of JUNB promotes the expression of mesenchymal proteins in cells exposed to TGFB2 ligand*. Immunoblots show the levels of JUNB, SMAD2, pSMAD2 (Ser465/467), and mesenchymal markers (SNAI1, ZEB1, Fibronectin, Integrin α5 and β2, and vimentin) in UTA6-Control and UTA6-JUNB cells asynchronously growing in the absence of tetracycline and treated with 8 ng/ml of recombinant TGFβ2 for the indicated times. HSP90 is used as a loading control. **C ***JUNB promotes a mesenchymal-like phenotype upon treatment with TGFB2 ligand.* Representative images of UTA6-Control and UTA6-JUNB cells stained with phalloidin after treatment with TGFB2 ligand for 72 h. Scale bars, 20 and 10 μm are indicated in the original and zoom images, respectively. Arrows indicate migratory cells with mesenchymal phenotype. **D ***Overexpression of JUNB increased TGFB2 ligand-induced cell migration and invasion in Matrigel invasion assays*. UTA6-Control and UTA6-JUNB cells that migrated through the membrane were stained with crystal violet, and representative fields were photographed (upper panel). Scale bars: 100 μm. Cell invasion was quantified by counting the number of cells passing through the membrane normalized to total cell number, from eleven random fields (lower panel). Data are shown as means with SEM from 3 technical replicates of 2 independent experiments. Statistical analyses were performed using two-way ANOVA with Tukey’s multiple comparisons test (*p<0.05, ****p<0.0001). **E ***Overexpression of JUNB decreases TGFB2 mRNA levels in UTA6 cells exposed to exogenous TGFB2 ligand*. Relative *TGFB2* mRNA levels in UTA6-Control and UTA6-JUNB cells treated with 8 ng/ml of recombinant TGFB2 for the indicated times were analyzed by RT-qPCR. Data are shown as means with SEM from 3 independent experiments. Statistical analyses were performed using two-way ANOVA with Tukey’s multiple comparisons test (*p<0.05, **p<0.01, ***p<0.001). **F ***Overexpression of JUNB in cells treated with TGFB2 ligand leads to an increase in endogenous TGFB2 protein*. Protein abundance of JUNB, TGFB2, and TGFB2 monomers in asynchronously growing cells in the absence of tetracycline treated with 8 ng/ml of exogenous TGFB2 were analyzed by immunoblotting. HSP90 was used as a loading control. **G ***Overexpression of JUNB induces TGFB2 mRNA association to polysome-enriched microsomal fraction, under stimulation with TGFB2 ligand.* UTA6-Control and UTA6-JUNB cells treated or not with TGFB2 ligand for 48 h were fractionated as described in the “Materials and methods.” Relative TGFB2 mRNA steady-state levels were analyzed by RT-qPCR on total RNA isolated from the whole cell extract (Input) or the polysome-enriched microsomal fraction. Data are shown as means with SEM from 3 independent experiments. Statistical analyses were performed with two-way ANOVA using Tukey’s multiple comparisons test (*p<0.05, **p<0.01; ns: non-significant). **H** Proposed model of JUNB as a promoter of cell proliferation and cell invasion. In response to mitogenic factors, JUNB protein is expressed during G1/S to G2/M to regulate cell cycle progression in proliferating cells. After long exposure to exogenous TGFB2, which are conditions found in a number of solid tumors, JUNB expression is increased and promotes TGFB2 signaling and EMT by enhancing TGFB2 protein level. This largely occurs via increasing the association of TGFB2 mRNA to polysomes leading to increased endogenous TGFB2 production. This mechanism most probably creates a positive autocrine loop increasing TGFB signaling promoting EMT, cell migration, and invasion

As a first step to address this point, we tested whether increasing the stimulation time by exogenous TGFB2 of JUNB-overexpressing U2OS cells, which would mimic permanent production of this cytokine in an advanced tumor environment, could affect cell proliferation. This turned out to be the case, as the proliferation of UTA6-JUNB cells overexpressing JUNB cultured in the presence of TGFB2 was slowed down at 48 and 72 h (Fig. 6A), in keeping with the data presented in Fig. 4G, but was no longer inhibited in a 96-h-long experiment (Fig. 6A). In contrast, TGFB2 cell proliferation inhibition activity was maintained, at 48, 72, and 96 h (although to a lesser extent), in UTA6-Control cells processed in parallel (Fig. 6A). Thus, compared to control cells, JUNB-overexpressing cells counteract more rapidly/efficiently the inhibitory effect of a long stimulation by TGFB2 on cell growth.

Next, the observation that overexpression of JUNB could contribute to the neutralization of the antiproliferative effect of exogenous TGFB2 upon long-term stimulation led us to ask whether this could be associated with a TGFB2-dependent pro-EMT phenotype. This question was also motivated by, not only the observation by others that JUNB can contribute to EMT in response to TGFB1 in breast and lung cancer cells [30, 31], but also the functional pathway enrichment analysis of our JUNB transcriptomic data pointing to a possible regulatory role for JUNB in TGFB and EMT signaling pathways (Fig. 2F). To address this point, we monitored in UTA6-control and UTA6-JUNB cells cultured for various periods of time in the presence of exogenous TGFB2, the expression of protein markers whose expression is well-known to vary during EMT induction [47] (Fig. 6B). Our data showed that in UTA6-Control cells, the addition of TGFB2 induced the expression of the endogenous JUNB gene transiently (up to 48 h) and in a moderate manner. This induction was associated with concomitant inductions of ZEB1, SMAD2, pSMAD2, SNAI1, fibronectin, and integrin α5. Additionally, slight increases in the mesenchymal markers’ integrin-β1 and vimentin were also observed at the latest time points tested. Interestingly, the induction of all of the tested EMT-associated markers was higher in JUNB-overexpressing than in control cells. It was also of note that the levels of SMAD2, SNA1, fibronectin, integrin-β1, integrin-α5, and vimentin at time 0 were also higher in UTA6-JUNB- than in UTA6-Control cells. It could be plausible that JUNB overexpression may have a global impact on gene expression by altering AP-1 dimer composition thus promoting EMT. The expression of JUNB and EMT markers were also analyzed in established MCF-7 cells expressing JUNB-GFP (MCF-7-JUNB) or GFP (MCF-7-Control) treated with exogenous TGFB2 with similar results (Additional file 1: Fig. S6A). MCF-7 cells are positive for epithelial markers, such as E-cadherin. As expected, TGFB2 treatment induced a higher decrease in the expression of E-cadherin in the MCF7-JUNB cells compared to control cells. These observations supported the idea that high JUNB expression might potentiate exogenous TGFB2-dependent EMT.

Then, we tested whether long exposure to TGFB2 could induce a mesenchymal-like morphotype in UTA-Control- and/or UTA6-JUNB cells. To this aim, filamentous actin staining with phalloidin was used to assess potential morphology changes. Untreated UTA6-Control cells showed cuboidal-shaped and in close contact but, in the presence of TGFB2 for 72 h, they became more loosely arranged and more elongated. In contrast, non-treated JUNB-overexpressing UTA6-JUNB cells showed an elongated shape with less tight connections. Moreover, this phenotype was amplified in the presence of TGFB2 with even less inter-cell connections and an even more elongated morphology associating with visible cytoplasmic extensions (Fig. 6C). This further supported the idea that, in the presence of exogenous TGFB2, JUNB can promote a mesenchymal-like phenotype.

As EMT often correlates with increased invasion ability, we next conducted in vitro invasion assays in Matrigel (Fig. 6D). UTA6-JUNB cells overexpressing JUNB showed more invasive behavior when grown in the presence of exogenous TGFB2 than in its absence. They also showed more invasive than UTA6-Control cells grown in the presence of TGFB2.

Thus, altogether, our data indicated that long-term exposure to exogenous TGFB2, as occurs in a number of advanced tumors, promotes a molecular switch in JUNB-overexpressing cells, which both counteracts the antiproliferative action of this cytokine and promotes EMT at the molecular, morphological, and functional levels.

### Regulation of endogenous TGFB2 production by JUNB in the presence of exogenous TGFB2

To clarify the apparent contradiction between JUNB-induced repression of TGFB2 gene, on the one hand (Fig. 4 D, E and Additional file 1: Fig. S4C and S4D), and JUNB acting as a promoter of TGFB2-dependent EMT, on the other hand (Fig. 6 B–D), we hypothesized that, upon long stimulation with exogenous TGFB2, the repressive action of JunB on the endogenous *TGFB2* gene could be reduced. In this scenario, exposure of JUNB-overexpressing cells to exogenous TGFB2 should trigger endogenous TGFB2 production which would then promote sustained EMT. To test it, we monitored *TGFB2* mRNA levels by RT-qPCR in UTA6-Control- and UTA6-JUNB cells 96 h after addition of exogenous TGFB2 to the culture medium. *TGFB2* mRNA accumulation was found to be transiently stimulated with a peak by 48 h post-addition of exogenous TGFB2 in both cell lines. However, *TGFB2* mRNA levels were always lower in UTA6-JUNB- than in UTA6-Control cells, including at time 0 (Fig. 6E). This supported the idea that JUNB kept at least some of its transcriptional repression ability on the *TGFB2* gene under the conditions studied.

Then, we addressed endogenous TGFB2 protein production. TGFB2 is produced as a precursor preprotein (48 kDa) that is proteolytically processed to give both a so-called latency-associated peptide (LAP) and the mature TGFB2 cytokine. This allows the formation of TGFB2 homodimers (each monomer being 12.5 kDa) bound by disulfide bridges that remain in complex with LAP (and possibly other proteins) until they are secreted outside of cells where they dissociate from LAP and, thereafter, play their biological role [52]. The kinetic analysis of the TGFB2 precursor preprotein by immunoblotting (Fig. 6F) indicated no detectable changes in UTA6-Control cells despite the observed transient induction of the TGFB2 mRNA (Fig. 6E). In contrast, a slight transient increase in the TGFB2 precursor preprotein as well as in the mature monomeric form (Fig. 6F) paralleling the profile of *TGFB2* mRNA accumulation (Fig. 6E) was seen in UTA6-JUNB cells. In addition, we assessed endogenous TGFB1 gene and protein expressions according to its activity as an inducer of EMT. In the kinetic analysis of *TGFB1* mRNA after exogenous addition of TGFB2 only subtle changes were transiently observed (Additional file 1: Fig. S6B). At the protein level, the kinetics of the TGFB1 precursor preprotein and mature monomeric form between UTA6-Control and UTA6-JUNB cells were similar, although an increase of TGFB1 protein was observed in UTA6-JUNB cells (Additional file 1: Fig. S6C).

The observation of the increment in TGFB2 precursor and mature protein levels were paradoxical, as they contrasted with the lower abundance of the *TGFB2* mRNA in UTA6-JUNB cells. This led us to inquire about the possibility of JUNB-regulated translation of the *TGFB2* mRNA in the latter cells. To this aim, we quantified *TGFB2* mRNA abundance in polysome-enriched microsomal fractions prepared from UTA6-Control- and UTA6-JUNB cells cultured in the absence or in the presence of TGFB2 ligand for 48 h (i.e., the time corresponding to maximal accumulation of *TGFB2* mRNA in UTA6-Control and UTA6-JUNB cells; Fig. 6E). No change in *TGFB2* mRNA association was seen in the former cells whereas an increase was clearly detected in JUNB-overexpressing UTA6-JUNB cells due to TGFB2 treatment (Fig. 6G).

Thus, sustained exposure of U2OS cells to exogenous TGFB2 reverses the action of JUNB on the production of the endogenous TGFB2 protein from a negative to a positive effect. However, increased production of the endogenous TGFB2 cytokine appears not to be due to loss of ability to repress the synthesis of the *TGFB2* mRNA by JUNB. Rather, it results from a TGFB2-induced, JUNB-dependent signaling facilitating *TGFB2* mRNA translation via increased recruitment in polysomes (See proposed model of JUNB as a promoter of cell proliferation and cell invasion in Fig. 6H).

### Overexpression of JUNB promotes tumor development and metastasis by U2OS cells in vivo

**Fig. 7:**
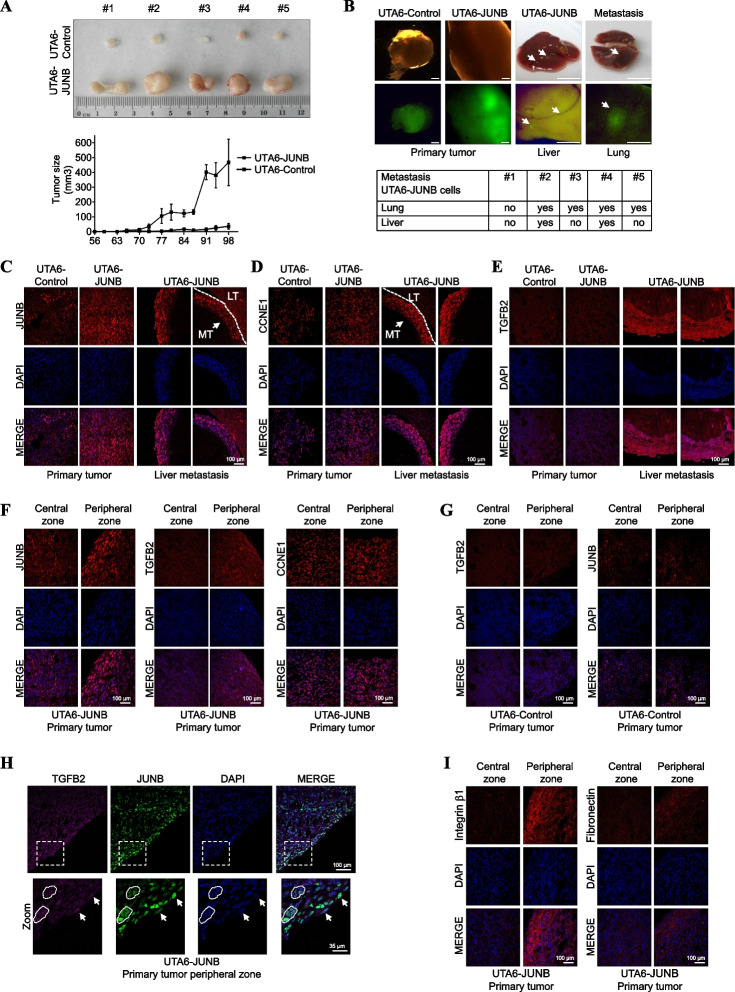
Overexpression of JUNB promotes tumor development and metastasis in vivo. **A ***JunB induces tumor growth in immunodeficient mice*. UTA6-JUNB and UTA6-control cells were subcutaneously injected in NSG mice. Tumor size in the subcutaneous xenograft model was measured twice a week (lower panel) and 3 months after implantation, mice were euthanized and dissected. Photographs of tumors retrieved at the end of the experiment (upper panel). **B ***JunB induces tumor metastasis in immunodeficient mice.* UTA6-control- and UTA6-JUNB-induced primary tumors and GFP expression in these tumors (Left panels, the scale bar is 1 mm), and representative image of liver and lung UTA6-JUNB-induced metastasis and GFP expression in these metastases (right panels, the scale bar is 1 cm). Arrows indicate metastatic lesions. Table reporting EGFP-expressing metastases in lung and liver of the 5 mice xenografted with UTA6-JUNB cells. **C–E ***Higher expression of JUNB, CCNE1, and TGFB2 in UTA6-JUNB liver metastases than in primary UTA6-JUNB tumors.* Representative immunofluorescence images of paraffin-embedded UTA6-JUNB and UTA6-Control primary tumor tissues, and UTA6-JUNB liver metastases. **C** Corresponds to JUNB, **D** corresponds to CCNE1, and **E** corresponds to TGFB2 analyses, respectively. DAPI staining (blue) indicates cell nuclei. Scale bars are indicated. **F ***JUNB and TGFB2 levels are increased in the border of UTA6-JUNB expressing primary tumors*. Representative immunofluorescence images of the central and peripheral zones of UTA6-JUNB. JUNB, TGFB, CCNE1 (red), and cell nuclei (blue) are observed. **G ***JUNB and TGFB2 levels are similar in the central and peripheral zones in control tumor tissues*. Representative immunofluorescence images of the central and peripheral zones of UTA6-Control tumor tissues. JUNB, TGFB (red), and cell nuclei (blue) are observed. **H ***JUNB and TGFB colocalization in the UTA6-JUNB primary tumor border.* Representative immunofluorescence images of UTA6-JUNB primary tumor tissue. TGFB2 (magenta), JUNB (green), and cell nuclei (blue) are observed. Arrows indicate cells co-expressing JUNB and TGFB2 proteins. **I ***Integrin β1 and Fibronectin levels are increased in the border of UTA6-JUNB expressing primary tumors*. Representative immunofluorescence images of UTA6-JUNB and control primary tumor tissues. Integrin β1 (red, left panel), fibronectin (red, right panel), and cell nuclei (blue) are shown. Scale bars are indicated. LT: Liver Tissue MT: Metastatic tissue

The fact that JUNB overexpression may confer a stronger EMT- and invasion-promoting potential in vitro to JUNB-overexpressing UTA6-JUNB cells as compared to UTA6-Control cells (Fig. 6 B–D) led us to ask whether high JUNB levels could also be associated with higher U2OS cell tumorigenicity in vivo. To this aim, UTA6-JUNB- and UTA6-Control cells were inoculated subcutaneously to immunocompromised NSG mice. Tumor size was then monitored at regular intervals and mice were eventually euthanized at 3 months post-inoculation. Although both cell lines were tumorigenic in all the animals used (5 per group), UTA6-JUNB cell-derived tumors were much bigger at the end of the experiments than UTA6-Control cell-derived ones (Fig. 7A, upper panel) and developed much faster after a shorter latency time (Fig. 7A, lower panel).

We also searched for lung and liver metastases in euthanized xenografted mice. This analysis was facilitated by the GFP-marking of xenografted cells, as the Tet-regulated plasmid used to generate them is a bicistronic vector expressing this fluorescent protein from an IRES sequence [13]. The feasibility of GFP-tracking of cancer cells was established by analyzing primary tumors (Fig. 7B, left upper panel). Whereas neither lung nor liver metastases were found in UTA6-Control cells, some were detected in both organs in 4/5 mice xenografted with UTA6-JUNB cells (Fig. 7B, upper right and lower panel). Hematoxylin and eosin staining (H&E) images of paraffin-embedded sectioned tissue slices from primary tumors and lung and liver metastasis are shown in Additional file 1: Fig. S7A. Specific tumor cells expressing GFP were also detected by immunohistochemistry (IHC) (Additional file 1: Fig. S7B-D) and immunofluorescence (IF) (Additional file 1: Fig. S8) assays. Importantly, IF analysis confirmed that both UTA6-JUNB primary tumors and liver and lung metastasis co-expressed GFP and JUNB protein (Additional file 1: Fig. S8). JUNB expression was observed at all tumor sites tested with signals stronger in UTA6-JUNB cell-derived primary tumors and metastases than in UTA6-Control cell-derived primary tumors analyzed by IF (Fig. 7C) and IHC (Additional file 1: Fig. S9A left panel, S9B and S9C). These data supported the idea that high JUNB expression confers a stronger tumorigenic potential to U2OS cells, leading to greater metastases.

Next, we examined CCNE1 expression by IF and IHC assays and observed that its expression was slightly higher in UTA6-JUNB cell-derived primary tumors than in UTA6-Control cell-derived primary tumors (Fig. 7D and Additional file 1: Fig. S9A left panel). Moreover, CCNE1 expression in UTA6-JUNB cell-derived liver metastases was higher in comparison to UTA6-JUNB cells in primary tumors (Fig. 7D and Additional file 1: Fig. S9B), suggesting that even higher expression of CCNE1 is also associated with metastasis. We also addressed TGFB2 expression in primary tumors and metastases by IF and IHC. No detectable differences in signal intensities could be detected between UTA6-Control and UTA6-JUNB cell-derived primary tumors when examinations were carried out inside tumors (Fig. 7E and Additional file 1: Fig. S9A left panel). However, stronger TGFB2 signals were recurrently found at the border of UTA6-JUNB cell-derived primary tumors (Fig. 7F middle panel and Additional file 1: Fig. S9A left panel), which was also where stronger JUNB signals were often observed (Fig. 7F, left panel, and 7H, and Additional file 1: Fig. S9A left panel). In contrast, CCNE1 protein levels remained similar in the border compared to the central zone (Fig. 7F, right panel, and Additional file 1: Fig. S9A left panel). Of note, no differences in JUNB and TGFB2 expressions were observed between the central and peripheral zones in the UTA6-Control primary tumors (Fig. 7G). We also observed strong fibronectin and integrin β-1 signals at the border UTA6-JUNB cell-derived primary tumors, suggesting that these cells have stronger migration and invasion capacities (Fig. 7 I and Additional file 1: Fig. S9A right panel). Accordingly, TGFB2 expression was stronger in UTA6-JUNB cell-derived liver metastases as compared to UTA6-JUNB cell-derived primary tumors (Fig. 7E, right panel, and S9B), suggesting that the cells with the highest metastatic capacity are also those showing the highest TGFB2 levels.

Thus, taken together, our data indicate that JUNB overexpression in U2OS cells promotes both tumor development and metastasis in vivo. They also suggest that the presence of TGFB2 in the tumor microenvironment may promote cell invasion and the metastatic capabilities of JUNB-overexpressing cells.

### High levels of JUNB protein associated with poorer outcomes in epithelial breast cancers

We next addressed whether high JUNB levels could be associated with bad prognosis in cancers by analyzing cancer data sets from the publicly accessible TCGA databank with cBioportal for Cancer genomics [53]. We found that *JUNB* copy number alterations (CNA) occur in several cancers including sarcoma, ovarian, esophagus, lung squamous carcinoma, and breast cancers (Additional file 1: Fig. S10A). Moreover, breast and ovarian cancer patients with high *JUNB* levels due to gene amplification showed poorer prognosis than the rest of patients (Additional file 1: Fig. S10B). As the publicly available data on protein expression in osteosarcoma are scarce, we addressed breast cancer, for which a wealth of data can be processed. In silico analysis of available published protein breast cancer dataset [54] using the Kaplan-Meier plots website [55] also showed that high levels of JunB protein were associated with bad prognosis (Additional file 1: Fig. S11A). In addition, JUNB protein levels correlated with cyclin E1 and TGFB2 protein levels (Additional file 1: Fig. S11B), suggesting that high JUNB protein levels may also promote aggressiveness of breast tumors by positively controlling their expression with consequences on both cell proliferation and invasion.

## Discussion

In the present study, we have shown that JUNB has important previously uncharacterized roles, not only in the control of cell proliferation, but also in cell invasion/metastatization by cancer cells with an epithelial phenotype. Moreover, we also report that JUNB impacts on these two processes depending on the extracellular environment.

### Control of the cell cycle by JUNB

In the first part of our investigations, we addressed cell proliferation regulation by JUNB under standard culture conditions and showed that JUNB promotes cell progression from G1 to S. Two lines of evidence supported this conclusion. First, depletion of JUNB in U2OS osteosarcoma cells, as well as in other epithelial cancer cells, was sufficient to reduce cell division by impairing the progression from G1 to S, leading to decreased entry into S and cell cycle arrest. Second, these data were corroborated by the observation that overexpression of JUNB accelerated the progression from G1 to S.

To understand how JUNB promoted cell cycle progression through G1, we pursued a comprehensive characterization of JUNB transcriptional activity in U2OS cells. Our data placed JUNB as a regulator of the expression of many genes involved in cell division control, which was consistent with our cell cycle analyses, and also suggested a role for JUNB in the regulation of EMT, angiogenesis, and responses to stresses via affecting E2F, KRAS, AKT, and TGFB signaling. The effects of RNAi-mediated downregulation of JUNB were both positive and negative in our transcriptomic study, which was expected since JUNB is known to activate or repress transcription depending on its target genes [10]. In most cases, the transcriptional alterations were modest, in keeping with the notion that JUNB is reputed to be a transcription factor showing weak transcription-regulating activity [56]. To help identify direct gene targets of JUNB, we also characterized the JUNB cistrome. A minority of JUNB binding sites (3%-range) were found in gene transcription promoters and the rest at more remote places with, however, the majority of these binding sites within less than 50 kb of the closest annotated gene TSSs. The crossing of our ChIP-seq data with publicly available data on histone modifications and chromatin accessibility in U2OS cells indicated that around 35% of JUNB binding sites lie within transcriptionally active enhancers (30%) and promoters (3%). Together, our data were coherent with the emerging notion that AP-1-binding sites usually do not reside within gene promoter regions but at distant regulatory elements, whatever the AP-1 dimers at play [1, 42]. Additionally, we observed that JUNB principally binds to TRE motifs, and not to CREs, in the vicinity of other transcription factor binding sites. AP-1 dimers formed by Jun and Fos proteins usually show higher affinity for TREs than for CREs whereas ATF protein-containing dimers preferentially recognize CREs [1], thus our data supported the idea that JUNB most likely little dimerizes with ATF family members in U2OS cells and most probably collaborates with other transcription factors to regulate the expression of its target genes.

Among several candidate JUNB target genes, we focused on two of them, *CCNE1* and *TGFB2,* for functional studies due to their known functions in cell cycle control and oncogenesis and because they were not previously known as JUNB targets. Thus, CCNE1 is a well-established driver of both the G1/S transition and DNA synthesis initiation [57]. Moreover, it has been reported critical for initiation of hepatocellular carcinoma [58] and is amplified and overexpressed in different types of cancer, especially in gynecologic malignancies such as ovarian and uterine serous carcinoma, where it leads to chromosome instability and, thereby, likely contributes to tumorigenesis [57, 59]. On its side, TGFB2 is a two-faceted cytokine well-known for its ability to, on the one hand, arrest the proliferation of normal cells or of early-stage cancer cells and, on the other hand, enhance tumorigenesis in a number of advanced cancers [47].

We showed that *CCNE1* is a positive target of JUNB in G1, consistently with its acknowledged positive role in G1/S transition. In addition, the cell cycle-dependent binding of JUNB to a TRE-containing enhancer region located just upstream the gene’s TSS also supported the idea that CCNE1 is a direct target of JUNB during G1 to S cell cycle progression. In the present stage of investigation, we cannot, however, exclude that other enhancers might collaborate with this one to regulate CCNE1 transcription.

Besides this, we identified the *TGFB2* gene as repressed by JUNB in G1, which we found consistent with, on one side, the positive role of JUNB we observed in this phase of the cell cycle and, on the other side, the described antiproliferative function of the TGFB2 cytokine. JUNB binding was found enriched in G1/S-synchronized cells at two TRE-containing sites located downstream of the TGFB2 TSS that were marked by the enhancer-specifying histone H3K4me1 modification. Accelerated G1/S transition triggered by JUNB overexpression correlated with both enhanced JUNB binding at the TGFB2 regulatory elements and lower expression of TGFB2. These data support the role of JUNB role as TGFB2 transcriptional repressor to facilitate proper G1/S cell cycle transition. It is however of note that one of these elements is marked by the active transcription-associated histone mark H3K27ac but not the other. This raises the two-fold possibility that these two putative regulatory elements may not have the same biochemical/biological functions and that TGFB2 gene transcriptional regulation may involve a functional balance between them and possibly other still-to-be-identified regulatory domains. Such an idea is supported by fine genome-wide analysis of the enhancers bound by another AP-1-constituting protein, FOSL1/FRA-1, as they show strong biochemical heterogeneity [42].

Thus, despite JUNB overexpression may affect AP-1 dimer composition and widely impact on gene expression and affect cell cycle progression, we report here that JUNB can positively control G1 to S phase progression. This regulation most likely involves a diversity of mechanisms. Our data reveal a to-date unknown positive action of JUNB on the *CCNE1* gene, which is consistent with the acknowledged role of *CCNE1* in this specific phase of the cell cycle. They also point to an important negative action of JUNB on the *TGFB2* gene, which would otherwise behave as a cell cycle brake.

### Regulation of EMT, invasion, and metastasis by JUNB

Advanced tumors are well-known to produce and secrete large amounts of TGFB and this is often associated with drastic cell signaling changes entailing loss of cell division repression by TGFB and promotion of tumor progression and metastasis by this cytokine [50, 51]. Our finding that JUNB is involved in the TGFB signaling and can repress the *TGFB2* gene consequently led us to wonder about the possibility that continuous and sustained cell stimulation by exogenous TGFB2 might overcome the effect of TGFB2 repression by JUNB. The results we present herein showed that this was actually the case.

In a first step, we addressed in vitro the proliferation of JUNB-overexpressing cells subjected to short-term or longer-term stimulation by TGFB2, the latter condition being intended to mimic continuous and sustained TGFB2 production by tumors. Under short-term stimulation, TGFB2 slowed down cell proliferation, consistently with its known antiproliferative effects. In contrast, under longer-term stimulation by TGFB2, we observed a JUNB-dependent signaling switch that, unexpectedly, enhanced endogenous TGFB2 protein production. Interestingly, this was not associated with a loss of JUNB ability to repress the TGFB2 gene but, rather, with a JUNB-dependent post-transcriptional mechanism entailing better translation of the TGFB2 mRNA. Future work will have to elucidate the intimate molecular mechanisms at play.

Phenotypic changes under long-term exposure of JUNB-overexpressing cells to TGFB2 were not limited to loss of cell proliferation inhibition by TGFB2. Thus, we also observed JUNB-dependent morphological and molecular changes specifying EMT. Moreover, these changes were also associated with a higher cell invasion capacity in in vitro assays and more tumorigenic and metastatic capacities when grafted to immunocompromised mice. Of note, higher levels of CCNE1 were found in JUNB-overexpressing primary tumors and in the metastatic lesions. This was coherent with our finding of CCNE1 being a gene positively regulated by JUNB and the higher in vivo proliferation capacity of these cells. Besides this, foci of higher TGFB2 expression could be observed at the borders of JUNB-overexpressing primary tumors but not in tumors derived from UTA6-Control cells. In addition, TGFB2 expression was found higher in metastatic lesions as compared to primary tumors generated by UTA6-JUNB cells. This provided further support to the notion of TGFB2 being key for the acquisition of stronger invasive ability by JUNB-overexpressing cells.

TGFB signaling has already received considerable attention as a therapeutic target in several cancers, leading to the development of TGFB inhibitors currently tested in clinical trials [60]. However, stronger prognosis markers are still desirable to identify the patients who might benefit the most [60]. In the view of our data, it is reasonable to hypothesize that tumors showing high JUNB activity might be particularly responsive to TGFB inhibition therapy. Finally, it is possible that other extracellular cues besides TGFB2 provided by the tumor environment might affect JUNB signaling in cancerous cells and, thereby, their tumorigenicity.

Finally, we conducted in silico analysis of clinical cancer data and show that amplification of JUNB associates with poor outcome in ovarian and breast cancer. Moreover, in silico meta-analysis of JUNB protein levels showed its association with poor survival in breast cancer patients and identified a positive correlation between JUNB, CCNE1, and TGFB2 expression levels in these tumors.

## Conclusions

Here we provide a genome-wide JUNB binding site study in proliferating U2OS cells showing that JUNB preferentially binds to transcriptionally active enhancers. By combining these data with transcriptomic data and functional studies, we demonstrate that JUNB facilitates cell proliferation, in part, via a positive action on CCNE1 and a negative action on endogenous TGFB2 expression. This cell division-promoting effect is however amplified under conditions of higher JUNB expression and it corresponds to those found in early-stage tumors abnormally overexpressing JUNB. Under conditions of sustained cancer cell stimulation by environmental TGFB2, such as those found in more-advanced stage tumors, JUNB overexpression not only supports cell proliferation, but also permits cells to gain a more tumorigenic phenotype via the acquisition of EMT, and invasion abilities, as well as tumor- and metastasis-forming properties. Consistently with our observations, the publicly available clinical data in breast and ovarian tumors strengthens the idea that JUNB overexpression can contribute to tumor aggressiveness. Altogether, our results on JUNB genomic, transcriptomic, and functional studies provide useful information that may be exploited for cancer prognosis and therapy.

## Materials and methods

### Cell culture, expression vectors, antibodies, and reagents

HeLa, U2OS, and U2OS-derived UTA6 cells were cultured in DMEM medium supplemented with 10% foetal bovine serum. H1395 and MCF7 cells were cultured in DMEM/F12 and RPMI respectively and supplemented with 10% foetal calf serum. Stable inducible UTA6 cell populations were generated following the protocol described in [11]. Cells were seeded at a density of 100,000–150,000 cells per well in a 6-well plate and grown to ~70 to ~80% confluency for all experiments. For M-G1/S cell synchronization, 1.5 × 10^6^ UTA6 cells were routinely seeded into 10-cm-diameter culture dishes in the presence of tetracycline. Twenty-four hours later, 2.5 mM thymidine was added for 24 h to induce a G1/S phase block. Cells were released in the cycle by washing out thymidine and subsequently cultured in standard culture medium without tetracycline and containing 0.04 μg/ml nocodazole for 16 h to induce a mitosis block. Mitotic cells were collected by shake-off, washed twice, and replated in nocodazole-free medium for subsequent culture. pCDNA3- and pTRE-JUNB expression vectors were previously described [11, 13]. Wild-type JUNB was cloned in the pEGFP-N1 vector (Clontech) to give pEGFP-JUNB plasmid. pCDNA3-cyclin E1 was a gift from Bob Weinberg (Addgene plasmid #8963; http://n2t.net/addgene:8963; RRID:Addgene_8963) [61]. Cells were transfected with 250 ng of cDNAs using Lipofectamine 2000 (Invitrogen). The antibodies used are listed in Additional file 7: Table S6. TGFB2 ligand (Pepro-Tech) was added to a final concentration of 8 ng/mL and medium with TGFB2 was replaced every day.

### Flow cytometry and EdU incorporation

Cells were washed once in ice-cold phosphate-buffered saline (PBS), fixed in 70% ethanol at −20°C for 2 h, resuspended in PBS solution containing 4 μg/ml of propidium iodide and 0.1 mg/ml of RNase A, and incubated overnight before quantification of propidium iodide fluorescence. For Edu incorporation assays, cells were treated with 2 μM of Click-iT EdU (5-ethynyl-2’-deoxyuridine) reagent (Click-iT™ Plus EdU Alexa Fluor™ 647, Invitrogen) for 1 h before harvesting and fixation in 70% ethanol at −20°C overnight. Cells were then incubated for 30 min with the Click-iT EdU reaction mixture, as indicated by the manufacturer, followed by another 30 min incubation with the propidium iodide and RNase A containing PBS solution. Fluorescence detection was performed using CytoFLEX flow cytometer (Beckman Coulter) and cell distribution in the cell cycle was determined with the FlowJo™ Software (Becton Dickinson) after gating out cell debris signals.

### Fluorescence microscopy

Cells seeded onto coverslips were washed with PBS and fixed with paraformaldehyde 4% for 20 min at room temperature. After three washes with PBS, they were permeabilized with 0.4% Triton X-100 in PBS for 10 min and incubated 1 h at 37°C with blocking buffer (PBS, 0.5% BSA, 0.05% Triton X-100). Cells were incubated overnight at 4 °C with anti-JUNB antibody in the blocking buffer, washed with PBS, and incubated with secondary antibody for 1 h. Actin filaments were labelled with Phalloidin-TRITC (Merck). Slides were mounted with ProLong™ Gold Antifade Mountant with DAPI (Thermo Fisher Scientific) and analyzed using a Leica confocal microscope TCS-SP2-AOBS (Leica Microsystems).

### Cell proliferation assay (MTS)

Cells were plated at a density of 2000 cells per well in 96-well plates, and cell proliferation was evaluated 72 h after with the CellTiter 96® Aqueous One Solution Cell Proliferation Assay (Promega, WI, USA). Cell titration assay was performed to optimize the initial cell number plated and to ensure that cell number and absorbance values obtained in the MTS assay are correlative according to the manufacturer’s instructions. Absorbance was measured with a Victor 2 plate reader (Perkin Elmer, MA, USA). When indicated, 0, 2, 4, 8, and 10 ng/mL of TGFΒ2 ligand were added to the media 24 h after seeding the cells and the medium with TGFB2 ligand was replaced every day.

### Cell invasion assay

Cell invasion assay was performed using transwell chambers (PET 8 μm, Sarstedt, Germany) pre-coated with Matrigel-GFR (Corning, NY, USA). Cells were seeded in serum-free DMEM and treated or not with TGFΒ2 ligand (8 ng/mL) for 24 h. Then, 5 × 10^4^ cells were inoculated into the upper chamber, and 750 μl of DMEM with 10% FBS was added to the lower chamber. After 24 h, the chambers were removed, and the uninvaded cells were wiped using a cotton-tipped swab. Then, filters were washed with PBS and stained with 0.5% crystal violet for 20 min at 25°C. Invaded cells were photographed and counted with an IN Cell Analyzer 2200 (GE Healthcare).

### Cell fractionation

UTA6-Control and UTA6-JUNB cells were seeded at low confluence and treated with TGFB2 ligand (8 ng/mL) for 48 h, refreshing it every 24 h. Cells were incubated with cycloheximide (CHX) (100 μg/ml, Calbiochem) for 5 min at 37°C, to block translation and sequester mRNA in polysomes, and trypsinized and washed with cold PBS supplemented with CHX. Cell pellet were then resuspended in cold sucrose buffer (0.25M Sucrose, 50mM Hepes, 60 mM KCl, 5mM MgCl_2_, 100 μg /mL CHX) supplemented with protease inhibitors and RNase inhibitor (20 U/ml). After 10 min incubation on ice, Triton X-100 was added at a final concentration of 0.2% and the cell lysate was mixed gently by inversion and incubated 5 more min on ice. Cell disruption with intact nuclei was analyzed by trypan blue and 5% of whole cell lysate was separated as input. The rest of cell lysate was centrifuged at 1000*g* and 16,000*g* for 5 and 15 min respectively at 4°C, and the last supernatant was centrifuged at 100,000*g* for 1 h at 4°C. The pellet was used as the polysome-enriched microsomal fraction for subsequent analysis.

### RT-qPCR

Total RNA was extracted using the High Pure RNA Isolation kit (Roche). Then, total RNA (1 μg) was used as a template to obtain the corresponding cDNA using a mixture of random hexamer primers and oligo (dT)_18_ primer and the PrimeScript RT reagent kit (Perfect Real Time, Takara Bio Inc., Otsu, Shiga, Japan) according to the supplier’s specifications. cDNAs were amplified using EvaGreen (CMB Bioline), and amplification products were detected by real-time PCR using the LightCycler 480 (Roche) according to the manufacturer’s specifications. RT-qPCR data were calculated by the 2^−ΔΔCt^ method by measuring the average cycle threshold (Ct) for the mRNA concerned and normalized to the values of the housekeeping gene S26, GUSB, or ACTB. The PCR primers used are listed in Additional file 8: Table S7.

### RNA interference experiments, transcriptome microarray assay, and data analysis

siRNAs (Additional file 9: Table S8) were transfected at a final concentration of 8 nM in UTA6, HeLa, H1395, and MCF7 cells using Interferine (Polyplus) transfection reagent according to the manufacturer’s instructions. For the transcriptome microarray assay, total RNA was extracted 48 h post-transfection using High Pure RNA Isolation kit (Roche). Triplicate RNA samples for each of the 4 conditions were quality checked (RIN>9) with Eukaryotic Total RNA Nano Kit on Bioanalyzer 2100 (Agilent) and then analyzed on GeneChip Human Transcriptome Array 2.0 (Affymetrix). Data were processed with Partek Suite and differentially expressed genes detected with the LIMMA package [62]. Significantly differentially expressed genes were selected based on a false discovery rate (FDR) threshold of less than 0.05.

### ChIP-seq and data analysis

ChIP experiments were performed essentially as described in [63]. Briefly, 5 × 10^7^ UTA6 cells growing asynchronously were crosslinked with 1% formaldehyde for 10 min at room temperature followed by formaldehyde quenching with 125 mM glycine. Chromatin was fragmented by sonication for 10 min (30 s on/off) at maximum power using the Bioruptor system from Diagenode and then incubated overnight at 4°C with 10 μl of anti-JUNB antibody (Cell Signaling (#3753)) previously bound to Dynabeads Protein G (Thermo Fisher Scientific). The immunoprecipitate was washed, de-crosslinked, and digested with proteinase K and RNase A as indicated in [63]. DNA was purified using Nucleospin Gel and PCR cleanup (Macherey-Nagel) according to the manufacturer’s recommendations. For ChIP-seq, two biological replicates were prepared. For each sample, an unbound fraction (input) was used for normalization in all subsequent bioinformatics analyses.

DNA concentration was measured by Qubit (Thermo Fisher Scientific) and sequencing DNA libraries were prepared from 8 to 10 ng of DNA using TruSeq ChIP Sample Prep Kit (Illumina). DNA quality was assessed with the High Sensitivity DNA Analysis Kit on Bioanalyzer 2100 (Agilent). Sequencing was performed on Illumina HiSeq 2000 system. Image analyses and base calling were performed using the HiSeq Control Software (HCS) and Real-Time Analysis component (RTA). Reads were aligned to the human genome (hg19) using CASAVA (Illumina). Peak calling was performed using MACS2 [64] and *p*-value cutoff 1.00e−05, setting as control the input samples. Only peaks found across biological replicates (BEDtools [65], 1 bp minimum overlapping) were considered as binding sites for downstream analysis. ChIP-seq data were visualized using the Integrative Genomics Viewer (IGV) [66] and the UCSC Genome Browser (hg 19) ([67]; http://genome.ucsc.edu/).

Association of binding sites to putative target genes was obtained with RGmatch [68] using default distance parameters and RefSeq GTF (Release 105) as gene model reference.

For association of histone marks to JUNB binding peaks, ChIP-seq data of H3K4me3, H3K4me1, and H3K27ac [43] with approximately 4–7M reads were mapped using the hg19 reference genome and the bowtie2 algorithm [69]. The number of readings around the center of the JUNB consensus peaks were counted using BEDtools [65]. For each peak, 60 bins of 100bp were defined, around the center of the peak, thus covering a total of 6 kb. The results were scaled and visualized in R using heatmap3 package.

Motif analyses were performed with MEME suite [46]. MEME-Chip [70] was used to capture enrichment of motifs at binding regions. FIMO tool [70] was used to scan AP-1/TRE and AP-1/CRE motifs within the set of defined ChIP regions. Position weight matrices for CRE/TRE motifs were obtained from the JASPAR database [71].

### Gene Set Enrichment Analysis

Gene Set Enrichment Analysis (GSEA, Broad Institute) was performed using the tool available at http://www.broadinstitute.org/gsea/index.jsp [33]. Briefly, fold change (log2) in gene expression from two experimental conditions was calculated and the list was then used as a ranked list in the Pre-Ranked function of GSEA software. Gene ontology analysis were performed with AmiGO [72], Panther [73], GSEA [33], ENCODE [74] and db.EMT [75].

### Xenograft mouse models

Ten NSG mice (NOD.Cg-Prkdcscid Il2rgtm1WjI/SzJ, Charles River) aged 5–6 weeks were randomly divided into two groups of 5 for subcutaneous tumor cell injection of UTA6-control-IRES-EGFP and UTA6-JUNB-IRES-EGFP-established cell lines. Single-cell suspensions were prepared at a concentration of 1×10^7^ cells/ml in 100 μl of serum-free medium and Matrigel (1: 1) and injected subcutaneously (100 μl/mouse) in the dorsal flank region of mice (*n*=5/cell line). Three days post-injection mice behavior, weight, and tumor growth were analyzed. Tumor size was measured with a caliper twice a week and tumor volume (V) was obtained using the formula: *V* (mm^3^) = *d*^2^ × *D*/2, where *V* is tumor volume, *d* is the shorter diameter, and *D* is the longest diameter. Three months after implantation, mice were euthanized and the tumors, lungs, and livers were isolated and fixed in 4% paraformaldehyde for histological and morphological study. Metastatic GFP expressing cells were tracked in the whole organs by fluorescence microscopy using a Leica MZ 16 FA microscope.

For immunohistochemistry analysis, 7-μm-thick tissue sections were washed with PBS and antigen unmasking was performed using a 10 mM citrate buffer adjusted at pH 6. The sections were blocked using blocking buffer (10% Newborn Goat Serum and 0.1% Triton in PBS), incubated overnight with primary antibodies O/N at 4°C in blocking solution, washed with 0.05% Triton diluted in PBS, and incubated with Alexa Fluor secondary antibodies at room temperature for 2 h. The sections were mounted using the ProLong Gold Antifade with DAPI (Invitrogen).

### Analysis of cancer clinical datasets

Frequencies of JUNB gene alterations (mutation, amplification, and deletion) in several cancer datasets (TCGA Pan-Cancer Atlas Studies) and *Z*-score of JUNB amplification alteration in breast invasive carcinoma (TCGA, Firehose Legacy, 1101 patients), Molecular Taxonomy of Breast Cancer International Consortium (METABRIC, 1979 patients), and Ovarian cancer (TCGA, Pan-Cancer Atlas Studies, 557 patients) were obtained from cBioPortal for Cancer Genomics [53] in June 2021. Meta-analysis of breast cancer protein datasets was performed using a Kaplan-Meyer plotter (http://kmplot.com) [76] with default settings. Breast Invasive Carcinoma (TCGA, Firehose Legacy) (1101 patients) was downloaded from cBioPortal. *Z*-scored expression values of protein were obtained in June 2021.

### Statistics analysis

For statistical analysis, ANOVA and *t*-test analyses were performed using the GraphPad Prism software version 7.00 for Windows.

## Supplementary Information

Additional file 1: Figure S1. Depletion of JUNB inhibits cell proliferation in epithelial cancer cells. Figure S2. Validation by RT-qPCR of a panel of JUNB targets genes identified in the transcriptome approach and involved in cell cycle regulation and cell proliferation. Figure S3. JUNB binding at the TGFB2, RBPJ, ERCC2 and RPTOR locus. Figure S4. JUNB binds to and regulates the expression of TGFB2 gene. Figure S5. JUNB binds to and regulates the expression of CCNE1 gene. Figure S6. Overexpression of JUNB promotes TGFB2 signaling-induced EMT. Figure S7. H&E images and IHC images of GFP expression in UTA6-Control, UTA6-JUNB primary tumors and metastatic lung and liver lesions. Figure S8. JUNB and GFP expression in UTA6-UTA6-JUNB primary tumors and metastatic lung and liver lesions. Figure S9. IHC images of JUNB, TGFB2, CCNE1, Fibronectin and Integrin β1 in UTA6-Control and UTA6-JUNB primary tumors, and of JUNB, TGFB2 and CCNE1 in metastatic lung and liver lesions. Figure S10. JUNB amplification in breast and ovarian cancers is associated with poor survival. Figure S11. Expression of JUNB protein in breast cancer is associated with poor survival. Supplemental methods for cell proliferation by living cell imaging. Figure S12. Uncropped western blot gel images in Fig. 1B. The dotted line boxes highlight lanes used in figures. Figure S13. Uncropped western blot gel images in Fig. 2D. The dotted line boxes highlight lanes used in figures. Figure S14. Uncropped western blot gel images in Fig. 4E. The dotted line boxes highlight lanes used in figures. Figure S15. Uncropped western blot gel images in Fig. 5 A-B. The dotted line boxes highlight lanes used in figures. Figure S16. Uncropped western blot gel images in Fig. 6 B and F. The dotted line boxes highlight lanes used in figures. Figure S17. Uncropped western blot gel images in Fig. S1A and S1E. The dotted line boxes highlight lanes used in figures. Figure S18. Uncropped western blot gel images in Fig. S2E. The dotted line boxes highlight lanes used in figures. Figure S19. Uncropped western blot gel images in Figure S4C and S4D. The dotted line boxes highlight lanes used in figures. Figure S20. Uncropped western blot gel images in Figure S5A. The dotted line boxes highlight lanes used in figures. Figure S21. Uncropped western blot gel images in Figure S6A and S6C. The dotted line boxes highlight lanes used in figures.

Additional file 2: Table S1. Regulated genes in siJUNB-transfected cells compared to siControl-transfected cells.

Additional file 3: Table S2. High-confidence JUNB regulated genes involved in cell cycle regulation and E2F pathway.

Additional file 4: Table S3. High-confidence JUNB regulated genes involved in EMT and TGFB signalling.

Additional file 5: Table S4. Genes assigned to JunB binding sites. Each peak was assigned to the nearest TSS.

Additional file 6: Table S5. High-confidence JUNB regulated genes containing JUNB binding sites of ChIP-seq analysis.

Additional file 7: Table S6. List of antibodies used for western blot, immunofluorescence and immunohistochemistry.

Additional file 8: Table S7. List of primers used for real-time PCR.

Additional file 9: Table S8. List of siRNA.

Additional file 10. Review history.

## Data Availability

JunB transcriptome and ChIP-seq data generated in this study have been deposited in the Gene Expression Omnibus (GEO) under the accession number GSE195993 [77]. The two linked subseries on transcriptome and ChIP-seq can be found respectively under the accession numbers GSE195987 and GSE195991. For association of histone marks to JUNB binding peaks, sequencing data available on the GEO database under the accession numbers GSM1356566, GSM1356565, and GSM1356567 were used [43]. For ATAC-seq analysis, sequencing data available on the GEO database under the accession number GSM4133297 was used [45]. The data supporting the conclusions reported in this study are available within the article and its supplementary materials which will be available in the public database PubMed. The authors also agree to provide the data upon request.
