# Supplementary material for: New roles for AP-1/JUNB in cell cycle control and tumorigenic cell invasion via regulation of cyclin E1 and TGF-β2
Source: Genome Biol. 2022 Dec 9;23:252. doi: 10.1186/s13059-022-02800-0 (PMC9733061; doi:10.1186/s13059-022-02800-0)
Supplement: Supplementary file 1 — Additional file 1: Figure S1. Depletion of JUNB inhibits cell proliferation in epithelial cancer cells. Figure S2. Validation by RT-qPCR of a panel of JUNB targets genes identified in the transcriptome approach and involved in cell cycle regulation and cell proliferation. Figure S3. JUNB binding at the TGFB2, RBPJ, ERCC2 and RPTOR locus. Figure S4. JUNB binds to and regulates the expression of TGFB2 gene. Figure S5. JUNB binds to and regulates the expression of CCNE1 gene. Figure S6. Overexpression of JUNB promotes TGFB2 signaling-induced EMT. Figure S7. H&E images and IHC images of GFP expression in UTA6-Control, UTA6-JUNB primary tumors and metastatic lung and liver lesions. Figure S8. JUNB and GFP expression in UTA6-UTA6-JUNB primary tumors and metastatic lung and liver lesions. Figure S9. IHC images of JUNB, TGFB2, CCNE1, Fibronectin and Integrin β1 in UTA6-Control and UTA6-JUNB primary tumors, and of JUNB, TGFB2 and CCNE1 in metastatic lung and liver lesions. Figure S10. JUNB amplification in breast and ovarian cancers is associated with poor survival. Figure S11. Expression of JUNB protein in breast cancer is associated with poor survival. Supplemental methods for cell proliferation by living cell imaging. Figure S12. Uncropped western blot gel images in Fig. 1B. The dotted line boxes highlight lanes used in figures. Figure S13. Uncropped western blot gel images in Fig. 2D. The dotted line boxes highlight lanes used in figures. Figure S14. Uncropped western blot gel images in Fig. 4E. The dotted line boxes highlight lanes used in figures. Figure S15. Uncropped western blot gel images in Fig. 5 A-B. The dotted line boxes highlight lanes used in figures. Figure S16. Uncropped western blot gel images in Fig. 6 B and F. The dotted line boxes highlight lanes used in figures. Figure S17. Uncropped western blot gel images in Fig. S1A and S1E. The dotted line boxes highlight lanes used in figures. Figure S18. Uncropped western blot gel images in Fig. S2E. The dotted l [file 13059_2022_2800_MOESM1_ESM.docx]

Supplementary Materials for

- New roles for AP-1/JUNB in cell cycle control and tumorigenic cell invasion via regulation of cyclin E1 and TGF-β2

Beatriz Pérez-Benavente, Alihamze Fathinajafabadi, Lorena de la Fuente, Carolina Gandía, Arantxa Martínez-Férriz,José Miguel Pardo-Sánchez, Lara Milián, Ana Conesa, Octavio Romero, Julian Carretero, Rune Matthiesen, Isabelle Jariel-Encontre, Marc Piechaczyk, and Rosa Farràs.

.

Correspondence to: rfarras@cipf.es

**This PDF file includes:**

Additional file 1: Figs. S1 to S21 and Supplemental methods

**
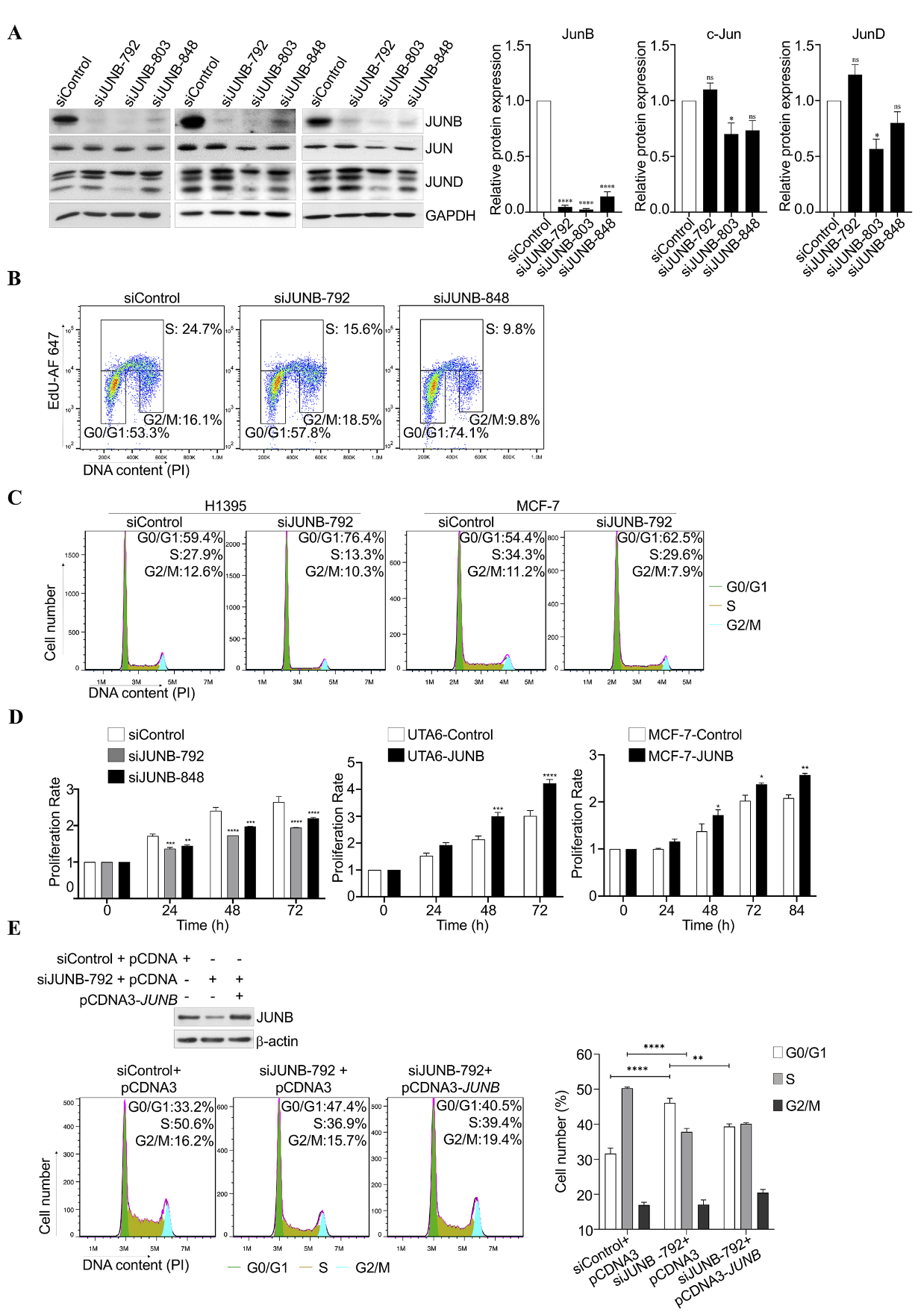
**

**Fig. S1.** **Depletion of JUNB inhibits cell proliferation in epithelial cancer cells.** (**A**) Quantification of *Jun family protein levels upon transfection of JUNB siRNAs* of three independent experiments processed as in Figure 1B. JUNB, JUN and JUND protein levels were estimated from the intensity of the signals on western blots using the ImageJ software and calculated as a ratio of each protein band relative to GAPDH. Statistical analyses were performed using one-way ANOVA test (*p < 0.05, ****p < 0.0001, ^ns^ not significant). (**B**) DNA content was analyzed by two-parametric flow cytometry after propidium iodide staining together with EdU incorporation in HeLa cells transfected with siControl, siJUNB-792 or siJUNB-848 for 72 h. Fluorescence intensity of cells stained with the Alexa Fluor 647 azide (AF-647) and PI is shown. (**C**) DNA content of H1395 lung cancer cells and MCF-7 breast cancer cells transfected with siControl and siJUNB-792 for 72 h were analysed by flow cytometry of propidium iodide (PI)-stained cells. (**D**) Proliferation rate was quantified by measuring cell confluence values of phase contrast images acquired by time-lapse microscopy. (Left panel) U2OS cells transfected with siRNA against JUNB. Images were acquired at the time of transfection (time 0 h) and for the indicated times. (Middle panel) U2OS-UTA6 cells expressing either an inducible empty pTRE vector (UTA6-Control) or an inducible JUNB encoding pTRE vector (UTA6-JUNB) [11, 13] . (Right panel) MCF-7 cells stably expressing either a pEGFP vector (MCF-7-Control) or JUNB-GFP encoding pEGFP vector (MCF-7-JUNB). Images were acquired 24 h after seeding the cells (time 0 h) and for the indicated times. (**E**) Ectopic expression of JUNB in siJUNB-transfected U2OS cells rescues cell cycle arrest.Upper panel: U2OS cells were transfected with siJUNB-792 for 72 h plus or minus *pCDNA3-JUNB* for 48 h. Protein extracts of the transfected cells were analysed by immunoblot using anti- JUNB antibody. β-actin was used as a loading control. Lower left panel: typical flow cytometry histogram of the cell cycle distribution of cells transfected with siControl or siJUNB-792 and empty pCDNA3 or pCDNA3 encoding JUNB as indicated on the figure. Lower right panel: quantification of 3 independent flow cytometry experiments conducted with siJUNB-792. Statistical analyses were performed using Two-way ANOVA with Tukey´s multiple comparisons test and one-tailed paired t-test (**p < 0.01, ****p < 0.0001).


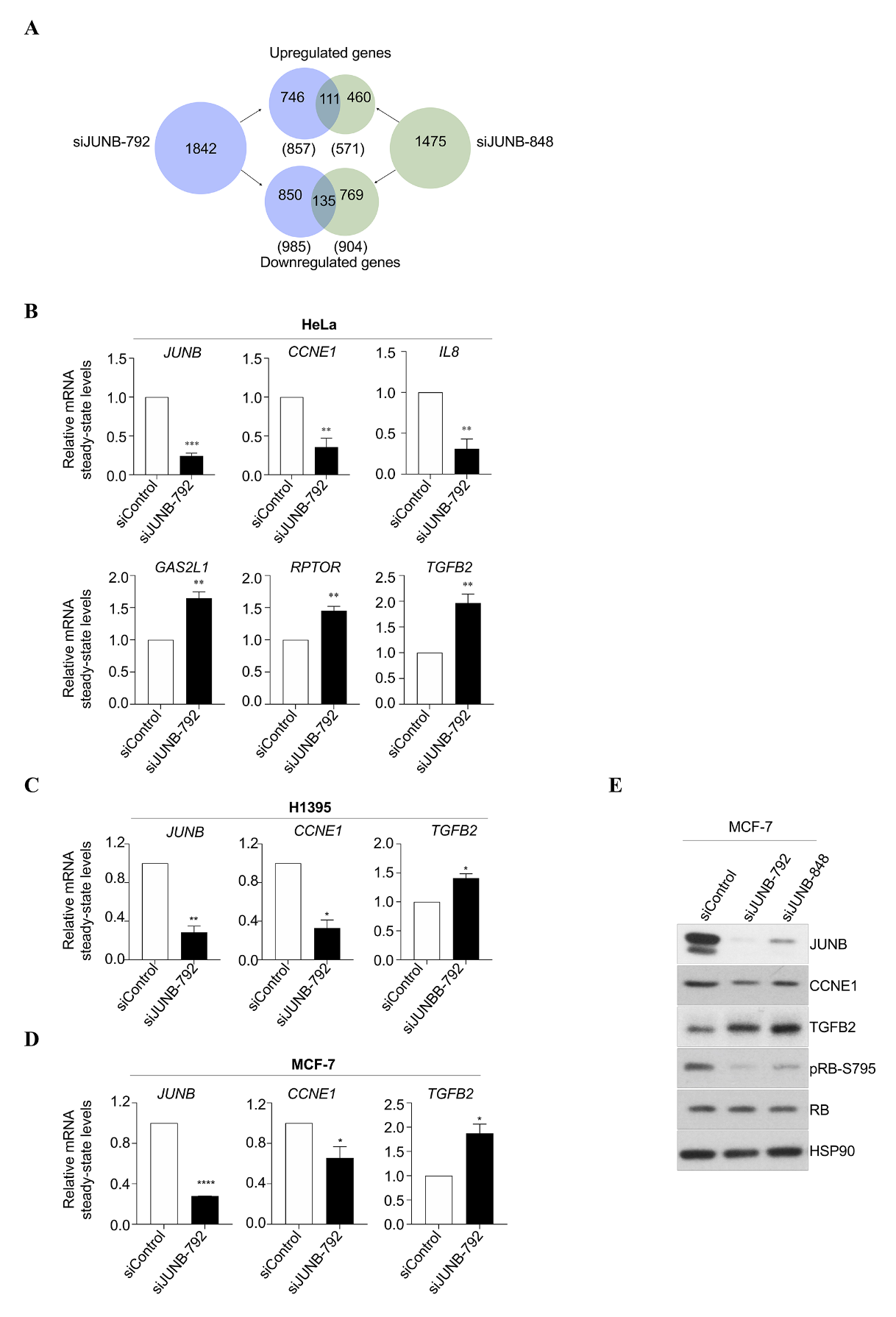


**Fig. S2.** **Validation by RT-qPCR of a panel of JUNB targets genes identified in the transcriptome approach and involved in cell cycle regulation and cell proliferation***.* (**A**) Proportional Venn diagram representing the intersection of genes upregulated or downregulated in our transcriptomic study using 3 biological replicates for siJUNB-792 (blue) and siJUNB-848 (green) transfected U2OS cell line (FDR < 0.05). (**B**) RT-qPCR validation of a panel of JUNB-regulated genes in HeLa cells 48h after transfection with either siControl, siJUNB-792 or siJUNB-792. Data shown are the means with SEM from three independent experiments. Statistical analyses were performed by one-tailed paired t-test (**p<0.01, ***p<0.001). (**C** and **D**) RT-qPCR validation of a panel of JUNB target genes in H1395 and MCF7 cells 48h after transfection with either siControl, or siJUNB-792. Results are the means with SEM of three independent experiments. Statistical analyses were performed using one-tailed paired t-test (*p<0.05, **p<0.01, ****p<0.0001). (**E**) Depletion of JUNB leads to a decrease in pRB and CCNE1 protein levels, and to an increase in that of TGFB2 in MCF-7 cells. Cells were transfected with siControl, siJunB-792 or siJunB-848 for 72 h and protein extracts were analysed by immunoblotting using the indicated antibodies.


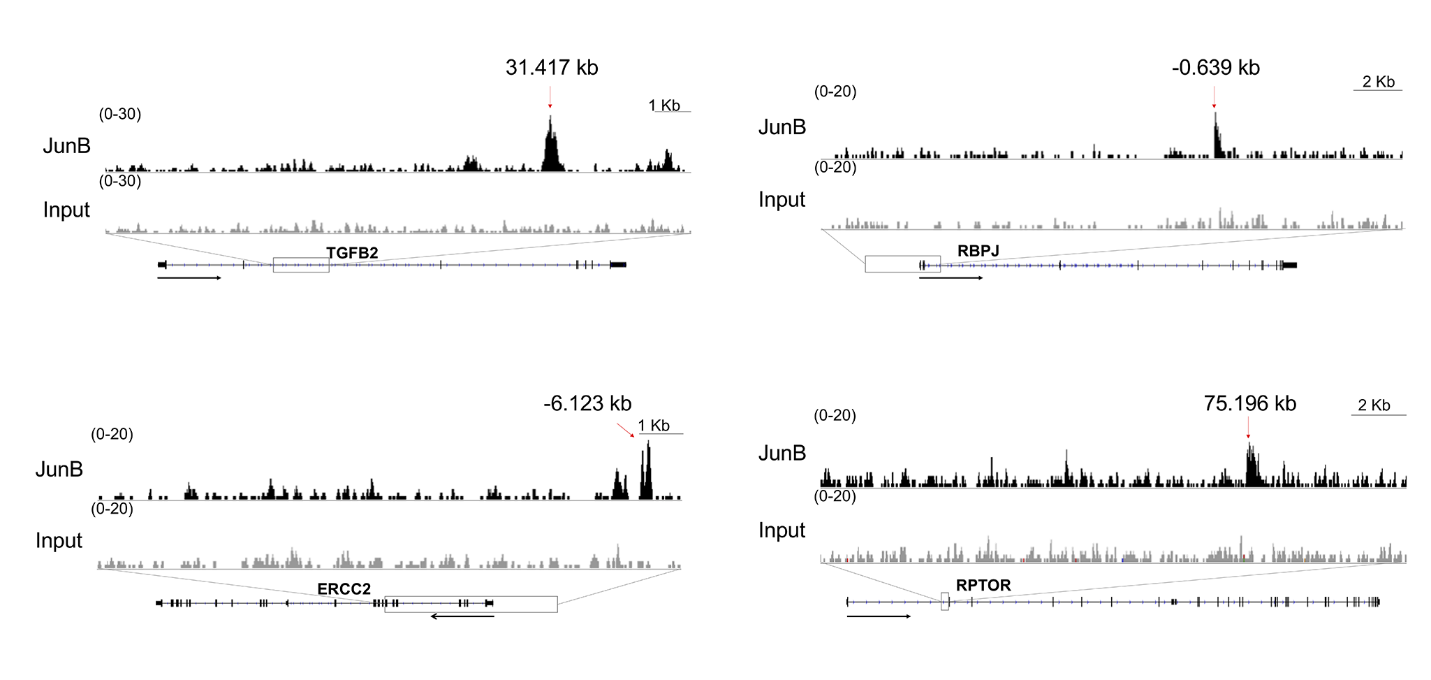


**Fig. S3**. **JUNB binding at the TGFB2, RBPJ, ERCC2 and RPTOR locus.** The *indicated loci* are represented using the Integrative Genomics Viewer (IGV). The bottom tracks show UTRs, coding regions and introns indicated by thin- or thick boxes and lines, respectively of the indicated genes. The direction of transcription is from left to right. The positions of the JUNB peaks relative to the TSS are indicated. The ChIP replicate with 15678 peaks was used.


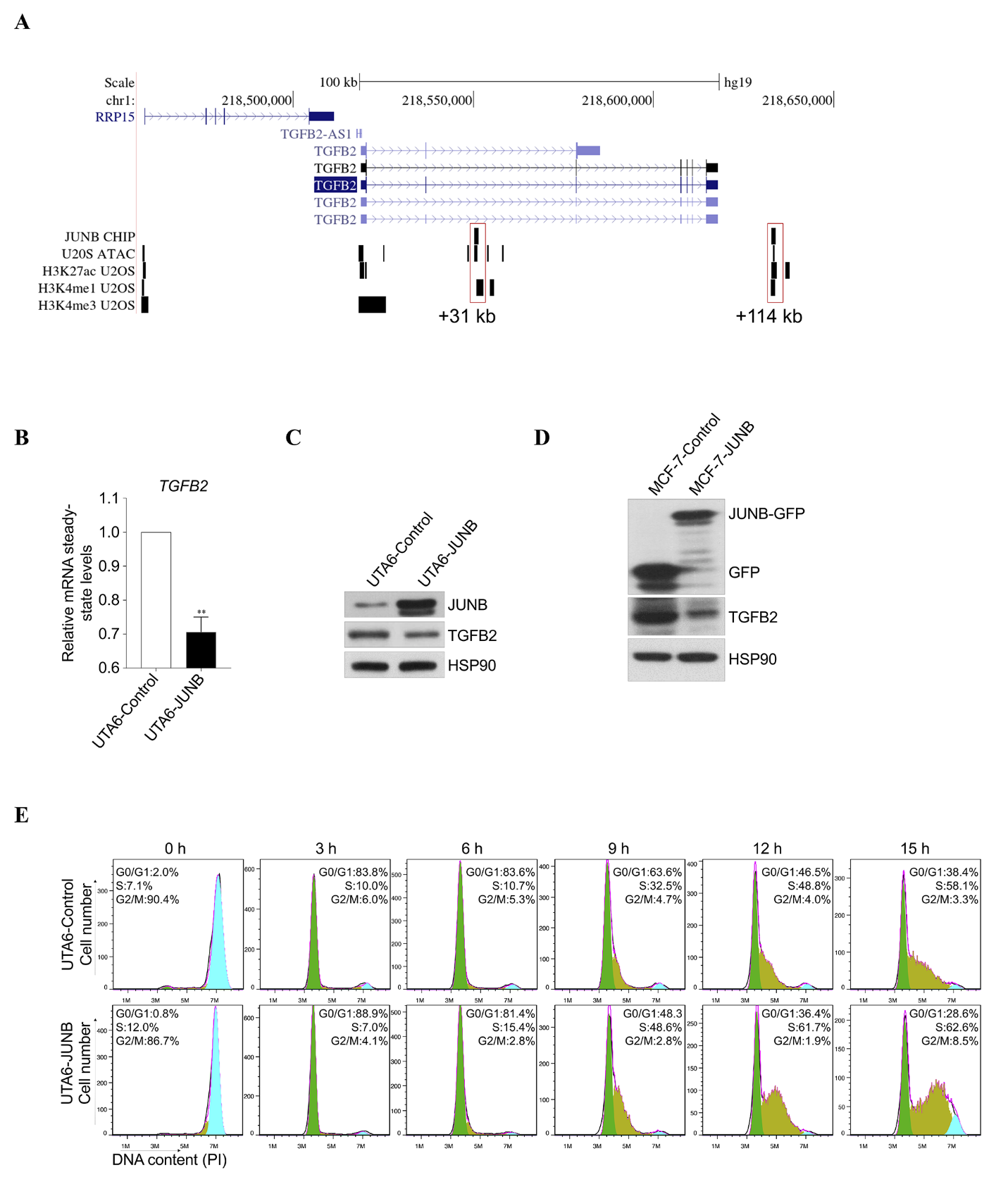


**Fig. S4. JUNB binds to and regulates the expression of TGFB2 gene***.* (**A**) *JUNB binding at the TGFB2 locus are associated with H3K4me1 and H3K27ac in U2OS cells. The* JUNB binding site at 31 kb downstream of the *TGFB2* TSS is surrounded by H3K4me1, and located at 114 kb is surrounded by H3Kme1 and H3K27ac in U2OS cells. UCSC genome browser snapshot of JUNB binding to *TGFB2* region at 31 and 114 kb downstream of TSS on human genome (hg19) chromosome 1 (chr1). The *TGFB2* locus is represented using the UCSC software. JUNB ChIP lane corresponds to our ChIP-seq data in U2OS cells. The rectangle in red indicates the JUNB peak identified at 31 and 114 kb downstream of the TGFB2 TSS. U2OS H3K27ac, H3K4me1 and H3K4me3 lanes correspond to ChIP-seq data in U2OS cells published in [42]. (**B**) *JUNB represses TGFB2 transcription in exponentially growing cells*. *TGFB2* mRNA level in UTA6 cells expressing either an inducible empty pTREvector (UTA6-Control) or an inducible JUNB encoding pTRE vector (UTA6-JUNB) grown in the absence of tetracycline were analysed by RT-qPCR. Data are shown as means with SEM from three independent experiments. Statistical analyses were performed using the one-tailed paired t-test (**p<0.01). (**C**-**D**) *JUNB overexpression decreases TGFB2 protein levels in exponentially growing cells*. (**C**) Protein extracts of UTA6-Control and UTA6-JUNB cells and (D) established MCF-7 cells expressing GFP (MCF-7-Control) or JUNB-GFP (MCF-7-JUNB) were analysed by immunoblotting using anti-JUNB, -GFP and -TGFB2 antibodies. HSP90 was used as a loading control. (**E**) *UTA6-Control or UTA6-JUNB were synchronized in mitosis by using a double block with thymidine and nocodazole.* Histograms showing the distribution of cells in the cell cycle of synchronized inducible UTA6-Control and UTA6-JUNB of experiment shown in Figure 4C. Cells were analysed by flow cytometry after staining of DNA with propidium iodide.


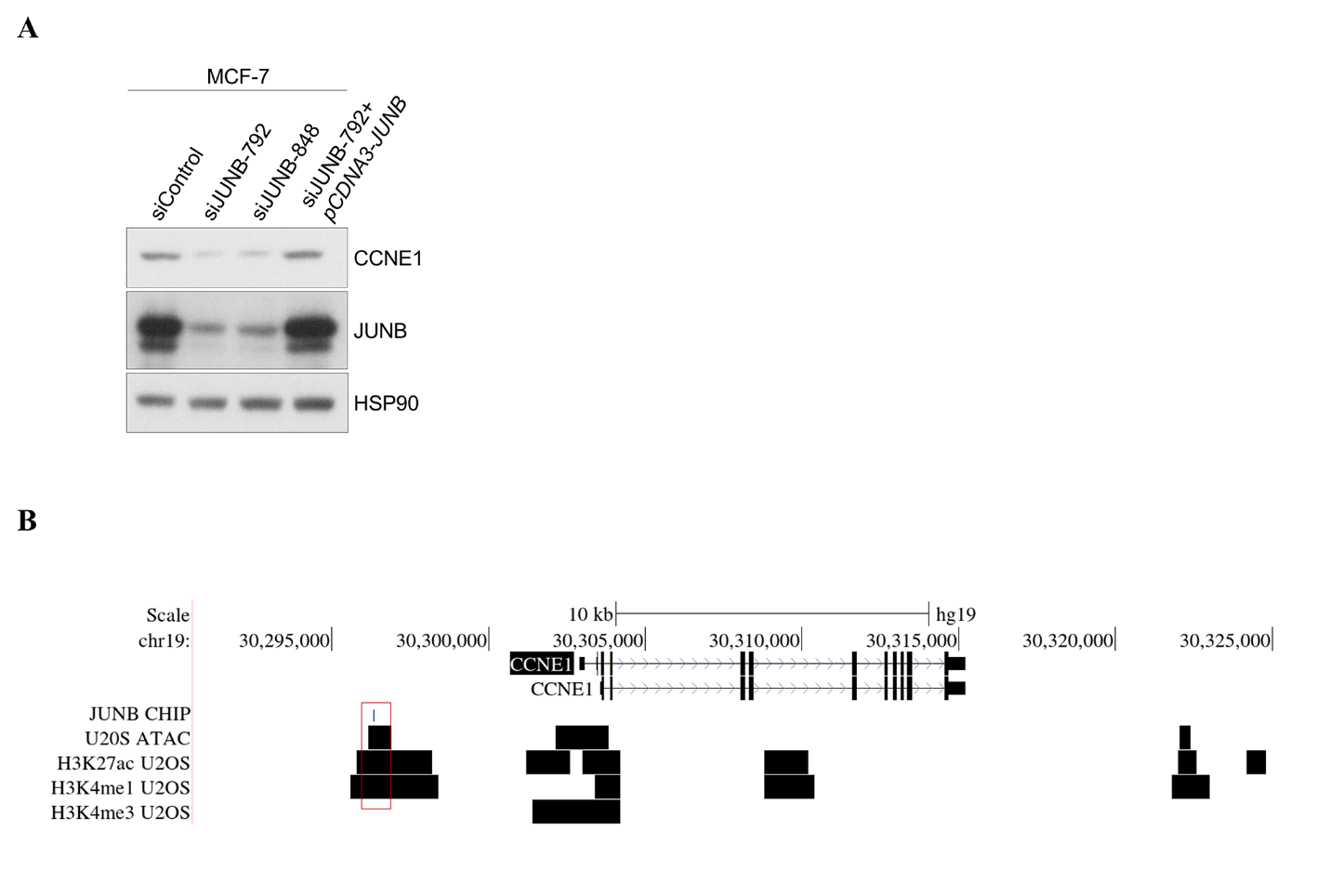


**Fig. S5. JUNB binds to and regulates the expression of CCNE1 gene.**** (A)** *Ectopic expression of JUNB in siJUNB-transfected MCF-7 cells restored endogenous CCNE1 protein expression in MCF-7 cells.* Cells were transfected with siJUNB-792 and siJUNB-848 for 72 h plus or minus *pCDNA3-JUNB* for 48 h. Protein extracts of the transfected cells were analysed by immunoblot using anti-JUNB and CCNE1 antibodies. HSP90 was used as a loading control. **(B)** *JUNB binding sites at the CCNE1 locus are associated with H3K4me1 and H3K27ac in U2OS*. JUNB binding at 6.1 kb upstream of the *CCNE1* TSS is associated with H3K4me1 and H3K27ac in U2OS. UCSC genome browser snapshot of JUNB binding to a *CCNE1* region at 6.1 kb upstream of TSS on human genome (hg19) chromosome 19 (chr19). The *CCNE1* locus is represented using the UCSC software. The JUNB ChIP lane corresponds to our ChIP-seq data in U2OS cells. U2OS H3K27ac, H3K4me1 and H3K4me3 lanes correspond to ChIP-seq data in U2OS cells published in [42]. The rectangle in red indicates the JUNB peak identified at 6.1 kb upstream of the CCNE1 TSS.


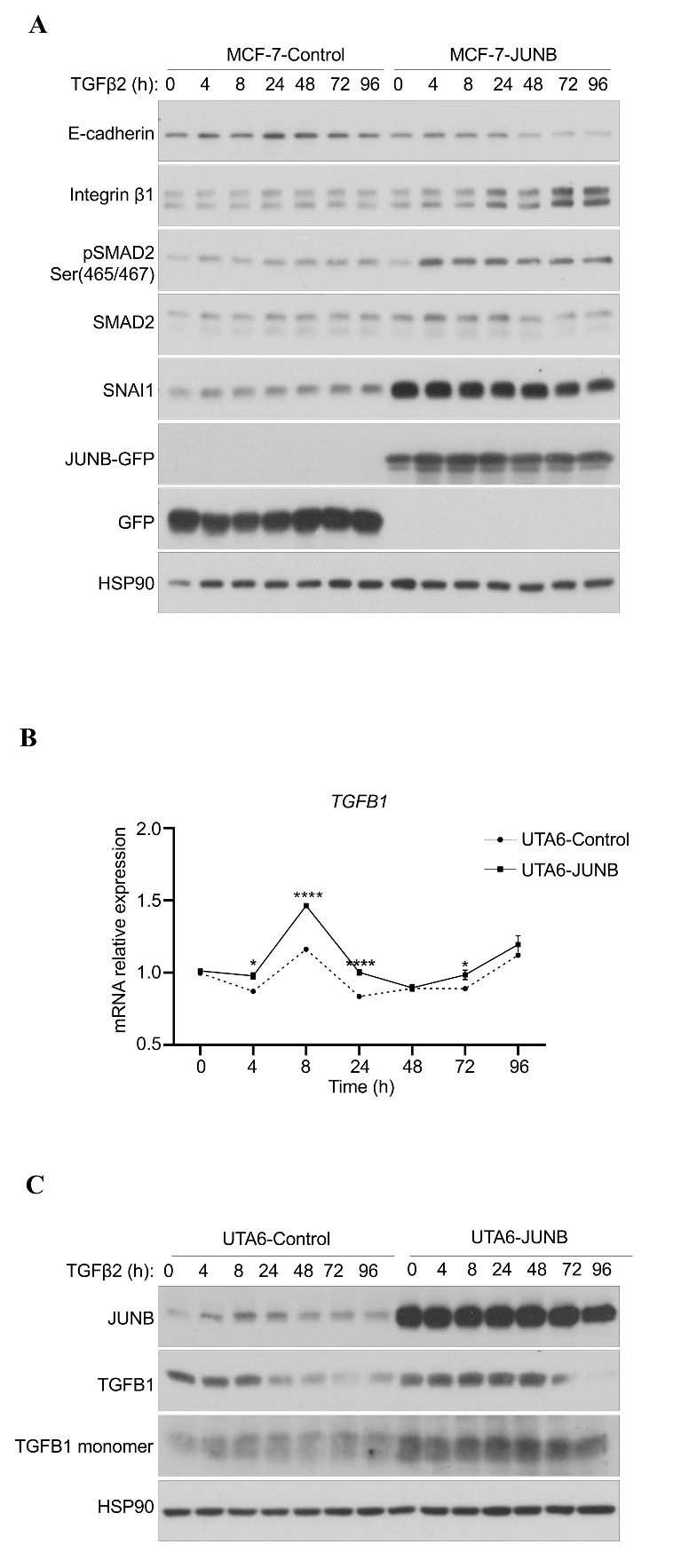


**Fig. S6**. **Overexpression of JUNB promotes TGFB2 signalling-induced EMT.** (**A**) *Overexpression of JUNB promotes the expression of mesenchymal proteins in MCF-7 cells exposed to TGFB2 ligand*. (A) Immunoblots show the levels of JUNB, E-cadherin and mesenchymal markers (SMAD2, pSMAD2 (Ser465/467), SNAI1, and Integrin β1) in cells stably expressing JUNB-GFP (MCF-7-JUNB) or GFP (MCF-7-Control) expression vectors treated with 8 ng/ml of TGFβ2 ligand for the indicated times. HSP90 is used as a loading control. (**B**)  *TGFB1 mRNA levels in UTA6 cells exposed to TGFB2 ligand*. Relative *TGFB1* mRNA levels in UTA6-Control and UTA6-JUNB cells treated with 8 ng/ml of TGFB2 ligand for the indicated times were analyzed by RT-qPCR. Data are shown as means with SEM from 3 independent experiments. Statistical analyses were performed using two-way ANOVA with Tukey´s multiple comparisons test. (*p<0.05, ***p<0.001). (**C**) Protein abundance of JUNB, TGFB1 and TGFB1 monomer in asynchronously growing UTA6-Control and UTA6-JUNB cells in the absence of tetracycline treated with 8 ng/ml of exogenous TGFB2 ligand were analysed by immunoblotting. HSP90 was used as a loading control.


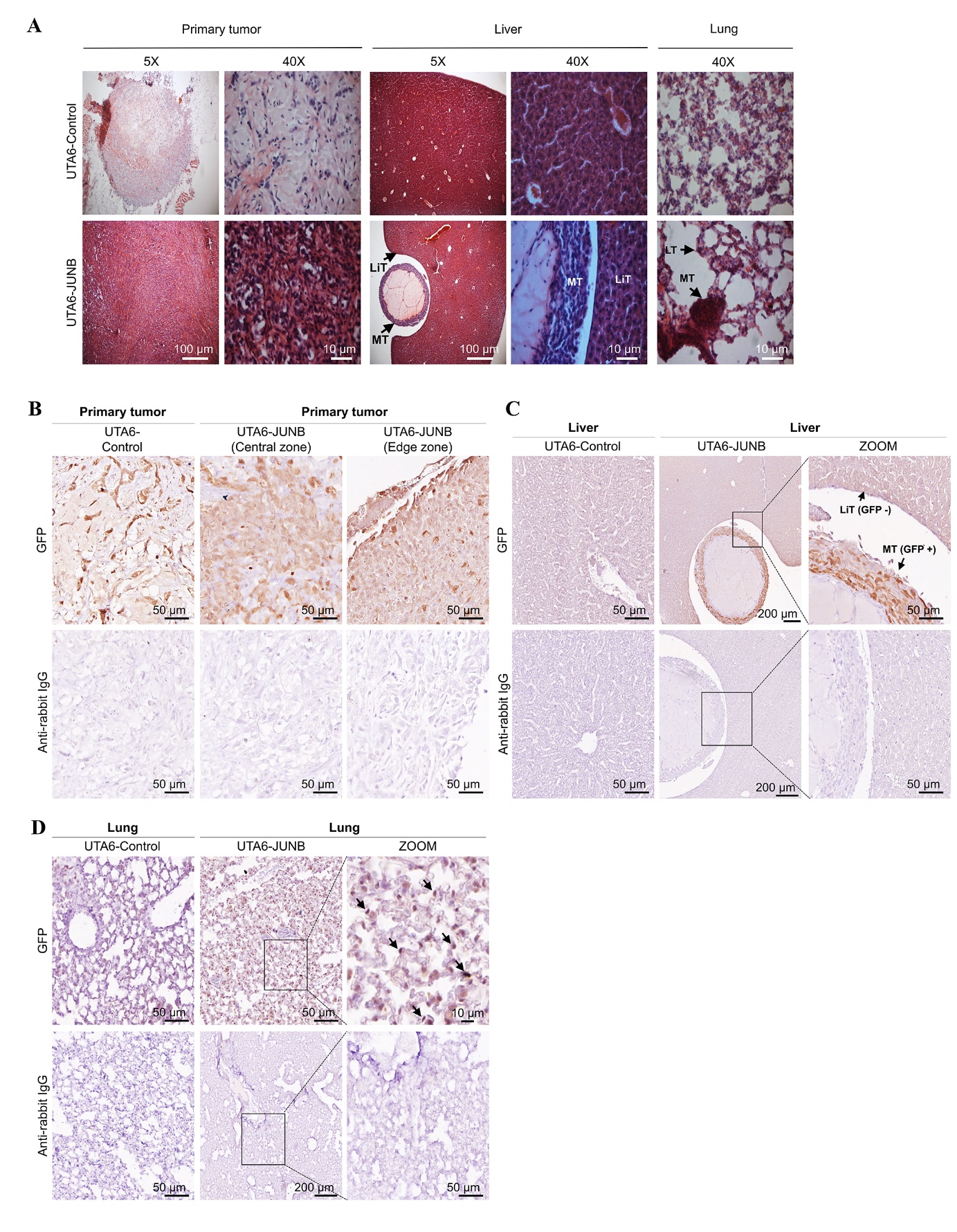


**Fig. S7.** **H&E images and** **IHC images of** **GFP expression in UTA6-Control, UTA6-JUNB primary tumors and metastatic lung and liver lesions.** (**A**) Representative H&E images of UTA6-JUNB and UTA6-Control tumor tissues and JUNB-expressing metastatic lung and liver tissues. (**B**) Representative immunohistochemistry images of GFP expression in the central and peripheral zones of paraffin embedded UTA6-JUNB and UTA6-Control primary tumor tissues. (**C**-**D**) Representative immunohistochemistry images of paraffin embedded UTA6-Control and UTA6-JUNB liver (**C**) and lung metastases (**D**). Arrows indicate GFP positive metastatic cells inside of lung tissue. Staining with an anti-rabbit secondary antibody was used as a negative control. Scale bars are indicated. LT: lung tissue; LiT: liver tissue; MT: metastatic tissue


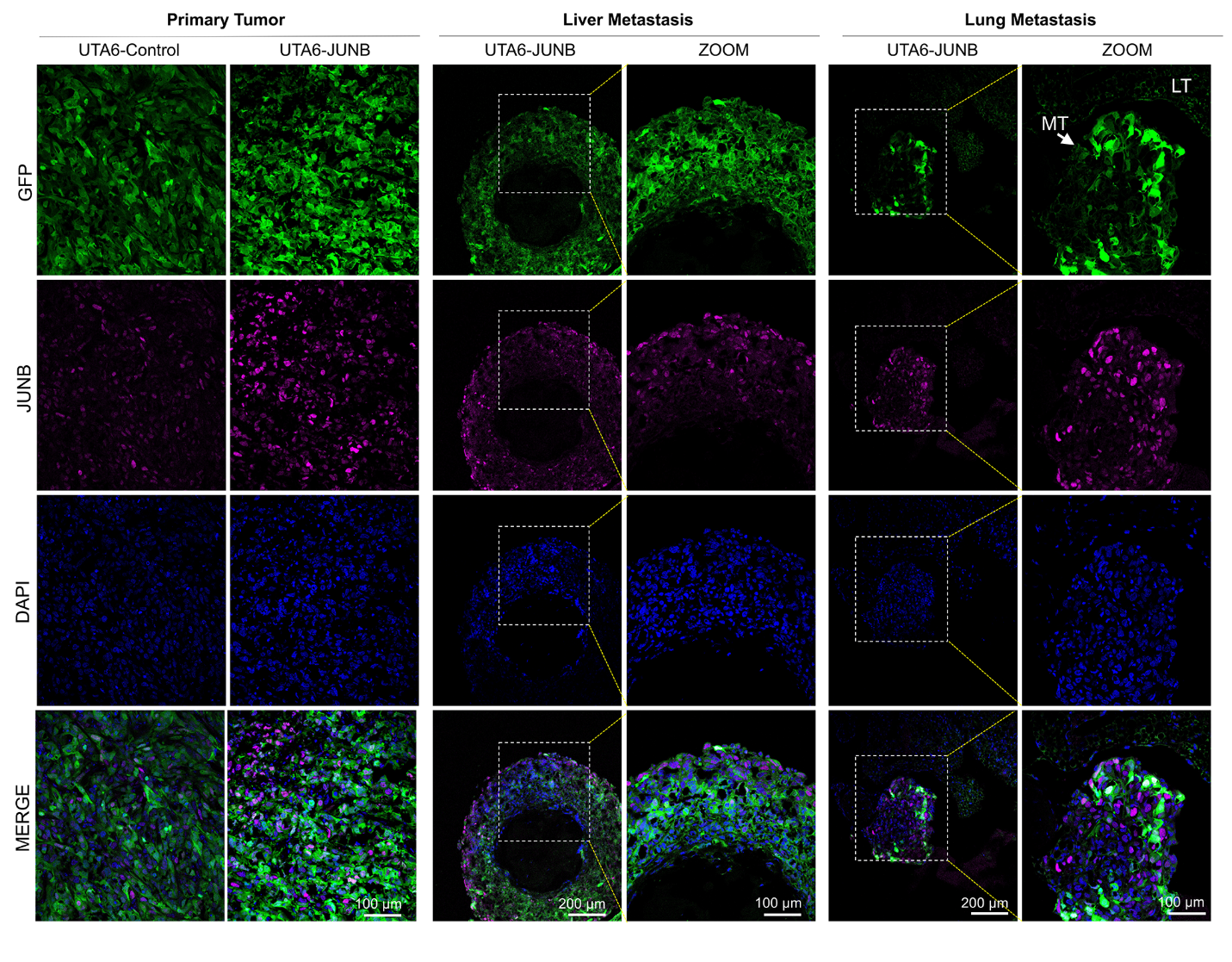


**Fig. 8.** **JUNB and** **GFP expression in UTA6-Control and UTA6-JUNB primary tumors and metastatic lung and liver lesions.**Representative immunofluorescence staining in cryostat sections of UTA6-JUNB and UTA6-Control tumor tissues, as well as of JUNB-expressing metastatic lung and liver tissues. GFP, JUNB, and cell nuclei (blue) are observed. Scale bars are presented. LT: lung tissue; MT: metastatic tissue


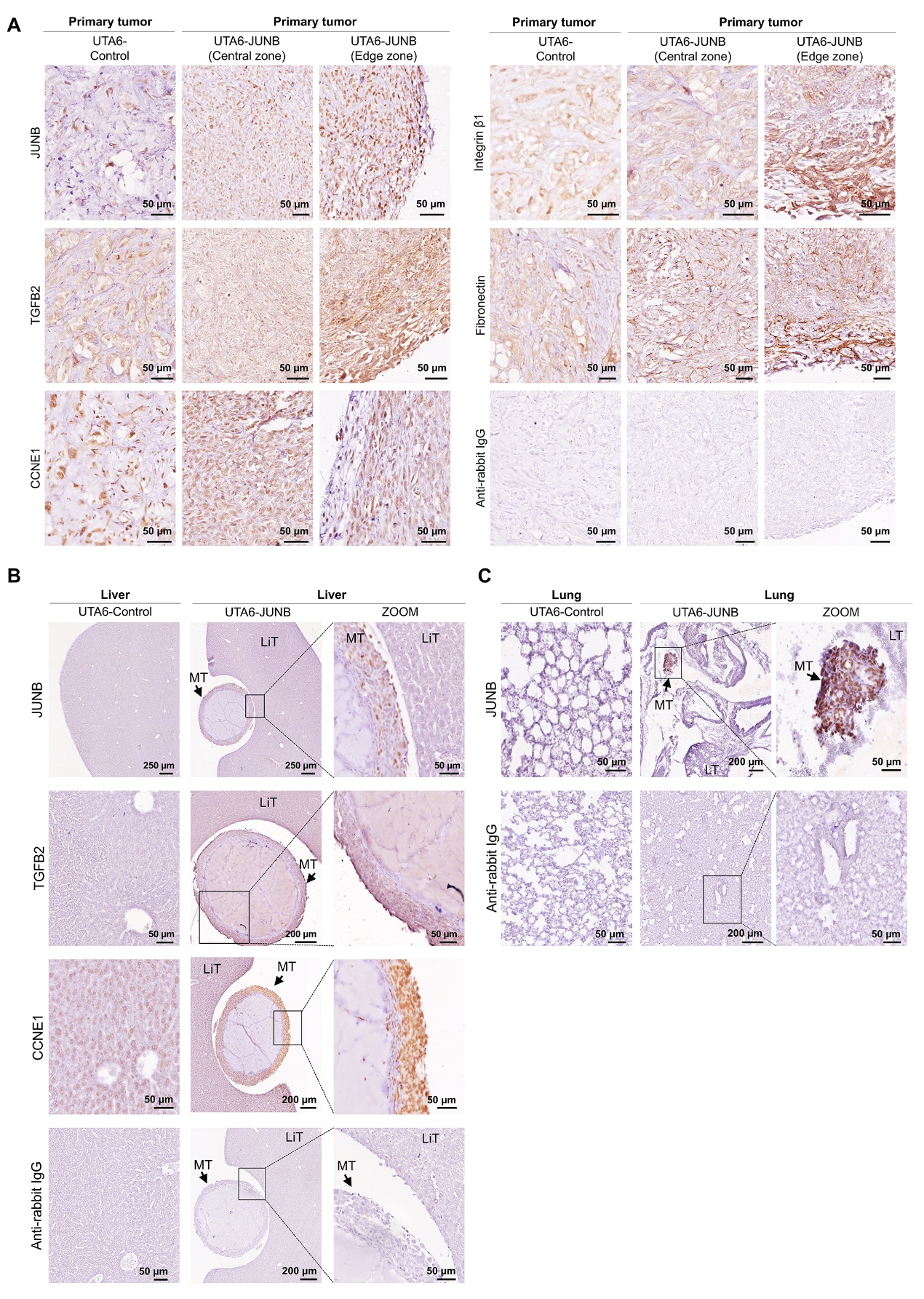


**Fig. S9.** **IHC images of JUNB, TGFB2, CCNE1, Fibronectin and Integrin b1 in UTA6-Control and UTA6-JUNB primary tumors, and of JUNB, TGFB2 and CCNE1 in metastatic lung and liver lesions**. (**A**) *Higher expression of JUNB, TGFB2, Fibronectin and Integrin b1 in the border of UTA6-JUNB primary tumors*. Representative immunohistochemistry images of the central and peripheral zones of paraffin embedded UTA6-Control and UTA6-JUNB primary tumor tissues. (**B**-**C**) *Expression of JUNB, TGFB2 and CCNE1 in metastatic lesions*. Representative immunohistochemistry images of paraffin embedded UTA6-JUNB and UTA6-JUNB liver (**B**) and lung metastases (**C**). Staining with an anti-rabbit secondary antibody was used as a negative control. Scale bars are indicated. LT: lung tissue; LiT: liver tissue; MT: metastatic tissue


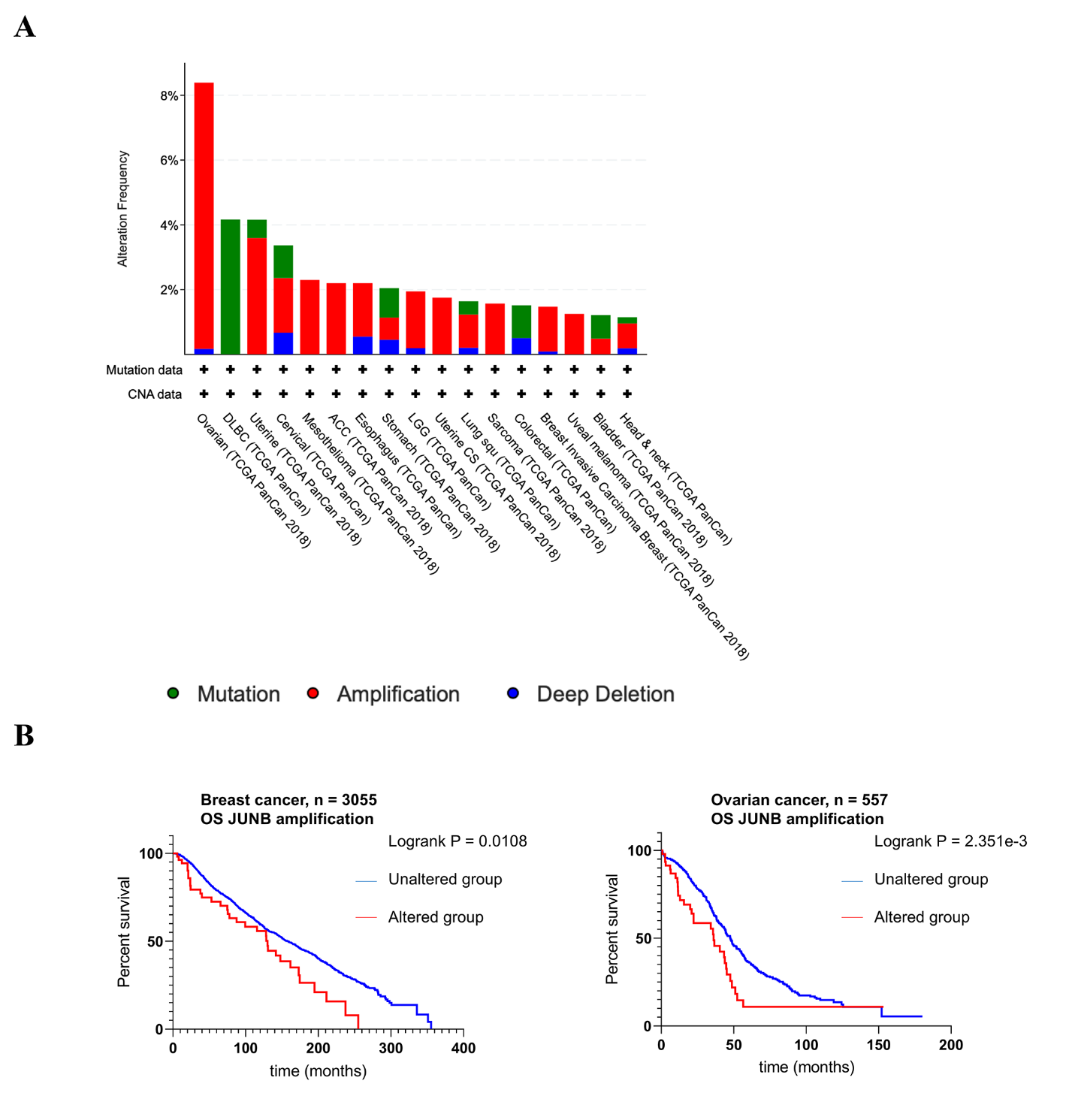


**Fig. S10**. **JUNB amplification in breast and ovarian cancers is associated with poor survival.** (**A**) JUNB copy number alteration (CNA) in several cancers. Genomic alterations of JUNB analysis using the dataset of TCGA Pan-Cancer Atlas Studies at the cBioPortal for Cancer Genomics. Green, mutation; red, amplification; blue, deep deletion. (**B**). Amplification of JUNB is associated with poor survival in ovarian and breast cancers. Survival prognostic curve in patients with ovarian serous cystadenocarcinoma and breast carcinoma.


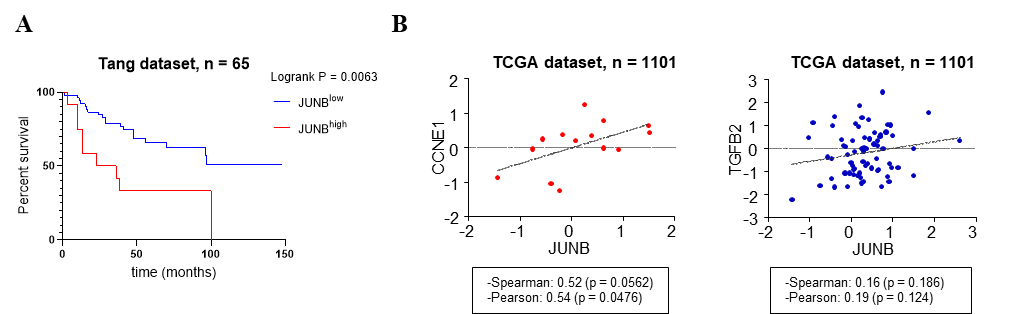
**Fig. S11. Expression of JUNB protein in breast cancer is associated with poor survival** (**A**) Survival prognostic curve in patients with breast cancer with high levels of JUNB protein expression. Patients were grouped by the trichotomization (lower quartile vs upper quartile) value of JUNB (**B**) Correlation between JUNB and CCNE1 or TGF2 protein abundance. The dataset of TCGA (Breast invasive carcinoma, Firehose Legacy) at the cBioPortal for Cancer Genomics was used for the analysis.

**
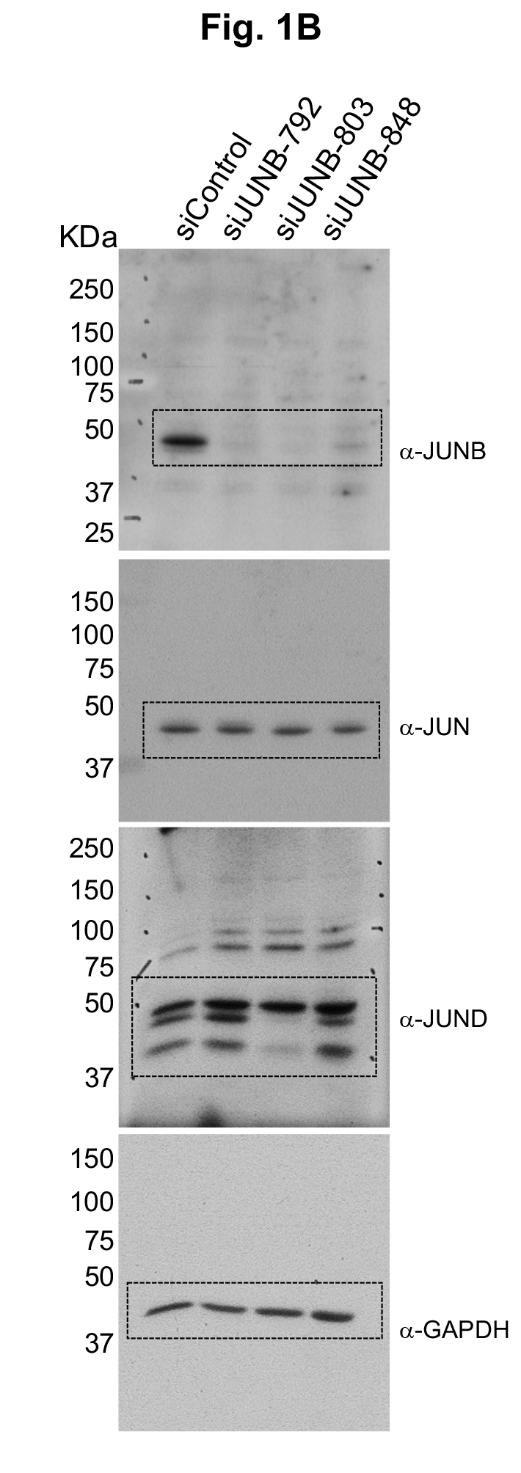
**

**Fig. S12. Uncropped western blot gel images in Figure 1B.** The dotted line boxes highlight lanes used in figures.

**
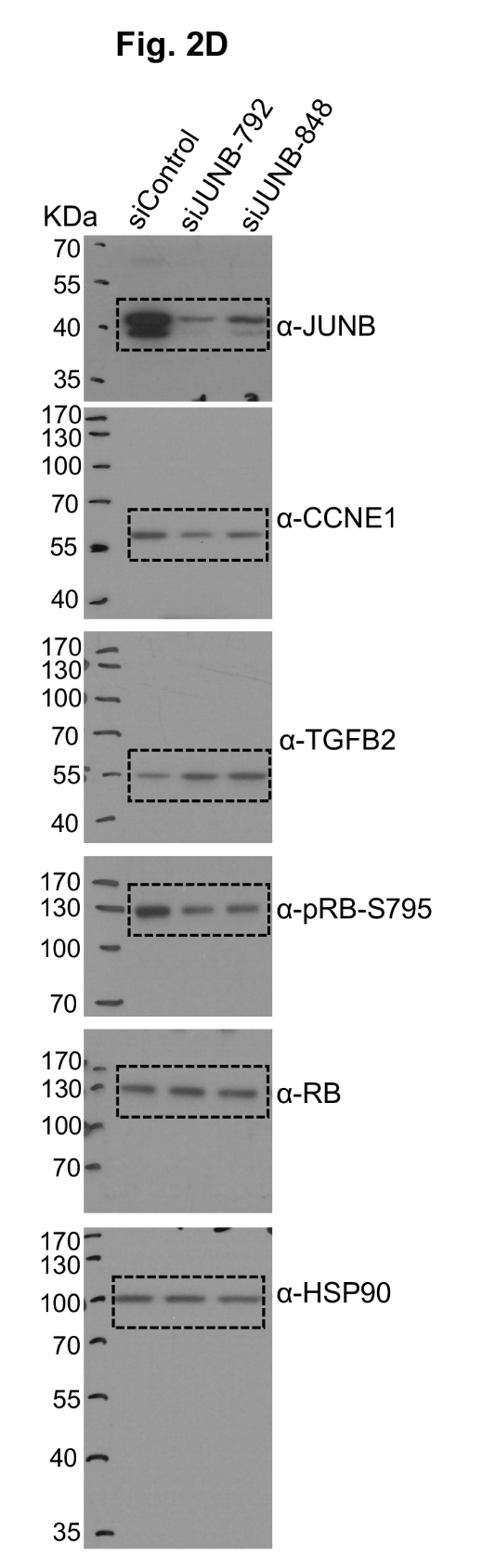
**

**Fig. S13. Uncropped western blot gel images in Figure 2D.** The dotted line boxes highlight lanes used in figures.

**
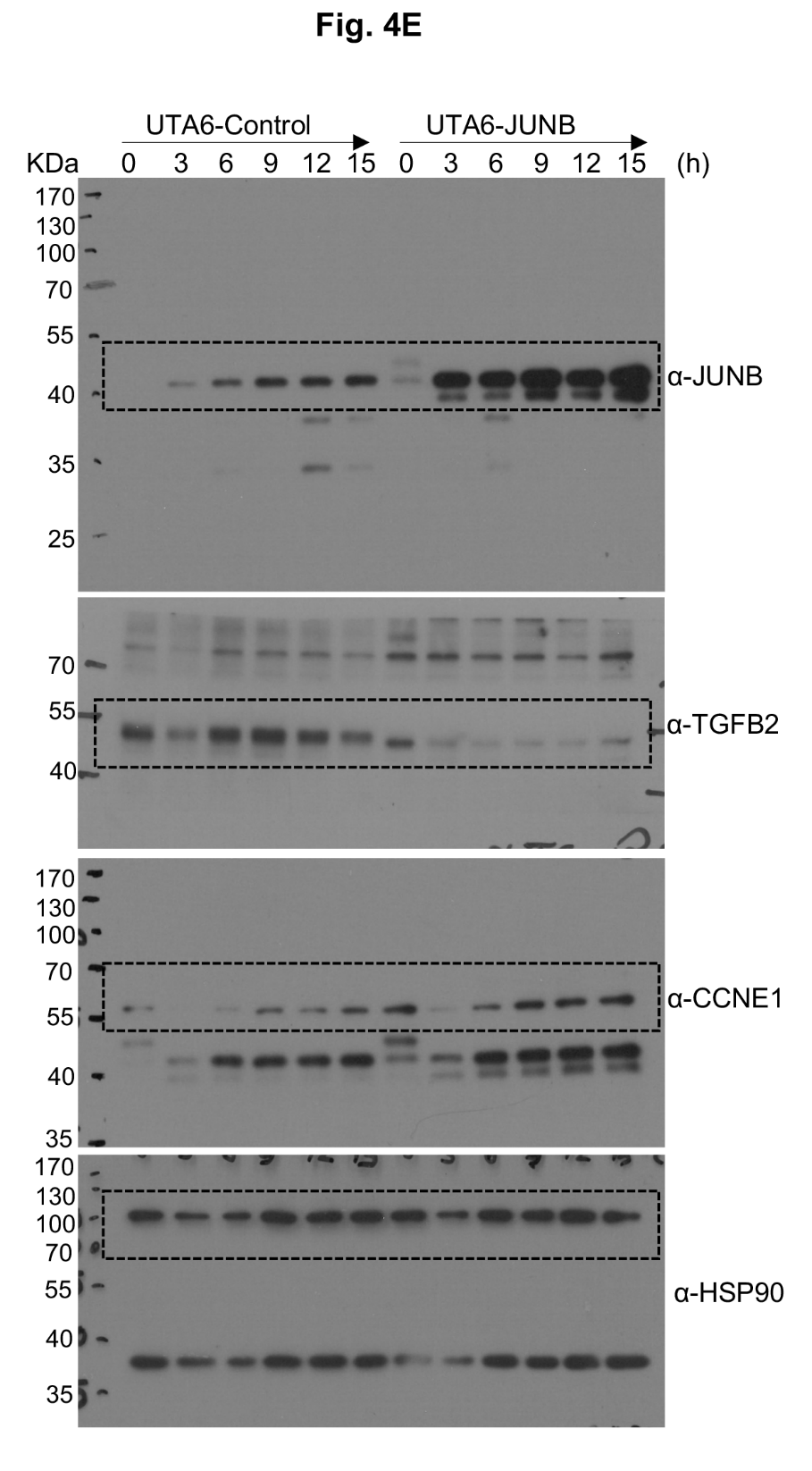
**

**Fig. S14. Uncropped western blot gel images in Figure 4E.** The dotted line boxes highlight lanes used in figures.

**
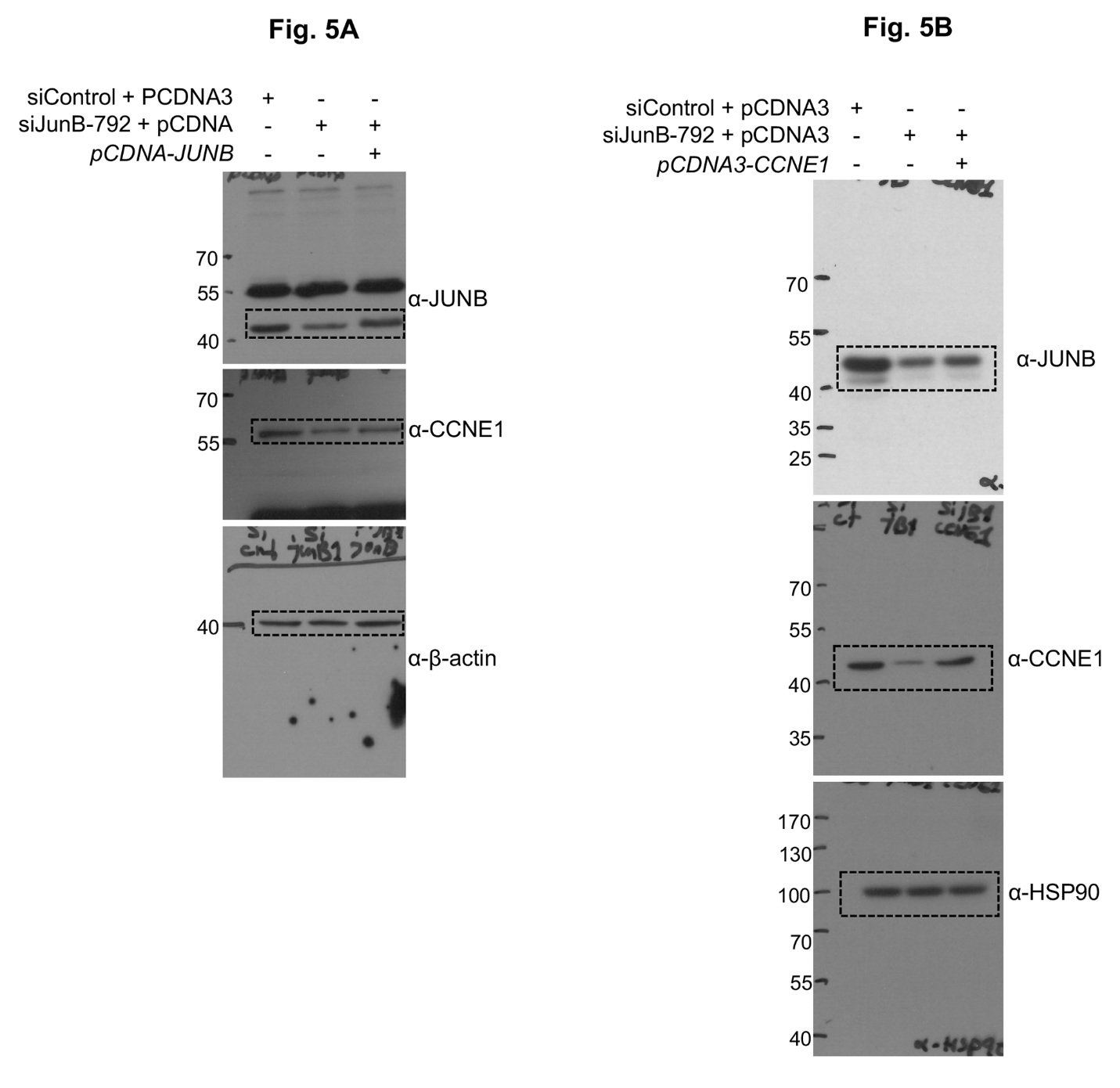
**

**Fig. S15. Uncropped western blot gel images in Figure 5A and 5B.** The dotted line boxes highlight lanes used in figures.

**
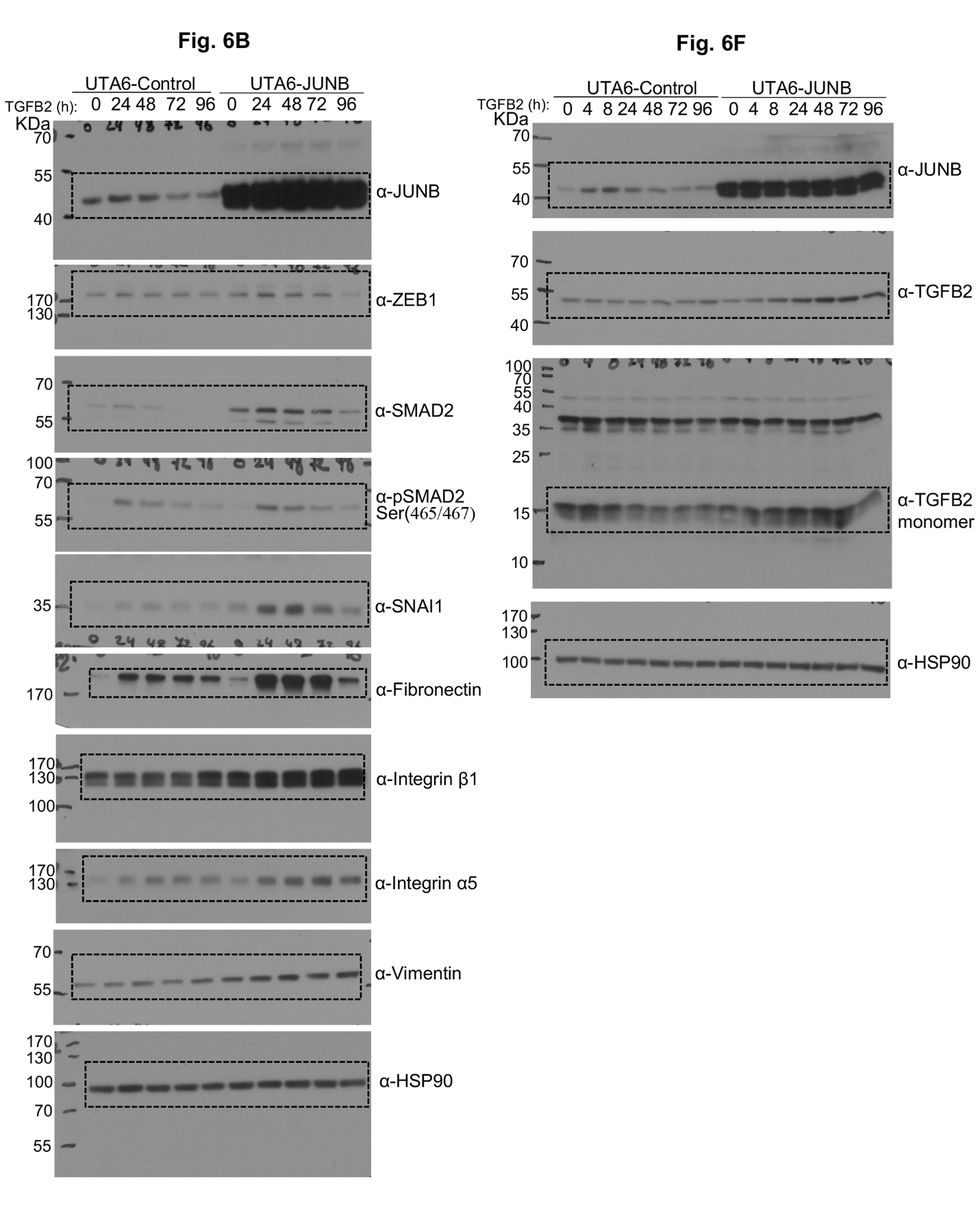
**

**Fig. S16. Uncropped western blot gel images in Figure 6B and 6F.** The dotted line boxes highlight lanes used in figures.

**
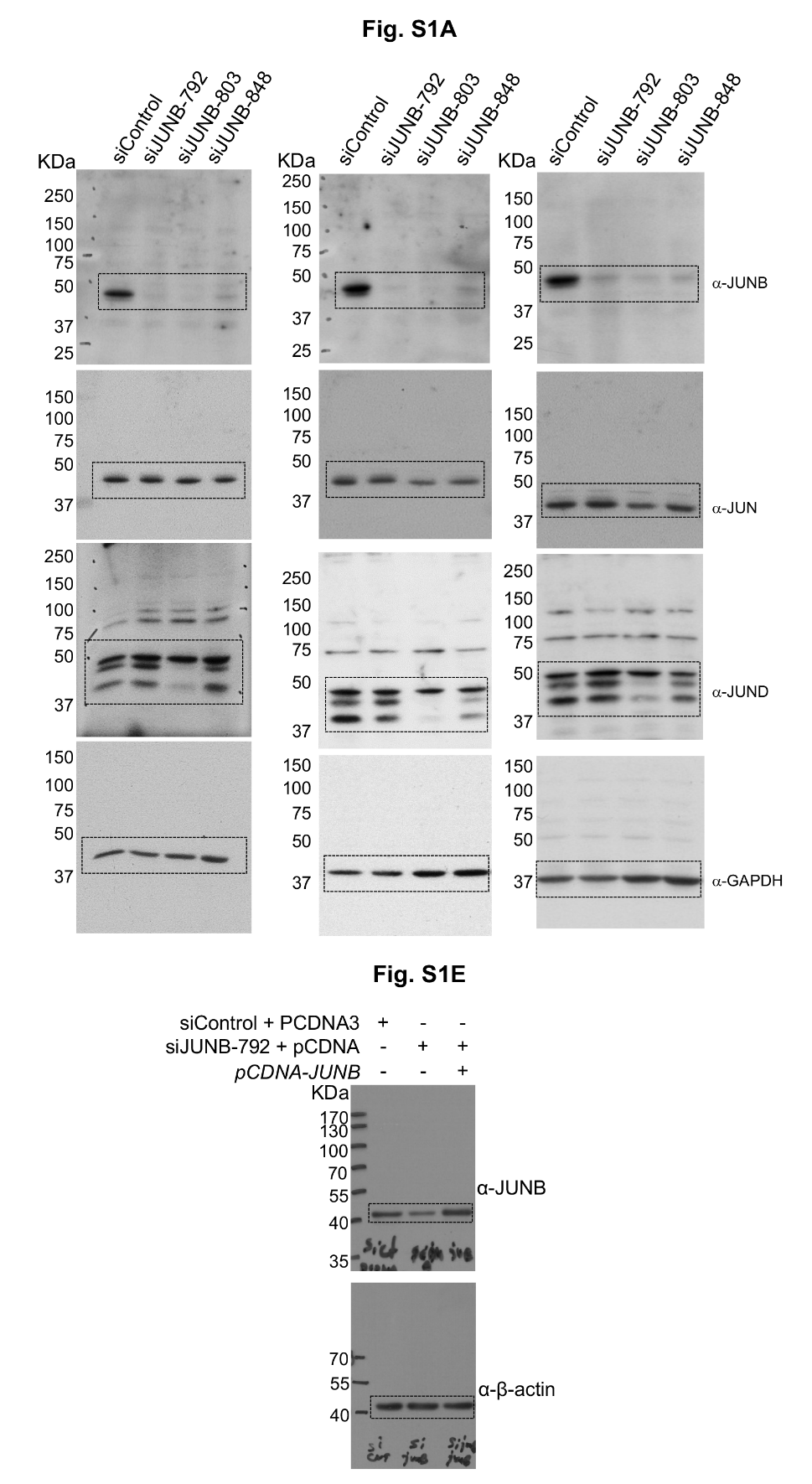
**

**Fig. S17. Uncropped western blot gel images in Figure S1A and S1E.** The dotted line boxes highlight lanes used in figures.

**
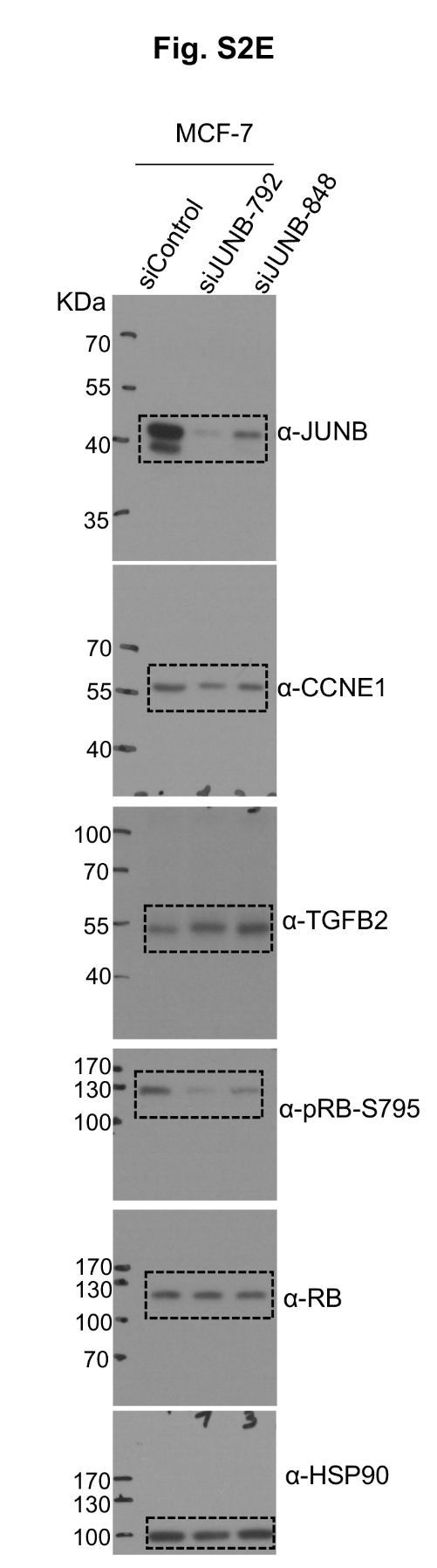
**

**Fig. S18. Uncropped western blot gel images in Figure S2E.** The dotted line boxes highlight lanes used in figures.

**
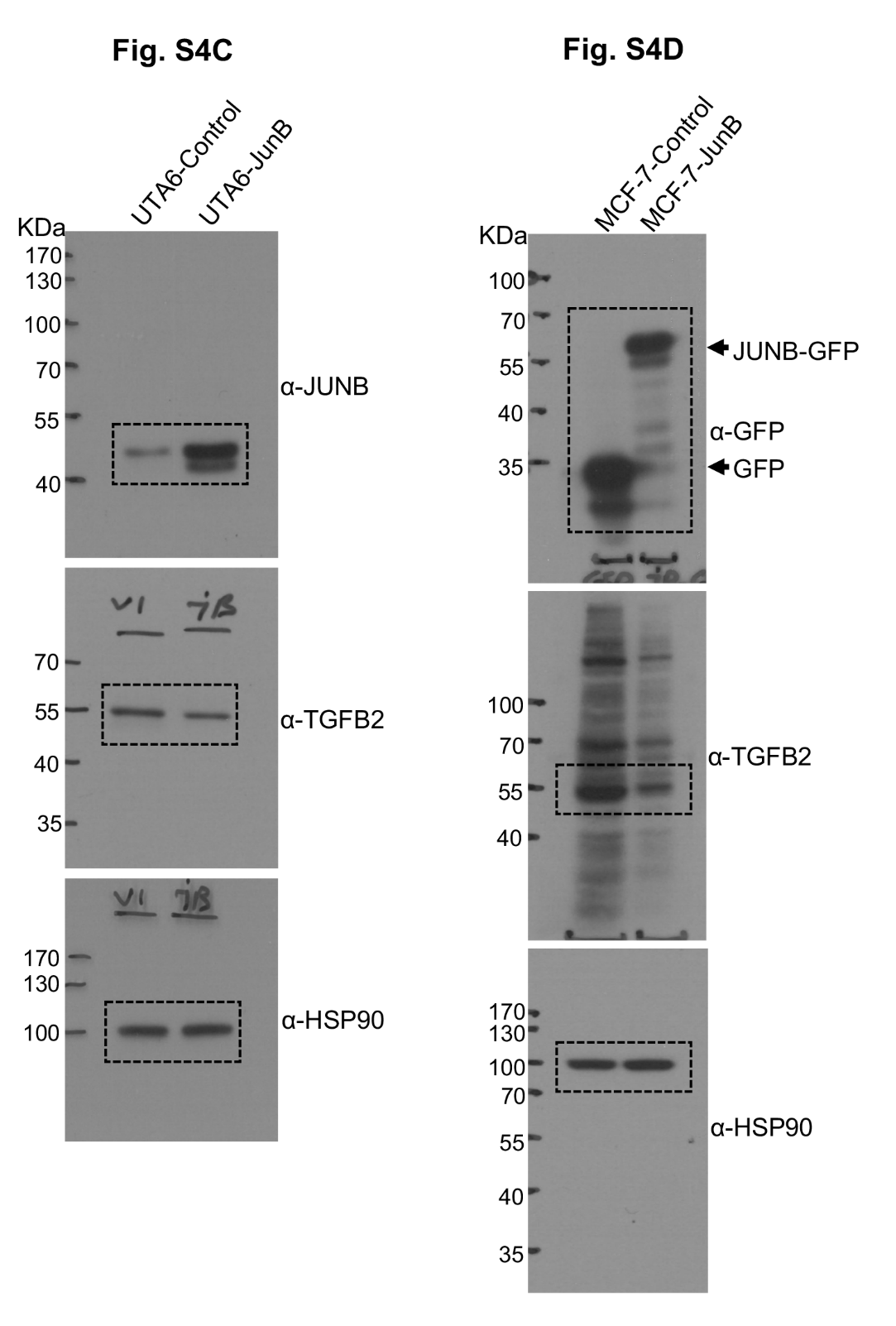
**

**Fig. S19. Uncropped western blot gel images in Figure S4C and S4D.** The dotted line boxes highlight lanes used in figures.

**
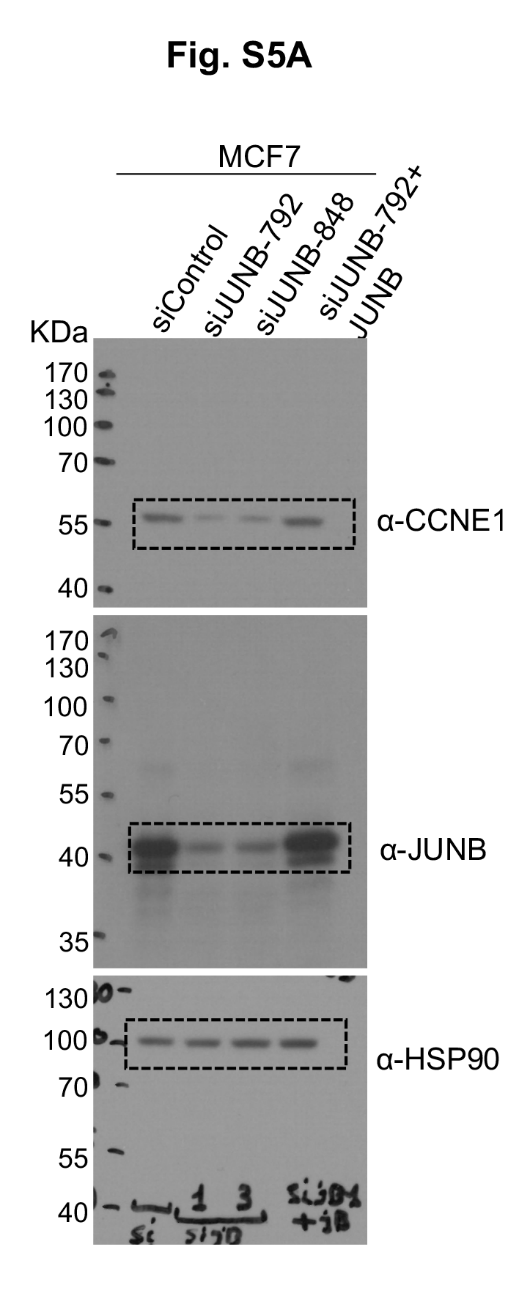
**

**Fig. S20. Uncropped western blot gel images in Figure S5A.** The dotted line boxes highlight lanes used in figures.

**
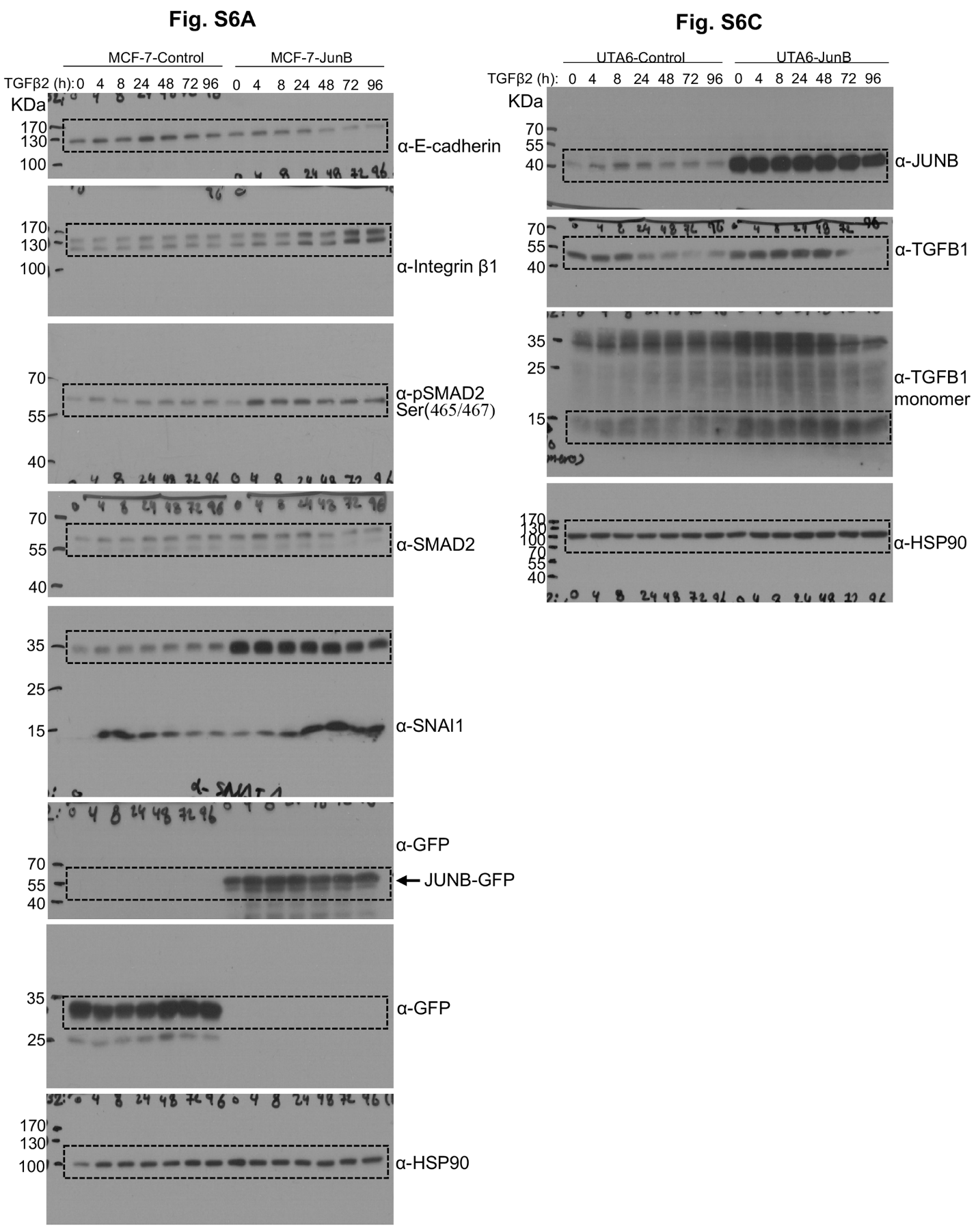
**

**Fig. S21. Uncropped western blot gel images in Figure S6A and S6C.** The dotted line boxes highlight lanes used in figures.

**SUPPLEMENTAL METHODS**

**Cell proliferation by living cell imaging**

Proliferation rate was quantified by measuring cell confluence values of phase contrast images acquired by time-lapse microscopy with Cytation 5 cell imaging system (Biotek). 1 x 10^4^ cells were seeded in 12-well culture plate and day after cells transfected with siRNA. Microscopy phase contrast images were acquired at the time of transfection (time 0 h) and for the indicated times. For cells stably overexpressing JUNB, 1 x 10^4^ cells were seeded in 12-well culture plate and microscopy phase contrast images were acquired 6 h after seeding (time 0 h) and for the indicated times. The area occupied by the cells related to total image area of 10 random images of each time was calculated with Gen5 software and related to its value at time 0 h.

**Immunohistochemistry**

Tissue sections were deparaffined and rehydrated through graded methanol, rinsed in distilled water. The samples were incubated for 10 minutes with peroxidase blocking solution (Dako). Antigen retrieval was performed by boiling in a pressure cooker for 3 min at 1.5 atm in Envision™ FLEX Target Retrieval Solution high pH Tris/EDTA, pH 9 (Dako). Then, samples were incubated overnight with primary antibodies prepared in EnVision FLEX Antibody Diluent (Dako). Secondary anti rabbit antibody (1:200) was incubated for 2 hours and after washing with PBS, Dako envision amplification system (Cytomation Envision System-labelled polymer-HRP anti-mouse) was used, followed by staining with 3,3′-diaminobenzidine (Dako) as chromogen according to the manufacturer’s instructions, which originated a brown staining in immunoreactive structures. Sections were finally counterstained with Mayer’s hematoxylin (SigmaAldrich).
