# Supplementary material for: New roles for AP-1/JUNB in cell cycle control and tumorigenic cell invasion via regulation of cyclin E1 and TGF-β2
Source: Genome Biol. 2022 Dec 9;23:252. doi: 10.1186/s13059-022-02800-0 (PMC9733061; doi:10.1186/s13059-022-02800-0)
Supplement: Supplementary file 3 — Additional file 3: Table S2. High-confidence JUNB regulated genes involved in cell cycle regulation and E2F pathway. [file 13059_2022_2800_MOESM3_ESM.docx]

| **Cell cycle genes** | **Fold change**  **siJUNB-792 vs siControl** | **Fold change**  **siJUNB-848 vs siControl** | **JUNB binding sites (bases from TSS)** | **AP-1/**  **TRE motifs** | **AP-1/**  **CRE motifs** | **cAE** | **cAP** | **cIE** |
| --- | --- | --- | --- | --- | --- | --- | --- | --- |
| *CCNE1* | -2,48 | -1,61 |  |  |  |  |  |  |
| *FZD3* | -2,32 | -1,48 |  |  |  |  |  |  |
| *YEATS4* | -2,06 | -2,39 | 45891 | 1 | 1 |  |  |  |
| *IL8* | -1,95 | -2,17 | -35586  -14304 | 1  1 |  |  |  |  |
| *ERCC2* | -1,49 | -1,37 | -6123 |  | 1 | ✓ |  |  |
| *FGF5* | -1,49 | -2,35 |  |  |  |  |  |  |
| *SLX4* | -1,41 | -1,58 |  |  |  |  |  |  |
| *DCUN1D3* | -1,38 | -1,46 | -10385 | 1 |  |  |  |  |
| *EXO1* | -1,34 | -1,25 |  |  |  |  |  |  |
| *PRDM5* | -1,33 | -1,46 | 227606 | 1 |  |  |  |  |
| *TIMELESS* | -1,31 | -1,39 |  |  |  |  |  |  |
| *THOC5* | 1,30 | 1,32 |  |  |  |  |  |  |
| *GAS2L1* | 1,31 | 1,36 | 4085 | 1 |  |  |  |  |
| *ZFP36L2* | 1,33 | 1,63 |  |  |  |  |  |  |
| *CAB39L* | 1,34 | 1,37 |  |  |  |  |  |  |
| *ZAK* | 1,49 | 1,48 | -26514 | 1 |  |  |  | ✓ |
| *GPNMB* | 1,52 | 1,45 |  |  |  |  |  |  |
| *PLCB1* | 1,55 | 1,50 |  |  |  |  |  |  |
| *RPTOR* | 1,61 | 1,55 | -12175  75196 | 1  1 |  | ✓ |  |  |
| *BANF1* | 1,65 | 1,57 |  |  |  |  |  |  |
| *RPS15A* | 1,72 | 1,61 |  |  |  |  |  |  |
| *TGFB2* | 1,73 | 1,81 | 31417  114007  134547  150759  239277  313038  314803  359953 | 1  1  1  1  1  1  1 | 1  1 | ✓ |  | ✓ |
| *SIRT7* | 1,92 | 1,53 |  | 1 |  |  |  |  |
| *CITED2* | 2,10 | 1,77 | -4425  -33286 | 1 |  |  |  |  |
| **E2F target genes** | **Fold change**  **siJUNB-792 vs siControl** | **Fold change**  **siJUNB-848 vs siControl** | **JUNB binding sites (bases from TSS)** | **AP1/**  **TRE motifs** | **AP1/**  **CRE motifs** | **cAE** | **cAP** | **cIE** |
| *JUNB* | -2,56 | -1,66 |  |  |  |  |  |  |
| *CCNE1* | -2,48 | -1,61 |  |  |  |  |  |  |
| *FZD3* | -2,32 | -1,48 |  |  |  |  |  |  |
| *CTNNBIP1** | -1,74 | -2,20 |  |  |  |  |  |  |
| *ZMYM4* | -1,47 | -1,41 |  |  |  |  |  |  |
| *CITED1* | -1,43 | -1,46 |  |  |  |  |  |  |
| *VEGFC* | -1,35 | -1,38 |  |  |  |  |  |  |
| *EXO1*** | -1,34 | -1,25 |  |  |  |  |  |  |
| *TIMELESS** | -1,31 | -1,39 |  |  |  |  |  |  |
| *EIF3B*** | 1,27 | 1,29 |  |  |  |  |  |  |
| *NTN1**** | 1,42 | 1,52 | 29496 | 1 |  |  |  |  |
| *RBPJ* | 1,57 | 1,33 | -79350  -639  34053  58342 | 1  1  1 | 1 |  | ✓ | ✓ |
| *TCEB3* | 1,64 | 1,51 |  |  |  |  |  |  |
| *JAG1*** | 1,66 | 2,45 |  |  |  |  |  |  |
| *BLMH* | 1,70 | 1,56 |  |  |  |  |  |  |
| *TGFB2* | 1,73 | 1,81 | See above |  |  |  |  |  |
| *GSTM2* | 1,74 | 2,02 |  |  |  |  |  |  |
| *H3F3A* | 1,88 | 2,08 |  |  |  |  |  |  |
| *GCH1* | 1,9 | 1,37 |  |  |  |  |  |  |
| *CITED2* | 2,10 | 1,77 | See above |  |  |  |  |  |

**Table S2.** **High-confidence JUNB regulated genes involved in cell cycle regulation and E2F pathway.** Fold change in mRNA expression of JUNB-regulated genes in siJUNB-792 or siJUNB-848 vs siControl transfected cells*.* Genes with a well-documented role in cell cycle were collected from AmiGO [72], Panther [73] and GSEA [33]. E2F target genes were collected from AmiGO [72], Panther [73], GSEA [33], ENCODE [74] and references [34, 40]. JUNB binding sites identified in the ChIP-seq analysis are also indicated. JUNB binding sites associated with either active promoter (cAP), active enhancer (cAE), inactive promoters (cIP) or inactive enhancers (cIE) regions as defined in the text are shown. *E2F1 target; **E2F1 and E2F3 target; ***E2F3 target. The rest of the indicated genes are targets of E2F1, E2F2 and E2F3.
