# Supplementary material for: New roles for AP-1/JUNB in cell cycle control and tumorigenic cell invasion via regulation of cyclin E1 and TGF-β2
Source: Genome Biol. 2022 Dec 9;23:252. doi: 10.1186/s13059-022-02800-0 (PMC9733061; doi:10.1186/s13059-022-02800-0)
Supplement: Supplementary file 4 — Additional file 4: Table S3. High-confidence JUNB regulated genes involved in EMT and TGFB signalling. [file 13059_2022_2800_MOESM4_ESM.docx]

| **EMT and TGFB signalling genes** | **Fold change**  **siJUNB-792 vs siControl** | **Fold change**  **siJUNB-848 vs siControl** | **JunB binding sites (bases from TSS)** | **AP-1/TRE motifs** | **AP-1/CRE motifs** | **cAE** | **cAP** | **cIE** |
| --- | --- | --- | --- | --- | --- | --- | --- | --- |
| *JUNB* | -2,56 | -1,66 |  |  |  |  |  |  |
| *IL8* | -1,95 | -2,17 | see Table S2 |  |  |  |  |  |
| *ETV4* | -1,91 | -1,65 | -33327  -45761  -24136 | 1  1 | 1  1 | ✓  ✓  ✓ |  |  |
| *CORO2B* | -1,80 | -1,59 | 7457 |  |  | ✓ |  |  |
| *RELL1* | -1,80 | -1,60 | -7065  38088 | 1  1 |  |  |  |  |
| *CTNNBIP1* | -1,74 | -2,20 |  |  |  |  |  |  |
| *EFR3B* | -1,73 | -3,44 |  |  |  |  |  |  |
| *FHOD3* | -1,66 | -1,35 | 11996 | 1 |  |  |  |  |
| *EDNRA* | -1,65 | -1,52 |  |  |  |  |  |  |
| *SLFN5* | -1,57 | -1,58 |  |  |  |  |  |  |
| *RAB8B* | -1,49 | -1,51 |  |  |  |  |  |  |
| *MAML3* | -1,47 | -1,35 |  |  |  |  |  |  |
| *DIRC2* | -1,46 | -2,38 |  |  |  |  |  |  |
| *CITED1* | -1,43 | -1,47 |  |  |  |  |  |  |
| *ZFYVE9* | -1,43 | -1,62 |  |  |  |  |  |  |
| *ETV1* | -1,44 | -1,40 |  |  |  |  |  |  |
| *VEGFC* | -1,35 | -1,38 |  |  |  |  |  |  |
| *CDH4* | -1,34 | -1,47 | 32345  -66256  -62833 | 1  1 | 1 | ✓ |  |  |
| *TAPT1* | 1,30 | 1,37 |  |  |  |  |  |  |
| *ZNF451* | 1,30 | 1,98 |  |  |  |  |  |  |
| *SNIP1* | 1,31 | 1,31 | 19712 | 1 |  |  |  |  |
| *PLS3* | 1,31 | 1,47 |  |  |  |  |  |  |
| *LAMA5* | 1,35 | 1,48 |  |  |  |  |  |  |
| *NTN1* | 1,42 | 1,52 | 29496 | 1 |  |  |  |  |
| *NEBL* | 1,42 | 1,67 |  |  |  |  |  |  |
| *PGM2L1* | 1,51 | 1,94 |  |  |  |  |  |  |
| *PTPN14* | 1,51 | 1,66 | 104940 | 1 |  |  |  |  |
| *TSPAN2* | 1,52 | 2,39 | -100688  -90263  -79238  -46203 | 1  1  1  1 | 1 | ✓  ✓ |  |  |
| *TMEFF1* | 1,54 | 1,47 |  |  |  |  |  |  |
| *RBPJ* | 1,57 | 1,33 | see Table S2 |  |  |  |  |  |
| *JAG1* | 1,66 | 2,45 |  |  |  |  |  |  |
| *TGFB2* | 1,73 | 1,81 | see Table S2 |  |  |  |  |  |
| *SNAI2* | 1,76 | 1,92 |  |  |  |  |  |  |
| *CITED2* | 2,10 | 1,77 | see Table S2 |  |  |  |  |  |
| *CALU* | 2,16 | 2,24 |  |  |  |  |  |  |
| *SLC2A3* | 3,52 | 1,80 |  |  |  |  |  |  |

**Table S3.** **High-confidence JUNB regulated genes involved in EMT and TGFB signalling.** Fold change in mRNA expression of JUNB target genes in siJUNB-792 or siJUNB-848 vs siControl and JUNB binding sites for EMT and TGFB signalling target genes*.* JunB binding sites identified in the ChIP-seq analysis are also indicated. Genes with a well-documented role in epithelial- mesenchymal transition and TGFβ signalling were collected from AmiGO [72], Panther [73] GSEA [33], ENCODE [74] and [31]. JUNB binding sites identified in the ChIP-seq analysis are also indicated. JUNB binding sites associated either at active promoter (cAP), active enhancer (cAE), inactive promoters (cIP) or inactive enhancers (cIE) regions as defined in the text are shown.
