# Supplementary material for: New roles for AP-1/JUNB in cell cycle control and tumorigenic cell invasion via regulation of cyclin E1 and TGF-β2
Source: Genome Biol. 2022 Dec 9;23:252. doi: 10.1186/s13059-022-02800-0 (PMC9733061; doi:10.1186/s13059-022-02800-0)
Supplement: Supplementary file 7 — Additional file 7: Table S6. List of antibodies used for western blot, immunofluorescence and immunohistochemistry. [file 13059_2022_2800_MOESM7_ESM.docx]

| **Antibody Target** | **Host** | **Dilution**  **WB** | **Dilution**  **IF/IHC** | **Company**  **(Catalog#)** |
| --- | --- | --- | --- | --- |
| β-actin | Mouse | 1:5000 | - | Sigma-Aldrich (A5441) |
| FOS | Rabbit | 1:1000 | - | SCBT (sc52-G) |
| JUN | Rabbit | 1:1000 | - | SCBT (sc-1694) |
| Cyclin E1 | Rabbit | 1:1000 | - | SCBT (sc-596) |
| CCNE1 | Rabbit | - | 1:100 | Sigma-Aldrich (HPA018169) |
| Fibronectin | Rabbit | 1:3000 | 1:150 | GeneTex (GTX112794) |
| GAPDH | Mouse | 1:5000 | - | SCBT (sc-47724) |
| GFP | Rabbit | 1:1000 | 1:150 | Rockland (600-401-215) |
| HSP90 | Mouse | 1:5000 | - | SCBT (sc-13119) |
| Integrin α5 | Rabbit | 1:3000 | - | GeneTex (GTX130705) |
| Integrin β1 | Rabbit | 1:3000 | 1:200 | GeneTex (GTX128839) |
| JUNB | Rabbit | 1:1000 | 1:100 | Cell Signaling (#3753) |
| JUNB | Mouse |  | 1:200 | [12] |
| JUND | Rabbit | 1:1000 | - | SCBT (sc-74) |
| pRb-S795 | Rabbit | 1:1000 | - | Cell Signaling (#9301) |
| pSMAD2(Ser465/467) | Rabbit | 1:1000 | - | GeneTex (GTX133614) |
| Rb | Mouse | 1:2000 | - | Cell Signaling (#9309) |
| SMAD2/3 | Mouse | 1:1000 | - | SCBT (sc-133098) |
| SNAI1 | Rabbit | 1:1000 | - | GeneTex (GTX125918) |
| TGFB1 | Rabbit | 1:1000 | - | Abcam (ab92486) |
| TGFB1 | Rabbit | 1:1000 | - | GeneTex (GTX130023 |
| TGFB2 | Rabbit | 1:2000 | - | GeneTex (GTX132546) |
| TGFB2 (clone 1D11) | Mouse | 1:1000 | - | R&D Systems |
| TGFB2 | Rabbit | - | 1:50 | Abcam(ab53778) |
| Vimentin | Mouse | 1:7000 | - | Millipore (MAB1633) |
| ZEB1 | Rabbit | 1:1000 | - | GeneTex (GTX105278) |
| Anti-Mouse IgG (whole molecule)–Peroxidase | Rabbit | 1:5000 | - | Sigma-Aldrich (A9044) |
| Anti-Rabbit IgG (whole molecule)–Peroxidase | Goat | 1:5000 | - | Sigma-Aldrich (A6154) |
| Goat anti-Mouse IgG (H+L) Cross-Adsorbed Secondary Antibody Alexa Fluor 488 | Goat | - | 1:400 | Invitrogen(A11001) |
| Goat anti-Rabbit IgG (H+L) Cross-Adsorbed Secondary Antibody Alexa Fluor 488 | Goat | - | 1:400 | Invitrogen(A11008) |
| F(ab')2-Goat anti-Rabbit IgG (H+L) Cross-Adsorbed Secondary Antibody, Alexa Fluor 633 | Goat | - | 1:400 | Invitrogen(A21072) |

**Table S6.** List of antibodies
