# Supplementary material for: New roles for AP-1/JUNB in cell cycle control and tumorigenic cell invasion via regulation of cyclin E1 and TGF-β2
Source: Genome Biol. 2022 Dec 9;23:252. doi: 10.1186/s13059-022-02800-0 (PMC9733061; doi:10.1186/s13059-022-02800-0)
Supplement: Supplementary file 8 — Additional file 8: Table S7. List of primers used for real-time PCR. [file 13059_2022_2800_MOESM8_ESM.docx]

| **Primer name** | **Primer sequence (5´-3´)** |
| --- | --- |
| *CCNE1 F* | GGTATACTTGCTGCTTCGGCC |
| *CCNE1 R* | AGTTTTGAGCTCCCCGTCTCC |
| *CITED2 F* | GAGGGCAACGGAGGGGAAATA |
| *CITED2 R* | CCAGTCCTTCCGTTTTTGCGA |
| *ERCC2 F* | GTCCCTGCTGCATACTCTGGA |
| *ERCC2 R* | GTTCTGTCGTCAAAGGGCTCG |
| *FGF5 F* | ACTGAAAAAACAGGGCGGGAG |
| *FGF5 R* | TGGGTAGAGATATGCTGGGGT |
| *FZD3 F* | GACAGCAGCTTTGGCAATGGA |
| *FZD3 R* | GAACACTCACTGTAAGCCCGC |
| *GAS2L1 F* | CTCATCTTTGTGCGGGTGCTG |
| *GAS2L1 R* | CGATGAGCAGTGGAGGAGCAG |
| *GUSB F* | GGAGCAGAAACGATTGCAGGG |
| *GUSB R* | TATTCCCCAGCACTCTCGTCG |
| *IL8 F* | AACCACCGGAAGGAACCATCT |
| *IL8 R* | AACTGCACCTTCACACAGAGC |
| *hJUNB F* | CTGGTGGCCTCTCTCTACACG |
| *hJUNB R* | CCCGCGGGGGTAAAAGTACTG |
| *mJUNB F* | TCCCTATCGGGGTCTCAAGG |
| *mJUNB R* | TAGCTTCAGAGATGCGCCTG |
| *PIK3CD F* | ACAGATCAGCCTCCTCATCGG |
| *PIKECD R* | CTGCAGGGGGAAACTGTACTG |
| *RPTOR F* | GCTGGAGGATGAAGGATCGGA |
| *RPTOR R* | GAGGACCCATCGACAGAGGAT |
| *S26* | CTGCACTAACTGTGCCCGATGCGTG |
| *S26* | GACGCTCGCTTCAGAAATGTCCCTG |
| *TGFB2 F* | AAGCTTACACTGTCCCTGCTG |
| *TGFB2 R* | ACTGGTATATGTGGAGGTGCC |
| *TGFB2-A ChIP F* | GCAACTCACCCCCTTCCTTT |
| *TGFB2-A ChIP R* | CCAGTAGCTGCCATTCCACA |
| *TGFB2-B ChIP F* | GAACCCAGTGCTTTACGTGG |
| *TGFB2-B ChIP R* | GAGGGATGCCCTTGTGGTTA |
| *TGFB2-C ChIP F* | AAACAGACCCGGTAAAAGCC |
| *TGFB2-C ChIP R* | TGGTAGTGAGTCATCCCACAAC |
| *CCNE1-A ChIP F* | TGCAAAGCCTTCTTCCGTCT |
| *CCNE1-A ChIP R* | AGCCACCTCACCCTATGTCT |
| *CCNE1-B ChIP F* | GACTGATGTAAGCGTGTGGC |
| *CCNE1-B ChIP R* | GTTGGCCCCTCATGTGGAAG |
| *CCNE1-C ChIP F* | TCTGCCATCTTGGACACAACT |
| *CCNE1-C ChIP R* | GCAGTGTAGCATGATAGGGCT |

Forward primer, F; Reverse primer, R.

**Table S7.** List of primers
