# Supplementary material for: New roles for AP-1/JUNB in cell cycle control and tumorigenic cell invasion via regulation of cyclin E1 and TGF-β2
Source: Genome Biol. 2022 Dec 9;23:252. doi: 10.1186/s13059-022-02800-0 (PMC9733061; doi:10.1186/s13059-022-02800-0)
Supplement: Supplementary file 9 — Additional file 9: Table S8. List of siRNA. [file 13059_2022_2800_MOESM9_ESM.docx]

| **Primer name** | **Primer sequence (5´-3´)** |
| --- | --- |
| siJUNB-792: sense | AGACCAAGAGCGCAUCAAA |
| siJUNB-848 | NM_002229 SASI_Hs01_00204774, Sigma |
| siJUNB-803 | NM_002229 SASI_Hs01_00204768, Sigma |
| Silencer negative control | AM4611, Ambion |

**Table S8.** List of siRNA
