# Supplementary material for: New roles for AP-1/JUNB in cell cycle control and tumorigenic cell invasion via regulation of cyclin E1 and TGF-β2
Source: Genome Biol. 2022 Dec 9;23:252. doi: 10.1186/s13059-022-02800-0 (PMC9733061; doi:10.1186/s13059-022-02800-0)
Supplement: Supplementary file 10 — Additional file 10. Review history. [file 13059_2022_2800_MOESM10_ESM.docx]

Review History

**First round of review**

**Reviewer 1**

**Were you able to assess all statistics in the manuscript, including the appropriateness of statistical tests used?**

Yes, and I have assessed the statistics in my report.

**Comments to author:**

Major Points:

1. Pg6: It is unclear why U2OS line was selected as a model. What is a specific question(s) can be resolved with this line? What is that question? The first sentence comes from nowhere. Please provide a clear explanation.

2. Fig1A: It appears that siRNA-JUNB-803 and 848 affect JUND expression. Please provide quantification of the western blot results.

3. Fig1D: Apparently, there is a significant difference in the EdU incorporation between si-792 vs si-848 (6% vs 18% in EdU incorporation). Please explain the difference and if this is reproducible. In addition, the cell confluency is a critical factor in studies of the cell cycle and may have a confounding effect given a timeframe of the assay (72hr post transfection). It is unclear whether the confluency was indeed controlled in this work.

In addition, the MTS assay measures the cell metabolic activity and can be used for indirect evaluation of cell numbers (proliferation) and cell viability. One can assume that the appropriate correlatives for cell numbers and MTS values have been established prior to the experiment with siRNA. However, it was not mentioned in M&M. Please address this issue.

4. There is a limited overlap in gene expression profiles between the two siRNA-JUNB constructs. If the difference is associated with the level of JUNB protein depletion, then one can expect a variation in the degree of modulation but not in the spectrum of genes. Thus, given ~15% overlap between the two gene sets, these two siRNAs may have off-target effects that may influence to the cell cycle and other biological phenotypes observed in this work. This issue is especially important given overlapping DNA binding activities in JUN-family members, and it should be carefully addressed.

5. The authors should be more specific which E2F factor is discussed in the paper (out of 8 members in human). In respect to RB blots (Fig2D), in asynchronous culture Rb is typically detected in two or more bands (phospho-forms). The blot shows that depletion of JUNB does not affect mobility of RB, while a single band is detected in control conditions. This result does not support the author conclusion, given that CCNE promotes to multi-phosphorylation of RB and late G1-to-S transition. Given a potential significance of the link of JUNB to CCNE1, modulation of CCNE1 and RB proteins should be validated in other cell lines on the study (or even in JUNB-deficient MEFs).

6. It is known that the composition of AP1 factor can influence the activity of AP1 towards a target gene. For example, JUNB can form a complex with FOS or FRA1 proteins that have a stronger transactivating activity compared to JUNB. Thus, although endogenous JUNB and ectopic JUNB can bind to the same genetic element, the outcome may be different due to a composition of AP1. This may explain the data in Fig4E, as in control conditions JUNB levels correlates with an increase in TGFB2, while in the overexpression model the level of TGFB2 is reduced upon JUNB expression. Ectopic JUNB may have a global impact on gene expression and affect cell cycle progression. This notion should be considered in the interpretation of the results in Fig4G and Fig5. Please make corresponding changes in the Results and Discussion.

7. The EMT passages should be avoided as U2OS line represents a mesenchymal lineage. Ectopic JUNB expression enhances TGFb2-induced ECM proteins and integrins. This observation is consistent with prior published reports. In addition, the authors should also assess whether TGFb2 and/or JUNB regulate expression and secretion of other TGFb ligands (b1 and b3). Again, the considerations described in comment 6 should be considered in the interpretation of the results in Fig.6.

8. It is known that tumor size strongly affects metastases. Thus, it is logical that mice with larger tumors show greater metastases. This should be considered in the interpretation of the in vivo data.

9. Correlative analysis of gene expression in human cancers is rather limited and should be performed on larger cohorts of patients related to the study, i.e., sarcoma patients. If breast cancer is considered then use TCGA cohorts (over 3,000 cases).

Minor points:

Pg3Ln60: The first two members, i.e., v-Fos and v-Jun, have been discovered as oncogenes. Cellular FOS/JUNE are considered as protooncogenes. Please rephrase the sentence.

Introduction did not define a specific significant question/gap addressed in the study.

Pg9Ln152: "overexpression of an ectopic JUNB"; This is incorrect use of the term 'ectopic'; Ectopic is overexpression of JUNB, but not JUNB itself, assuming a human JUNB cDNA construct was used. It is better to use 'forced expression' or just 'overexpression of JUNB'.

**Reviewer 2**

**Were you able to assess all statistics in the manuscript, including the appropriateness of statistical tests used?**

Yes, and I have assessed the statistics in my report.

**Comments to author:**

Beatriz Pérez-Benavente et al. report the results of combined transcriptomic, genomic and functional studies showing that JUNB promotes cell cycle progression via induction of cyclin E1 and repression of transforming growth factor (TGF)-b2 genes. They also show that high levels of JUNB switch the response of TGF-b2 stimulation from an anti-proliferative to a pro-invasive one, induce endogenous TGF-b2 production by promoting TGF-b2 mRNA translation, and enhance tumor growth and metastasis in mice. Moreover, tumor genomic data indicate that JUNB amplification associates with poor prognosis in breast and ovarian cancer patients with JUNB amplification. However, JUNB transcription factor involved in the control of many physiological and disease-associated functions. The major limitation of current study is performing most of the experiments in U2OS osteosarcoma cells, but evaluated the clinical relevance in breast and ovarian cancer. The specific comments as followed:

1.The wet experiments in this study should be performed at least in two different types of cell lines. It would be great to select cell line models with clinical relevance.

2.Uncontrolled cell division is one hallmark of cancer. Thus, the roles of JUNB in regulation of cell cycle may contribute to cancer growth and progression. The author should not simply introduce the roles of JUNB in cell cycle and cancer separately.

3.Actually, from Fig1B, siJUNB-848 could also perturb the abundances of the JUN and JUND protein, not only siJUNB-803. More siRNA targeting JUNB should be designed to avoid off-target effects.

4.It would be better to exhibit the G0/G1, S and G2/M distribution of the cells with overexpression of wildtype JUNB, not only show the increased ratio of proliferating cells (fig1e).

5.The overlapped DEGs of U2OS cells transfected with siJUNB-792, siJUNB-848 are limited, compared with the total DEGs. There results indicated the potential off-target effect of the applied siRNAs. Therefore, more siRNAs or samples at different time points will be needed to achieve definite conclusions.

6.In fig2e, knockdown of JUNB also leads to a decrease in total RB protein, not only in phosphorylated RB, how to explain this result?

7.In line 291-293, I don't understand the meaning of this sentence "The other high confidence JUNB target genes without JUNB binding sites, might be either indirect target genes or direct target genes not identified due to the experimental cell growing conditions." The author crossed their ChIP-seq- and the transcriptomic data to get the target genes of JUNB in U2OS cells, but why don't these JUNB target genes have the JUNB binding sites?

8.In the fig4C, the author should show the percentages of the cells in G0/G1, S and G2/M phases?

9.In fig6G, why do the authors use exogenous TGFB2 but not the ligand to perform the polysome-enriched microsomal fraction experiment? In fig 6DEF, they used the TGFB2 ligands.

10.Why does overexpression of JUNB and TGFB2 counteract the antiproliferative activity and promote EMT? Why do different levels of TGFB2 have distinct phenotypes? How much difference in protein levels of TGFB2 leads to the distinct phenotypes? What's the different downstream effectors of TGBF2?

11.In fig7, although liver and lung metastasis were identified by xenograft model, without H&E or tumor cell-specific IHC staining, it was very difficult to define the location of the metastatic tumor cells.

12.It would be better to show the statistic results of the expression of JUNB, CCNE1 and other proteins in the immunofluorescence images in fig7. Furthermore, IHC or western blotting experiments should be done to show the changed protein levels of JUNB, CCNE1 and other proteins in primary tumors and metastases, not only by using immunofluorescence staining.

**Authors Response**

**Point-by-point responses to the reviewers’ comments:**

Reviewer 1

1. Pg6: It is unclear why U2OS line was selected as a model. What is a specific question(s) can be resolved with this line? What is that question? The first sentence comes from nowhere. Please provide a clear explanation.

*Reply: The observation made in our laboratory that JUNB depletion arrested the cell cycle led us to investigate the role of JUNB as a promoter of cell cycle progression, which is poorly studied. Thus, the initial objective of this study was to identify JUNB targets involved in cell cycle regulation in order to deepen into its role as a as cell cycle promoter. For this, the U2OS cells were used, which is a cell model widely used in the literature for cell synchronization assays and cell-cycle studies [1-3]. U2OS are adherent cells with epithelial phenotype, they grow fast and have good transfection efficiencies. In addition, JUNB is expressed in these cells and we had previously used them to study the regulation of JUNB protein levels during cell cycle progression [4, 5]. The consequences of cell cycle arrest after JUNB depletion were also confirmed in HeLa, H1395 and MCF-7 cell lines (Supplemental figure S1).*

*According to the Reviewer’s comment we have added the following sentence in the text:*

*Line 128: “To address the role of JUNB in cell proliferation, we investigated the consequences of JUNB depletion on the distribution of cells in the various phases of the division cycle using U2OS cells since it is a cell model widely used for cell cycle studies and we had previously used them to study the regulation of JUNB protein levels during the G2/M transition.*

2. Fig1A: It appears that siRNA-JUNB-803 and 848 affect JUND expression. Please provide quantification of the western blot results.

*Reply: We have updated Figure 1A and, as suggested by the Reviewer, we provide quantification of western blots results of three different replicates of the experiment in Supplemental Figure 1A. The new data presented confirms that JUN and JUND are significantly affected only when silencing JUNB with siRNA-JUNB-803. According to that we have modified the text as follows:*

*Line 137: Immunoblot analyses showed that siJUNB-803 affected significantly JUN and JUND levels (Figure 1B and Figure S1A). Therefore, all further experiments were conducted with siJUNB-792 and/or siJUNB-848.*

3. Fig1D: Apparently, there is a significant difference in the EdU incorporation between si-792 vs si-848 (6% vs 18% in EdU incorporation). Please explain the difference and if this is reproducible. In addition, the cell confluency is a critical factor in studies of the cell cycle and may have a confounding effect given a timeframe of the assay (72hr post transfection). It is unclear whether the confluency was indeed controlled in this work. In addition, the MTS assay measures the cell metabolic activity and can be used for indirect evaluation of cell numbers (proliferation) and cell viability. One can assume that the appropriate correlatives for cell numbers and MTS values have been established prior to the experiment with siRNA. However, it was not mentioned in M&M. Please address this issue.

*Reply: We appreciate the Reviewer's remark and must apologize as we made a mistake in assembling Figure 1D since the histograms showed corresponded to an EdU assay performed after 96 hours, instead of 72 hours of RNAi transfection as indicated in the figure legend, after JUNB was silenced. According to this we have replaced Figure 1D with new data obtained of EdU assays at 72 h after silencing JUNB. The results show a similar decrease in the EdU incorporation with both JUNB silencers. Results of three independent experiments of the EdU assays are included in the cover letter file (Figure 1).*

*We agree with the Reviewer that cell confluency is a critical factor when studying the cell cycle. For this reason, we carefully monitored cell confluency in our assays. We always seeded the cells at 30% confluency and grown to ~70%-~80% confluency for all experiments. We have specified cell seeding conditions in Material and Methods.*

*Line 719: Cells were seeded at a density of 100,000-150.000 cells per well in a 6-well plate and grown to ~70%-~80% confluency for all experiments.*

*Cell titration assays have been performed prior to the MTS assay to choose the initial cell number plating and to ensure that cell number and absorbance values obtained in the MTS assay are correlative. Find in the cover letter file the corresponding figure not shown in the manuscript (Figure 2). We have added this information in Material and Methods section as follows:*

*Line 757: Cell titration assay was performed to optimize the initial cell number plated and to ensure that cell number and absorbance values obtained in the MTS assay are correlative according to the manufacturer instructions.*

*In addition, new living cell time-lapse imaging experiments have been performed to quantify the proliferation rate of JUNB RNAi transfected U2OS cells and U20S and MCF-7 cells stably overexpressing JUNB. The results obtained confirmed the MTS assays. See new Figure S1D, updated text in Line 158, and supplemental methods, Line 1483.*

4. There is a limited overlap in gene expression profiles between the two siRNA-JUNB constructs. If the difference is associated with the level of JUNB protein depletion, then one can expect a variation in the degree of modulation but not in the spectrum of genes. Thus, given ~15% overlap between the two gene sets, these two siRNAs may have off-target effects that may influence to the cell cycle and other biological phenotypes observed in this work. This issue is especially important given overlapping DNA binding activities in JUN-family members, and it should be carefully addressed.

*Reply: We appreciate the Reviewer's comment about the limited overlap in gene expression profiles between the two siRNA-JUNB silencers. As discussed in the manuscript, siJUNB-848 was less efficient in JUNB depletion, and this could lead to alteration in the degree of modulation of gene expression. This idea is supported by the GSEA data (Figure 2A) showing that the same pathways are found enriched with both JUNB siRNAs. This reinforces the idea that differences between both silencers are more likely due to unequal silencing levels rather than to off-target effects. This has been amended in the text in Line 186.*

*Moreover, we carried out additional experiments to discard the possibility that off-targets could influence on the phenotypes described in this work. For instance, we performed rescue experiments where JUNB was overexpressed in cells where JUNB has been depleted with siRNA. In this way we could partially rescue the cell cycle and proliferation phenotypes observed due to JUNB depletion (Figure S1D). The arrest in the cell cycle after JUNB silencing was confirmed in HeLa, MCF-7 and H1395 cell lines (Figure S1B and C). In addition, we show that the counter-genetic approach (overexpression) lead to opposite phenotypes than the silencing tests. For instance, overexpression of JUNB in U2OS induced proliferation (Fig 1E), acceleration of G1 to S progression (Fig 4C) and induced EMT, cell migration and invasion (Fig 5). New experiments in MCF-7 cells confirmed that overexpression of JUNB induced cell proliferation (Figure S1D, right panel) and EMT (Figure S6A), reinforcing that the influence to the cell cycle and other biological phenotypes observed in this work are JUNB dependent. The text has been updated accordingly in Lines 153 and Line 448.*

5. The authors should be more specific which E2F factor is discussed in the paper (out of 8 members in human). In respect to RB blots (Fig2D), in asynchronous culture Rb is typically detected in two or more bands (phospho-forms). The blot shows that depletion of JUNB does not affect mobility of RB, while a single band is detected in control conditions. This result does not support the author conclusion, given that CCNE promotes to multi-phosphorylation of RB and late G1-to-S transition. Given a potential significance of the link of JUNB to CCNE1, modulation of CCNE1 and RB proteins should be validated in other cell lines on the study (or even in JUNB-deficient MEFs).

*Reply: In the manuscript we referred to the E2F subfamily activators E2F1, E2F2, E2F3. We have added the following sentence to be more specific:*

*Line 208: During the G1/S transition, the kinase CDK2 in complex with basal levels of CCNE1 phosphorylates the retinoblastoma protein (pRB), leading to the activation of the transcription factors E2F1, E2F2 and E2F3, a subclass of E2F transcription factor family.*

*In addition, in the legend of Table S1 we have specified which of the E2F genes are involved as regulators of the indicated target genes.*

*We thank the Reviewer for the comment on Rb blot. To our experience with the commercial antibody used, a single band is detected in the western blots as indicated also by the manufacturer (https://www.cellsignal.com/products/primary-antibodies/rb-4h1-mouse-mab/9309?site-search-type=Products&N=4294956287&Ntt=rb&fromPage=plp). To unequivocally confirm the Rb phosphorylation data, we used another antibody which specifically detects the S795 phosphorylated Rb proteoform as a unique band in the western blot, confirmed also by the manufacturer (https://www.cellsignal.com/products/primary-antibodies/phospho-rb-ser795-antibody/9301?site-search-type=Products&N=4294956287&Ntt=rb&fromPage=plp). Anyhow, we have repeated the experiment in U2OS cells and we have confirmed the results. We have replaced the original figure 2D with a new one with improved loading levels. Moreover, we have confirmed the data obtained with U2OS cells in MCF-7 cell line with identical results (see new figure S2E) and updated text in Line 324.*

6. It is known that the composition of AP1 factor can influence the activity of AP1 towards a target gene. For example, JUNB can form a complex with FOS or FRA1 proteins that have a stronger transactivating activity compared to JUNB. Thus, although endogenous JUNB and ectopic JUNB can bind to the same genetic element, the outcome may be different due to a composition of AP1. This may explain the data in Fig4E, as in control conditions JUNB levels correlates with an increase in TGFB2, while in the overexpression model the level of TGFB2 is reduced upon JUNB expression. Ectopic JUNB may have a global impact on gene expression and affect cell cycle progression. This notion should be considered in the interpretation of the results in Fig4G and Fig5. Please make corresponding changes in the Results and Discussion.

*Reply: We appreciate this comment but, in our opinion, the increased JUNB levels correlate with a decrease in TGFB2 levels. First in Figure 4D we show that enhanced JUNB levels by ectopic expression, lead to reduced TGFB2 mRNA levels during the G1 to S progression. The TGFB2 transcript levels were also confirmed at the protein level in Figure 4E. These gene expression data correlated also with enhanced binding of JUNB to the cis-regulatory elements of TGFB2 (Figure 4F). Moreover, in control conditions of Figure 4E although an initial increase of TGFB2 can be observed from 3 to 9 hours, after that time there is a notable reduction of TGFB2 until 15 hours which correlates with increased JUNB levels. Altogether the data provide sufficient support to our interpretation that JUNB acts as a transcriptional repressor of TGFB2. We agree with the Reviewer that at this point we cannot confirm whether ectopic JUNB is acting as a homodimer or may also recruit other AP1 members to form active heterodimers. To make this clear for the reader, we have added the following sentence in the RESULTS section:*

*Line 364: Although, we cannot discard that overexpression of JUNB may have a global impact on gene expression and affect cell cycle progression, our data indicate that the increased expression of JUNB during progression from G1 to S represses the expression of TGFB2, which would otherwise behave as a cell cycle brake.*

*We have also added the following sentence in DISCUSSION section:*

*line 651: Despite that JUNB overexpression may affect AP-1 dimer composition and widely impact on gene expression and affect cell cycle progression, accelerated G1/S transition triggered by JUNB overexpression correlated with both enhanced JUNB binding at the TGFB2 regulatory elements and lower expression of TGFB2.These data support the role of JUNB role as TGFB2 transcriptional repressor to facilitate proper G1/S cell cycle transition.*

7. The EMT passages should be avoided as U2OS line represents a mesenchymal lineage. Ectopic JUNB expression enhances TGFb2-induced ECM proteins and integrins. This observation is consistent with prior published reports. In addition, the authors should also assess whether TGFb2 and/or JUNB regulate expression and secretion of other TGFb ligands (b1 and b3). Again, the considerations described in comment 6 should be considered in the interpretation of the results in Fig.6.

*Reply: The U2OS cell line, originally known as the 2T line, was established from a moderately differentiated sarcoma of the tibia. During the passages to establish the cell line, the cells gradually assumed an epithelial-like growth pattern [7]. Several studies have demonstrated that U2OS cells underwent alterations associated with the EMT, for example see references [6, 8-10]. Additionally, we have performed new experiments in the MCF-7 breast cancer cell line and confirmed that ectopic JUNB expression enhanced TGFB2-induced EMT proteins and integrins. The results are shown in the new supplementary Figure S6A and the text has been updated accordingly as follows:*

*Line 448: The expression of JUNB and EMT markers were also analysed in established MCF-7 cells expressing JUNB-GFP (MCF-7-JUNB) or GFP (MCF-7-GFP) treated with exogenous TGFB2 with similar results (Figure S6A). MCF-7 cells are positive for epithelial markers, such as E-cadherin. As expected, TGFB2 treatment induced a higher decreased in the expression of E-cadherin in the MCF7-JUNB cells compared to control cells.*

*With regard to the comment saying that TGFB2-induced ECM proteins and integrins by ectopic JUNB expression has already been published, to our knowledge the literature only concerns TGFB1, but not TGFB2. After an exhaustive literature search, we have not been able to find any article describing JUNB-dependent TGFB2 gene expression. Therefore, we believe that the results shown in this manuscript are novel.*

*We appreciate the comment of the Reviewer with regard to other TGFB genes (B1 and B3). We found several data suggesting TGFB1 and TGFB3 gene expression are JUNB-independent. First, we did not find any significant alteration in their gene expression from the JUNB-dependent transcriptomic analysis. In addition, no significant peak of JUNB binding was found in the regulatory regions of either TGFB1 or TGFB3 genes from the Chip-seq analysis. To assess the impact of TGFB2 ligand treatment on their gene expression, we have performed RT-qPCR and western blot analysis for TGFB1 as an example. The results are shown in the new supplementary figure Figures S6B and S6C. In the kinetic analysis of TGFB1 mRNA after TGFB2 ligand treatment only subtle changes were transiently observed. At the protein level, the kinetics of the TGFB1 precursor preprotein and mature monomeric form between control cells and JUNB overexpressing cells were similar, although an increase of TGFB1 protein was observed in the latter cells. The new results are shown in Figure S6B and S6C and the text has been updated accordingly in Lines 496.*

*Taking into account all these data, we conclude that in cells treated with TFGB2 ligand overexpression of JUNB slightly affect the expression of TGFB1 at the transcriptional level, although upon TGFB2 treatment we observed an increase of TGFB1 protein. Therefore, we cannot discard any potential effect of JUNB at the post-transcriptional level.*

*We appreciate the Reviewer’s comments and as in Figure 4 we have amended the text as follows:*

*Line 446: It could be plausible that JUNB overexpression may have a global impact on gene expression by altering AP-1 dimer composition thus promoting EMT.*

8. It is known that tumor size strongly affects metastases. Thus, it is logical that mice with larger tumors show greater metastases. This should be considered in the interpretation of the in vivo data.

*Reply: We have modified the text according to the Reviewer comment as follows:*

*Line 542: These data supported the idea that high JUNB expression confers a stronger tumorigenic potential to U2OS cells, leading to greater metastases.*

9. Correlative analysis of gene expression in human cancers is rather limited and should be performed on larger cohorts of patients related to the study, i.e., sarcoma patients. If breast cancer is considered then use TCGA cohorts (over 3,000 cases).

*Reply: We have performed new correlative analyses of JUNB amplification in breast cancer tumors vs survival with 3055 cases including the Molecular Taxonomy of Breast Cancer International Consortium (METABRIC, n= 1979 patients) and TCGA (Firehose Legacy, n= 1101 patients) cohorts. The new results are show in Figure S10B. For the correlative analysis of protein expression in Figure S11 we used JUNB protein expression data of breast cancer cohorts available in public data bases, which is rather limited.*

*Analysis in osteosarcoma showed that patients with JUNB amplification had a trend of poorer prognosis, however the data is not significative due to the small number of samples and we have not included this result in the manuscript.*

Minor points:

10. Pg3Ln60: The first two members, i.e., v-Fos and v-Jun, have been discovered as oncogenes. Cellular FOS/JUNE are considered as protooncogenes. Please rephrase the sentence.

*Reply: We have replaced the word ‘oncogene’ by ‘protooncogene’ for the cellular AP1 members as suggested by the Reviewer.*

11. Introduction did not define a specific significant question/gap addressed in the study.

*Reply: We have added the following sentence in the Introduction section:*

*Line 103: However, the molecular role of JUNB in tumor cell proliferation and cancer progression is still poorly understood. Therefore, in the present study, we investigated the transcriptional activity of JunB as a promoter of cell proliferation, which let us to propose a novel role of JUNB activity in cell cycle progression.*

12. Pg9Ln152: "overexpression of an ectopic JUNB"; This is incorrect use of the term 'ectopic'; Ectopic is overexpression of JUNB, but not JUNB itself, assuming a human JUNB cDNA construct was used. It is better to use 'forced expression' or just 'overexpression of JUNB'.

*Reply: We appreciate the Reviewer’s comment and we have replaced the term as suggested.*

Reviewer 2

1. The wet experiments in this study should be performed at least in two different types of cell lines. It would be great to select cell line models with clinical relevance.

*Reply: The consequences of cell cycle arrest after JUNB depletion were also confirmed in other cell lines such as HeLa, H1395 and the MCF-7 cell line which is relevant at the clinical level in breast cancer (Supplemental figure S1B and C). We have performed new experiments to confirm the decrease in pRB, CCNE1 and increased of TGFB2 levels after JUNB depletion in MCF-7 cell line (Figure S2E). In addition, new experiments in MCF-7 cells subjected to JUNB RNAi transfection and pCDNA3-JUNB showed that upon JUNB expression rescue, CCNE1 and TGFB2 protein levels paralleled that of control cells (Figure S5A). Furthermore, the effect of JUNB overexpression in the promotion of cell proliferation and EMT have been confirmed in established MCF-7 cells overexpressing JUNB-GFP treated with TFGB2 (Figure S1D right panel and S6A). The text has been updated accordingly in Lines 218, 323, 378 and 448.*

2. Uncontrolled cell division is one hallmark of cancer. Thus, the roles of JUNB in regulation of cell cycle may contribute to cancer growth and progression. The author should not simply introduce the roles of JUNB in cell cycle and cancer separately.

*Reply: As suggested by the Reviewer, we have modified the Introduction section to introduce the roles of JUNB in cell cycle and cancer together.*

*Line 70: It is expressed in numerous cell types and its actions can be ambivalent, not only in cell proliferation control, but also in cancer, as it can be anti- or pro-tumorigenic, depending on the context. JUNB is a cell cycle regulated protein and its abundance varies depending on the cell proliferation state/stage.*

3. Actually, from Fig1B, siJUNB-848 could also perturb the abundances of the JUN and JUND protein, not only siJUNB-803. More siRNA targeting JUNB should be designed to avoid off-target effects.

*Reply: We appreciate Reviewer’s remark and we have updated Figure 1A and provide quantification of western blots results of three different replicates of the experiment in Supplemental Figure 1A. The data presented confirms that JUN and JUND are significantly affected only when silencing JUNB with siRNA-JUNB-803. According to that we have modified the text as follows:*

*Line 137: Immunoblot analyses showed that siJUNB-803 affected significantly JUN and JUND levels (Figure 1B and Figure S1A). Therefore, all further experiments were conducted with siJUNB-792 and/or siJUNB-848.*

*As discussed in the manuscript, siJUNB-848 was less efficient in JUNB depletion, and this could suggest the existence of off-targets. However, according to Figure 2A the GSEA data showed that the same pathways are found enriched with both JUNB siRNAs. This reinforces the idea that differences between both silencers are more likely due to unequal silencing levels rather than to off-target effects. This has been amended in the text in Line186.*

*Moreover, we provide evidences that the phenotypes and molecular pathways affected after siRNA JUNB transfection are not due to off-target effects, since the siRNA-JUNB genetic complementation and the counter-genetic approach by overexpression in different cell lines, demonstrate this robust JUNB-dependency. Some of the identified regulated genes were validated in independent qPCR assays in U2OS (Figure 2C) and in other cell lines such as Hela, H1395 and MCF-7 (Figure S2B, C and D). The arrest in the cell cycle after JUNB depletion was confirmed in HeLa, MCF-7 and H1395 cell lines (Figure S1B and C). To validate the effects of JUNB depletion we have performed new rescue experiments in MCF-7 where JUNB has been depleted with siRNA. As shown in the new Figure S5A, we could recover endogenous CCNE1 and TGFB2 protein expression levels. Moreover, we show that the counter-genetic approach (overexpression) leads to opposite phenotypes than the silencing tests. For instance, overexpression of JUNB in U2OS cells induces proliferation (Figure 1E), acceleration of G1 to S progression (Figure 4C) and induces EMT (Figure 6B and S6A) and cell invasion (Figure 6D). Additionally, new experiments confirmed that JUNB overexpression increased cell proliferation and EMT in MCF-7 cells Figure (S1D right panel and S6A) reinforcing that the influence to the cell cycle, EMT and invasive phenotypes observed in this work are JUNB dependent. the text have been updated accordingly in Lines 153 and 448.*

4. It would be better to exhibit the G0/G1, S and G2/M distribution of the cells with overexpression of wildtype JUNB, not only show the increased ratio of proliferating cells (fig1e).

*Reply: When we transiently overexpress JUNB in exponentially growing cells, no significant changes are observed in the cell cycle after the analysis of DNA content by flow cytometry, therefore we did not include the histogram data. However, when JUNB is conditionally overexpressed in a stable manner (UTA6-JUNB cells) the histograms showed that cells progressed faster through the S-phase of the cycle (Figure 4C). We have added the percentage of G0/G1, S and G2/M cell phase distribution on Figure 4C in the new Figure S4E.*

5. The overlapped DEGs of U2OS cells transfected with siJUNB-792, siJUNB-848 are limited, compared with the total DEGs. There results indicated the potential off-target effect of the applied siRNAs. Therefore, more siRNAs or samples at different time points will be needed to achieve definite conclusions.

*Reply: As pointed in answer 3 the results of the GSEA data confirmed that the same pathways are enriched with both JUNB siRNAs suggesting that differences between both silencers are more likely due to unequal silencing levels rather than to off-target effects. We also provide evidences that the limited overlap between the two siRNA-JUNB constructs is not relevant for the phenotypes and molecular pathways affected, since the siRNA-JUNB genetic complementation and the counter-genetic approach by overexpression in different cell lines, demonstrate the robust JUNB-dependency. For instance, several of the identified regulated genes have been validated in independent qPCRs in U2OS and in other cell lines such as HeLa, H1395 and MCF-7 (Fig S2B, C, D). Rescue experiments discard that the observed phenotypes are due to off-target effects (Figure S1C and S5A). Moreover, the overexpression of JUNB in U2OS and MCF-7 cell lines, lead to opposite phenotypes and induced cell proliferation and EMT, thus reinforcing the influence of JUNB in the cell cycle and other biological phenotypes observed in this work.*

6. In fig2e, knockdown of JUNB also leads to a decrease in total RB protein, not only in phosphorylated RB, how to explain this result?

*Reply: We have replaced the original Figure 2E with a new one with improved loading levels and the data shows that total Rb protein levels do not decrease. Moreover, we have confirmed the data obtained with U2OS cells in MCF-7 cell line transfected with siJUNB with the same results (see new figure S2E). The text has been updated accordingly in Line 378.*

7. In line 291-293, I don't understand the meaning of this sentence "The other high confidence JUNB target genes without JUNB binding sites, might be either indirect target genes or direct target genes not identified due to the experimental cell growing conditions." The author crossed their ChIP-seq- and the transcriptomic data to get the target genes of JUNB in U2OS cells, but why don't these JUNB target genes have the JUNB binding sites?

*Reply: Reviewer’s remark is right. Instead of “high confidence JUNB target genes” it should say “high-confidence JUNB-regulated genes”. The genes whose expression varied upon JUNB depletion by the two JUNB siRNAs were called high-confidence JUNB-regulated genes. After crossing our ChIP-seq- with the transcriptomic data we identified that some of these regulated genes have JUNB binding sites and others do not. Therefore, the genes whose expression are altered after JUNB silencing but were not identified in the Chip-seq analysis could be indirect target genes.*

*We apologize for this error in the phrase that has created confusion and have corrected as follows:*

*Line 301: The other high confidence JUNB-regulated genes identified in the transcriptomic assay without JUNB binding sites could be indirect target genes.*

8. In the fig4C, the author should show the percentages of the cells in G0/G1, S and G2/M phases?

*Reply: As suggested by the Reviewer, we have included the histograms with the percentages of the cells in G0/G1, S and G2/M phases in Figure S4E.*

9. In fig6G, why do the authors use exogenous TGFB2 but not the ligand to perform the polysome-enriched microsomal fraction experiment? In fig 6DEF, they used the TGFB2 ligands.

*Reply: We apologize for the misunderstanding. Indeed, we had used TGFB2 ligand to perform the polysome-enriched microsomal fraction experiment. We have replaced the word ‘exogenous’ for ‘ligand’ in Line 1269 to avoid confusion.*

10. Why does overexpression of JUNB and TGFB2 counteract the antiproliferative activity and promote EMT? Why do different levels of TGFB2 have distinct phenotypes? How much difference in protein levels of TGFB2 leads to the distinct phenotypes? What's the different downstream effectors of TGBF2?

*Reply: The explanation to the question raised by the Reviewer was schematically shown in Figure 6H. To summarize it, our data suggest that JUNB overexpressed cells display enhanced proliferation and, after a long stimulation with TGFB2 ligand, undergo an additional increase in JUNB expression (Figure 6B) which in turn enhances EMT gene expression (Figure 6B) and TGFB2 translation (Figure 6G). This positive feedback eventually leads to enhanced proliferation (Figure 6A) and also enhanced invasion (Figure 6D).*

*We have only tested different times of exposure of cells to TGFB2 and observed distinct phenotypes. The data obtained suggest that a sustained activated TGFB2 signalling in cells overexpressing JUNB will lead to an aggressive phenotype. We have not assayed different amounts of TGFB2.*

*It would be also interesting to identify the different downstream effectors of TGFB2 in each cell condition, but this is beyond the scope of this manuscript. As we point in the discussion in Line 656, future work will have to elucidate the intimate molecular mechanisms involved.*

11. In fig7, although liver and lung metastasis were identified by xenograft model, without H&E or tumor cell-specific IHC staining, it was very difficult to define the location of the metastatic tumor cells.

*Reply: We appreciate the Reviewer's remark and we have performed H&E, and IHC staining with anti GFP to detect tumor specific cells. The new data included in Figure S7A shows the identification of cancer cells in the primary tumor and metastatic cells in the liver and lung with H/E staining. Images of tumor cell-specific GFP expression obtained by IHC are shown in primary tumors (Figure S7B) and in liver and lung metastasis (Figure S7B and S7C). We have updated the Results and Supplemental methods sections accordingly (see Lines 534, 541, 544 to 563).*

12. It would be better to show the statistic results of the expression of JUNB, CCNE1 and other proteins in the immunofluorescence images in fig7. Furthermore, IHC or western blotting experiments should be done to show the changed protein levels of JUNB, CCNE1 and other proteins in primary tumors and metastases, not only by using immunofluorescence staining.

*Reply: We have performed new analysis of JUNB, CCNE1, TGFB2, Fibronectin and Integrin B1 protein expression in primary tumors and metastasis by IHC and confirmed the results obtained with immunofluorescence staining presented in Figure 7. The new figures are shown in Figure S9. The text has been updated in Line 559.*

*1. Hao Q, Zong X, Sun Q, Lin YC, Song YJ, Hashemikhabir S, et al. The S-phase-induced lncRNA SUNO1 promotes cell proliferation by controlling YAP1/Hippo signaling pathway. Elife. 2020;9.*

*2. Wang P, Hua X, Bryner YH, Liu S, Gitter CB, Dai J. Haspin inhibition delays cell cycle progression through interphase in cancer cells. J Cell Physiol. 2020;235(5):4508-19.*

*3. Mitxelena J, Apraiz A, Vallejo-Rodriguez J, Malumbres M, Zubiaga AM. E2F7 regulates transcription and maturation of multiple microRNAs to restrain cell proliferation. Nucleic Acids Res. 2016;44(12):5557-70.*

*4. Farràs R, Baldin V, Gallach S, Acquaviva C, Bossis G, Jariel-Encontre I, et al. JunB breakdown in mid-/late G2 is required for down-regulation of cyclin A2 levels and proper mitosis. Mol Cell Biol. 2008;28(12):4173-87.*

*5. Perez-Benavente B, Garcia JL, Rodriguez MS, Pineda-Lucena A, Piechaczyk M, Font de Mora J, et al. GSK3-SCF(FBXW7) targets JunB for degradation in G2 to preserve chromatid cohesion before anaphase. Oncogene. 2013;32(17):2189-99.*

*6. Wang Y, Ren X, Yuan Y, Yuan BS. Downregulated lncRNA GAS5 and Upregulated miR-21 Lead to Epithelial-Mesenchymal Transition and Lung Metastasis of Osteosarcomas. Front Cell Dev Biol. 2021;9:707693.*

*7. Ponten J, Saksela E. Two established in vitro cell lines from human mesenchymal tumours. Int J Cancer. 1967;2(5):434-47.*

*8. Fukuda S, Akiyama M, Harada H, Nakahama KI. Effect of gap junction-mediated intercellular communication on TGF-beta induced epithelial-to-mesenchymal transition. Biochem Biophys Res Commun. 2019;508(3):928-33.*

*9. Yang J, Guo W, Wang L, Yu L, Mei H, Fang S, et al. Notch signaling is important for epithelial-mesenchymal transition induced by low concentrations of doxorubicin in osteosarcoma cell lines. Oncol Lett. 2017;13(4):2260-8.*

*10. Sung JY, Park SY, Kim JH, Kang HG, Yoon JH, Na YS, et al. Interferon consensus sequence-binding protein (ICSBP) promotes epithelial-to-mesenchymal transition (EMT)-like phenomena, cell-motility, and invasion via TGF-beta signaling in U2OS cells. Cell Death Dis. 2014;5:e1224.*

**Second round of review**

**Reviewer 2**

All the comments of results part have been addressed. Despite the author present amount of information in the introduction section, it is still hard to pull out the key conclusions or questions addressed in the study. Moreover, the manuscript would benefit from simplifying concepts and improved organization.

I also went through the point-to-point response to reviewer 1, and the major concerns are shared by the two reviewers. The author has addressed the concerns from reviewer 1. I thought it will be ready for publication with editorial editing.

**Authors Response**

**Point-by-point responses to the reviewers’ comments:**

Reviewer 2

All the comments of results part have been addressed. Despite the author present amount of information in the introduction section, it is still hard to pull out the key conclusions or questions addressed in the study. Moreover, the manuscript would benefit from simplifying concepts and improved organization.

I also went through the point-to-point response to reviewer 1, and the major concerns are shared by the two reviewers. The author has addressed the concerns from reviewer 1. I thought it will be ready for publication with editorial editing.

*Reply: We have modified the last paragraph of the Introduction and the Conclusions sections to highlight the questions addressed in the study and to clarify the text.*
